# Bioinspired design of redox-active ligands for multielectron catalysis: effects of positioning pyrazine reservoirs on cobalt for electro- and photocatalytic generation of hydrogen from water†
†Electronic supplementary information (ESI) available: Non-aqueous cyclic voltammetry; Levich plots and scan rate dependence of aqueous voltammetry; pH dependence of photocatalysis; computational details; and supporting figures. CCDC 1060291–1060296. For ESI and crystallographic data in CIF or other electronic format see DOI: 10.1039/c5sc01414j



**DOI:** 10.1039/c5sc01414j

**Published:** 2015-06-09

**Authors:** Jonah W. Jurss, Rony S. Khnayzer, Julien A. Panetier, Karim A. El Roz, Eva M. Nichols, Martin Head-Gordon, Jeffrey R. Long, Felix N. Castellano, Christopher J. Chang

**Affiliations:** a Department of Chemistry , University of California , Berkeley , California 94720 , USA . Email: chrischang@berkeley.edu ; Email: jrlong@berkeley.edu ; Email: mhg@cchem.berkeley.edu; b Department of Molecular and Cell Biology , University of California , Berkeley , California 94720 , USA; c Department of Chemistry and Biochemistry , University of Mississippi , University , MS 38677 , USA; d Materials Sciences Division , Lawrence Berkeley National Laboratory , Berkeley , California 94720 , USA; e Chemical Sciences Division , Lawrence Berkeley National Laboratory , Berkeley , California 94720 , USA; f Department of Chemistry , North Carolina State University , Raleigh , NC 27695-8204 , USA . Email: fncastel@ncsu.edu; g Department of Natural Sciences , Lebanese American University , Beirut 1102-2801 , Chouran , Lebanon; h Howard Hughes Medical Institute , University of California , Berkeley , California 94720 , USA

## Abstract

Mononuclear metalloenzymes in nature can function in cooperation with precisely positioned redox-active organic cofactors in order to carry out multielectron catalysis. Inspired by the finely tuned redox management of these bioinorganic systems, we present the design, synthesis, and experimental and theoretical characterization of a homologous series of cobalt complexes bearing redox-active pyrazines. These donor moieties are locked into key positions within a pentadentate ligand scaffold in order to evaluate the effects of positioning redox non-innocent ligands on hydrogen evolution catalysis. Both metal- and ligand-centered redox features are observed in organic as well as aqueous solutions over a range of pH values, and comparison with analogs bearing redox-inactive zinc(ii) allows for assignments of ligand-based redox events. Varying the geometric placement of redox non-innocent pyrazine donors on isostructural pentadentate ligand platforms results in marked effects on observed cobalt-catalyzed proton reduction activity. Electrocatalytic hydrogen evolution from weak acids in acetonitrile solution, under diffusion-limited conditions, reveals that the pyrazine donor of axial isomer **1-Co** behaves as an unproductive electron sink, resulting in high overpotentials for proton reduction, whereas the equatorial pyrazine isomer complex **2-Co** is significantly more active for hydrogen generation at lower voltages. Addition of a second equatorial pyrazine in complex **3-Co** further minimizes overpotentials required for catalysis. The equatorial derivative **2-Co** is also superior to its axial **1-Co** congener for electrocatalytic and visible-light photocatalytic hydrogen generation in biologically relevant, neutral pH aqueous media. Density functional theory calculations (B3LYP-D2) indicate that the first reduction of catalyst isomers **1-Co**, **2-Co**, and **3-Co** is largely metal-centered while the second reduction occurs at pyrazine. Taken together, the data establish that proper positioning of non-innocent pyrazine ligands on a single cobalt center is indeed critical for promoting efficient hydrogen catalysis in aqueous media, akin to optimally positioned redox-active cofactors in metalloenzymes. In a broader sense, these findings highlight the significance of electronic structure considerations in the design of effective electron–hole reservoirs for multielectron transformations.

## Abstract 2

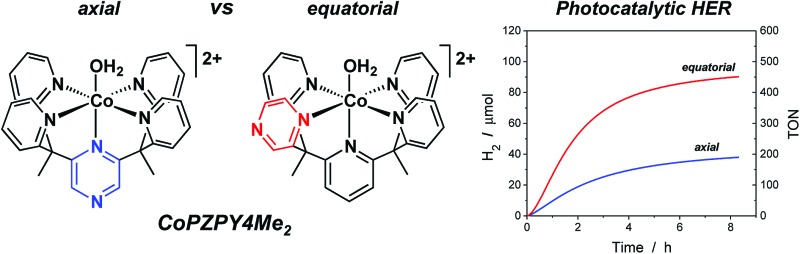
We report the effects of installing redox-active pyrazines at distinct positions in a series of isostructural Co catalysts.

## Introduction

Rising global energy demands and climate change provide motivation to develop new approaches for solar-to-fuel conversion chemistry.^1^ In this context, hydrogen is an attractive energy-dense, carbon-free fuel that is accessible by the two-electron reduction of water and thus a target product of many schemes for artificial photosynthesis.^2^ Numerous molecular catalysts for hydrogen evolution have been described, including ones that rely on earth-abundant metals, but the vast majority of these systems require organic acids, solvents, and/or other additives.^3^ In contrast, hydrogen-evolving catalysts that can reduce protons directly from water, particularly at environmentally-benign neutral pH values to avoid organic additives and corrosive conditions, remain rare and examples based on Co,^4–22^ Ni,^23–30^ Fe,^31–34^ and Mo^35,36^ have been reported. In previous work, we have leveraged the coordination chemistry of polypyridine ligand platforms to develop molecular hydrogen-evolving catalysts that can operate under biologically-compatible conditions (pH 7 buffered water and seawater),^10,17,20,35^ that structurally and functionally mimic active edge-sites in extended materials such as MoS_2_,^36^ and which can be driven by photoredox catalysis with molecular [Ru(bpy)_3_]^2+^ or semiconducting GaP nanowire chromophores.^10,17,20^

**Fig. 1:**
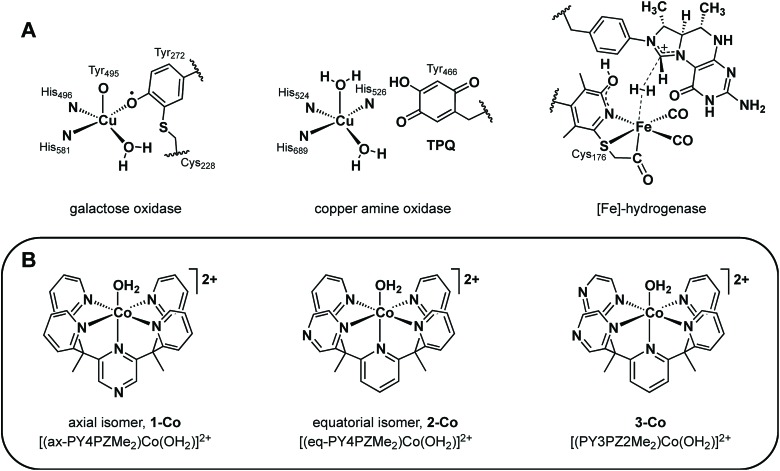
(A) Mononuclear metalloenzyme active sites with optimally positioned redox non-innocent organic cofactors. (B) A homologous series of molecular cobalt catalysts for water reduction to H_2_ containing pyrazine donors in a PY5Me_2_-type framework.

In search of new design strategies for the two-electron reduction of water to hydrogen, we were attracted to the integral role and ubiquity of redox-active ligands in numerous biological systems. Metalloenzymes routinely perform multielectron reactions near thermodynamic potentials under physiological conditions by accumulating multiple redox equivalents over proximal sites involving ligated or adjacent redox-active cofactors.^37–43^ Such redox-active moieties have finely tuned potentials and are optimally positioned within metalloenzyme active sites to promote synergistic redox chemistry. Of particular interest are systems comprising a single metal active site that functions in concert with redox-active organic pendants to execute multielectron transformations.^37–43^ Prototypical enzymes of this class (Fig. 1A) include galactose oxidase (GO) which catalyzes the two-electron conversion of primary alcohols to aldehydes *via* cooperative oxidation by a Cu(ii) center and coordinated phenoxyl radical,^38,39^ copper amine oxidase (CAO) which utilizes an *o*-quinone moiety (TPQ) to catalyze two half reactions in route to transforming primary amines to aldehydes,^40,43^ and mononuclear iron hydrogenase, comprising a tautomeric 2-hydroxypyridine/pyridone ligand and a closely-spaced, redox-active pterin cofactor that together enable efficient hydrogen processing.^41,42^

More specifically, the phenoxyl radical of GO is localized on an unusual cysteine-modified tyrosine residue whose in-plane orientation, combined with its axial/equatorial coordination to copper allows antiferromagnetic coupling with the metal, promotes resonance stabilization of the radical.^39^ In CAO, the versatility and optimal activity of this enzyme emerges from the carefully regulated position and orientation of the TPQ ring for controlled interaction with the metal.^40^ Similarly, the activity of iron hydrogenase is critically linked to the interplay of the redox non-innocent pyridyl unit and pterin cofactor at the interface of a labile iron coordination site.^41,42^ Indeed, precise arrangement of first- and second-sphere redox components is required in enzymatic systems and is most clearly observed in the diminished activity of minimally-modified active sites by protein engineering^40b,42,44^ and of less optimized synthetic model systems.^39,41d,e^

Intrigued by the exquisite management of redox inventories in these metalloenzymes, we sought to prepare synthetic catalysts bearing redox-active moieties to catalytically produce hydrogen from water and to further understand the electronic structure–function relationships of these redox reservoirs in catalysis. Specifically, we targeted the incorporation of redox-active donors at key positions within a structurally well-defined cobalt pentapyridine motif with demonstrated hydrogen production activity and focused on pyrazine as an isostructural pyridine analog. Because the gas phase electron affinity of pyrazine is *ca.* 0.6 eV more positive than pyridine,^45,46^ it can be reduced at modest potentials^47^ and could serve as a redox-active component to facilitate the two-electron reduction of protons to hydrogen. Moreover, we reasoned that the lower lying π* orbitals of pyrazine relative to pyridine would enhance metal-to-ligand backbonding from the cobalt center and give a more electron-deficient metal with more positive reduction potentials.^48,49^ Additionally, we note that seminal observations of redox non-innocent ligand behavior in metal dithiolene complexes^12,50^ have spawned a rich vein of inorganic reactivity studies in the area of redox-active ligands.^51–54^

In this report, we present the synthesis and characterization of a homologous series of cobalt complexes supported by pentadentate ligands where redox-active pyrazine functionalities are systematically incorporated at axial and equatorial positions (Fig. 1B). These bioinspired systems are capable of electro- and photocatalytic production of hydrogen from water at neutral pH. Catalyst isomers display markedly different reactivities depending on the relative position of the non-innocent pyrazine moiety, with a *ca.* 200 mV improvement in overpotential compared to pyridine analogs that lack pendant redox reservoirs. Density functional calculations have been performed to investigate the cobalt complexes and their one- and two-electron reduced species. This further illustrates the importance of electronic structure considerations in the placement of redox non-innocent ligands relative to catalytic metal sites for multielectron chemistry.

## Experimental section

### Materials and methods

Unless otherwise noted, all synthetic manipulations were carried out under a nitrogen atmosphere in a Vacuum Atmospheres glovebox or using Schlenk techniques. Tetrahydrofuran, acetonitrile, 1,2-dimethoxyethane, and dichloromethane were dried *via* Vacuum Atmospheres solvent purification system. The compounds 2-ethylpyridine, 2-fluoropyridine, 2,6-difluoropyridine, 2-ethylpyrazine, 2-chloropyrazine, 2,6-dichloropyrazine were purchased from Oakwood Chemicals. Anhydrous zinc(ii) trifluoromethanesulfonate (Zn(OTf)_2_) and anhydrous cobalt chloride (CoCl_2_) were purchased from Strem Chemicals. Trimethylsilyl trifluoromethanesulfonate (TMS triflate), tetrabutylammonium hexafluorophosphate (Bu_4_NPF_6_), tris(2,2′-bipyridyl)ruthenium(ii) chloride hexahydrate (Ru(bpy)_3_Cl_2_·6H_2_O), *n*-butyllithium (2.5 M solution), lithium diisopropylamide (1.8 or 2.0 M solution), anhydrous 1,4-dioxane, and l-ascorbic acid were purchased from Sigma Aldrich. Glassy carbon rods (type 2) were purchased from Alfa Aesar for electrochemical studies. Water was purified with the Millipore Milli-Q UF Plus system. All other chemical reagents were purchased from commercial vendors and used without further purification. ^1^H and ^13^C NMR spectra were obtained using Bruker spectrometers operating at 400 MHz (^1^H) or 100 MHz (^13^C) as noted. Spectra were calibrated to residual protiated solvent peaks; chemical shifts are reported in ppm. Positive mode, high-resolution electrospray ionization mass spectra (HR-ESI-MS) were obtained from the QB3/College of Chemistry Mass Spectrometry Facility and elemental analysis of carbon, hydrogen, and nitrogen were obtained from the Microanalytical Laboratory, both at the University of California, Berkeley.

### Electrochemical measurements

Electrochemistry was performed with a Bioanalytical Systems, Inc. (BASi) Epsilon potentiostat equipped with a BASi RDE-2 cell stand for rotating disk electrode voltammetry experiments. Voltammetry studies employed a three-electrode cell equipped with glassy carbon disc (3 mm dia.) working electrode, platinum wire counter electrode, and Ag/AgCl (3.0 M NaCl) aqueous reference or silver wire quasi-reference electrode for nonaqueous experiments conducted in anhydrous acetonitrile containing 0.1 M Bu_4_NPF_6_ electrolyte. Voltammograms in acetonitrile were referenced using ferrocene as an internal standard. Note: all voltammograms were cycled from the most positive potential to the most negative potential and back. Controlled potential electrolyses in acetonitrile with chloroacetic acid were conducted in a two-compartment H-cell with a glassy carbon disc (3 mm dia.) working electrode with a Ag/AgCl reference electrode in one side, separated from the other compartment, containing a platinum counter electrode, by a medium-porosity glass frit. Controlled potential electrolyses in aqueous phosphate buffer were conducted in similar two-compartment H-cells, but with a Hg pool working electrode (surface area = 19.6 cm^2^). Constant stirring was maintained during controlled potential electrolysis experiments. Evolved H_2_ during electrolysis measurements was quantified by gas chromatographic analysis of headspace gases using a Varian Micro-GC with a Molecular Sieve 5 Å column (length of 40 m). Integrated gas peaks were quantified with a calibration curve using 5 mL of injected methane as an internal standard. Values are plotted against the theoretical (assuming 100% Faradaic efficiency) hydrogen volume based on the accumulated charge passed during electrolysis.

### X-ray crystallography

Single crystals were coated with Paratone-N hydrocarbon oil and mounted on Kaptan loops. Temperature was maintained at 100 K with an Oxford Cryostream 700 during data collection at the University of California, Berkeley, College of Chemistry, X-ray Crystallography Facility. Samples were irradiated with Mo-Kα radiation with *λ* = 0.71073 Å using a Bruker APEX II QUAZAR diffractometer equipped with a Microfocus Sealed Source (Incoatec IμS) and APEX-II detector or a Bruker SMART APEX diffractometer equipped with a Fine-Focus Sealed Source and APEX-I detector. The Bruker APEX2 v. 2009.1 software package was used to integrate raw data which were corrected for Lorentz and polarization effects.^55^ A semi-empirical absorption correction (SADABS) was applied.^56^ Space groups were identified based on systematic absences, E-statistics, and successive refinement of the structures. The structures were solved using direct methods and refined by least-squares refinement on *F* ^2^ and standard difference Fourier techniques using SHELXL.^57^ Thermal parameters for all non-hydrogen atoms were refined anisotropically, and hydrogen atoms were included at ideal positions and refined isotropically.

### Computational methods

Density functional theory (DFT) calculations were performed with the Q-Chem package^58^ using the B3LYP functional.^59,60^ The def2-TZVP basis set^61^ was employed for Co and Zn while the def2-SVP basis set^61^ was used for all other atoms (denoted as BS1). Single-point calculations using diffuse functions at the B3LYP-optimized geometries were also performed. In this case, the def2-TZVPD basis set^61^ was employed for Co and Zn while the def2-SVPD basis set^61^ was used for all other atoms (denoted as BS2). The X-ray structure of each cation of Co-aqua salts reported herein was used as the initial input for calculations. However, in order to interpret the electrochemistry data in acetonitrile, the axial water molecule was substituted by CH_3_CN. Exchange correlation integrals were evaluated with a quadrature grid of 75 radial points and 302 Lebedev angular points. Unrestricted SCF calculations were performed using tight criterion and either the Direct Inversion in the Iterative Subspace (DIIS) algorithm^62^ or the Geometric Direct Minimization (GDM) algorithm^63^ with an integral threshold of 10^–14^ Hartrees and a convergence criterion of 10^–8^ Hartrees. Stability analyses were performed in addition to analytical frequency calculations on all stationary points to ensure that geometries correspond to local minima (no negative eigenvalue). The solvation effect was included *via* the SWIG C-PCM approach^64^ (water, *ε* = 78.355; acetonitrile, *ε* = 37.219) using the UFF radii as well as dispersion-corrected DFT using Grimme's D2 parameter set^65^ at the B3LYP-optimized geometries. In addition, all reported energies are corrected for zero-point-vibrational energy, while free energies (quoted at 298.15 K and 1 atm) are corrected for the harmonic oscillator approximation proposed by Grimme where low-lying vibrational modes are treated by a free-rotor approximation (see ESI†).^66^

### Photocatalysis experiments

In a 16-well combinatorial apparatus previously described,^17,67^ a 10 mL aqueous solution volume prepared in a 20 or 40 mL vial fitted to a Teflon reactor, which is connected to a pressure transducer (p51 pressure sensor, SSI technologies) and a mass spectrometer (UGA-Hydrogen, SRS) through capillary tubes. Solutions containing ascorbic acid/ascorbate (prepared by titration of ascorbic acid in water with NaOH) and the photosensitizer were thoroughly deaerated using 10 vacuum/argon pressurization cycles. The molecular cobalt catalysts were introduced under inert atmosphere and degassing was continued; finally terminated by equilibration to atmospheric pressure. Each solution was irradiated from the bottom using royal-blue Philips LEDs with an optical power output at *λ*_max_ = 452 ± 10 nm of 540 mW. All experiments were performed at a constant rotation speed of 150 rpm and constant temperature of 20 °C. The quantification of H_2_ was performed through normalizing the processed pressure transducer data to the calculated moles of H_2_ produced, which was measured independently by gas chromatography (GC-8A, Shimadzu) and mass spectrometry.

### Synthetic precursors

Literature procedures were used for the preparation of 2,2′-(ethane-1,1-diyl)dipyridine,^68^ 2,2′-(1-(6-fluoropyridin-2-yl)ethane-1,1-diyl)dipyridine (PY3MeF),^69^ and cobalt(ii)bis(acetonitrile)bis(trifluoromethanesulfonate) (Co(CH_3_CN)_2_(OTf)_2_).^70^

#### 2,6-Bis(1,1-di(pyridin-2-yl)ethyl)pyrazine (ax-PY4PZMe_2_), (**1**)

To a 2-neck round bottom flask equipped with reflux condenser was added 2,2′-(ethane-1,1-diyl)dipyridine (6.06 g, 32.9 mmol) in 50 mL of anhydrous tetrahydrofuran, which was then cooled to –78 °C. A 2.0 M solution of LDA (17.3 mL, 34.5 mmol, 1.05 equivalents) was added by syringe and the solution was allowed to warm to room temperature. Next, 2,6-dichloropyrazine (1.23 g, 8.26 mmol, 0.25 equivalents) was added to the reaction mixture and it was refluxed at 90 °C for 2.5 days. Upon cooling to room temperature, residual LDA was quenched with excess water, and organics were extracted with diethyl ether. The extract was dried over anhydrous sodium sulfate, the solvent was removed by rotary evaporation, and purification was achieved by alumina gel chromatography eluting with 1 : 1 ethyl acetate : hexanes. Pure product was obtained as the last band off the column to yield an off-white solid, 3.23 g (88%). ^1^H NMR (CDCl_3_, 400 MHz): *δ* 8.53 (ddd, *J* = 4.9, 1.9, 0.9 Hz, 4H), 8.30 (s, 2H), 7.48 (td, *J* = 7.8, 1.9 Hz, 4H), 7.09 (ddd, *J* = 7.5, 4.8, 1.1 Hz, 4H), 6.94 (dt, *J* = 8.0, 1.1 Hz, 4H), 2.22 (s, 6H). ^13^C NMR (CDCl_3_, 101 MHz): *δ* 165.11 (s), 159.21 (s), 148.71 (s), 141.61 (s), 135.91 (s), 123.59 (s), 121.36 (s), 58.83 (s), 26.57 (s). HR-ESI-MS (M^+^) *m*/*z* calc. for [**1** + H^+^], 445.2135, found, 445.2135.

#### 2-(1-(Pyridin-2-yl)ethyl)pyrazine, (**2a**)

To a 2-neck round bottom flask equipped with reflux condenser was added 2-ethylpyridine (7.00 g, 7.47 mL, 65.3 mmol) in 75 mL anhydrous 1,2-dimethoxyethane, which was cooled to 0 °C before a 2.5 M solution of *n*-butyllithium (26.1 mL, 65.3 mmol, 1 equivalent) was added by syringe. The reaction mixture was allowed to react for 15 min before 2-chloropyrazine (3.74 g, 2.92 mL, 32.7 mmol, 0.5 equivalents) was added by syringe. The reaction was refluxed overnight at 105 °C. After allowing the reaction to cool to room temperature, it was quenched with a copious amount of water and extracted with diethyl ether. The organic phase was dried over anhydrous sodium sulfate and subsequently taken to dryness on a rotary evaporator. Purification was achieved by vacuum distillation where unreacted starting material and side-product, butyl-substituted chloropyrazine (resulting from *n*-butyllithium addition to the pyrazine ring) were distilled first, followed by product at elevated temperatures (∼150 °C) to yield a pure yellow oil, 4.0 g (66%). ^1^H NMR (CDCl_3_, 300 MHz): *δ* 8.57 (d, *J* = 1.4 Hz, 1H), 8.55–8.50 (m, 1H), 8.49 (dd, *J* = 2.7, 1.5 Hz, 1H), 8.38 (d, *J* = 2.5 Hz, 1H), 7.61 (td, *J* = 7.7, 1.9 Hz, 1H), 7.28 (dt, *J* = 7.9, 1.1 Hz, 1H), 7.12 (ddd, *J* = 7.5, 4.8, 1.2 Hz, 1H), 4.48 (q, *J* = 7.2 Hz, 1H), 1.76 (d, *J* = 7.2 Hz, 3H). ^13^C NMR (CDCl_3_, 101 MHz): *δ* 162.28 (s), 158.89 (s), 149.03 (s), 144.24 (s), 143.56 (s), 142.15 (s), 136.41 (s), 121.94 (s), 121.48 (s), 47.17 (s), 19.11 (s). HR-ESI-MS (M^+^) *m*/*z* calc. for [**2a** + H^+^], 186.1026, found, 186.1028.

#### 2-(1-(6-(1,1-Di(pyridin-2-yl)ethyl)pyridin-2-yl)-1-(pyridin-2-yl)ethyl)pyrazine, (eq-PY4PZMe_2_, **2**)

To a 2-neck round bottom flask equipped with reflux condenser was added compound **2a** (1.34 g, 7.23 mmol) and 50 mL anhydrous 1,2-dimethoxyethane, which was then cooled to 0 °C. Next, a 1.8 M solution of LDA (4.2 mL, 7.6 mmol, 1.05 equivalents) was added by syringe and the reaction was left to stir for 15 min before the addition of PY3MeF (1.01 g, 3.6 mmol, 0.5 equivalents). The reaction mixture was heated to reflux at 105 °C for 2 days and then cooled to room temperature, quenched with excess water, and extracted with diethyl ether. The organic phase was dried over anhydrous sodium sulfate and solvent was removed *via* rotary evaporation. Alumina gel chromatography was employed, eluting with 1 : 1 ethyl acetate : hexanes with the second spot off the column yielding pure ligand as an off-white solid, 1.433 g (89%). ^1^H NMR (CDCl_3_, 400 MHz): *δ* 8.51 (dt, *J* = 4.7, 2.2 Hz, 3H), 8.40 (dd, *J* = 1.2, 1.2 Hz, 1H), 8.28 (d, *J* = 2.7 Hz, 1H), 8.15 (d, *J* = 1.2 Hz, 1H), 7.60 (t, *J* = 7.9 Hz, 1H), 7.51–7.38 (m, 3H), 7.14 (d, *J* = 7.9 Hz, 1H), 7.13–7.02 (m, 4H), 6.83 (td, *J* = 9.3, 8.6, 6.3 Hz, 3H), 2.20 (s, 3H), 2.18 (s, 3H). ^13^C NMR (CDCl_3_, 101 MHz): *δ* 166.08 (s), 165.17 (s), 164.85 (s), 163.16 (s), 162.01 (s), 148.78 (s), 148.58 (s), 146.72 (s), 142.62 (s), 141.28 (s), 137.06 (s), 135.97 (s), 135.71 (s), 123.88 (s), 123.65 (s), 121.43 (s), 121.14 (s), 120.46 (s), 119.65 (s), 60.15 (s), 58.81 (s), 26.92 (s), 26.33 (s). HR-ESI-MS (M^+^) *m*/*z* calc. for [**2** + H^+^], 445.2135, found, 445.2132.

#### 2,2′-(Ethane-1,1-diyl)dipyrazine, (**3a**)

To a 2-neck round bottom flask equipped with reflux condenser was added 2-ethylpyrazine (11.0 g, 11.2 mL, 102 mmol) in 75 mL of anhydrous tetrahydrofuran, which was cooled to –78 °C, before a 1.8 M solution of lithium diisopropylamide, LDA, (59.5 mL, 107 mmol, 1.05 equivalents) was added *via* cannula transfer. The reaction mixture was allowed to stir for 15 min before it was warmed to room temperature. Then 2-chloropyrazine (5.84 g, 4.55 mL, 51.0 mmol) was added by syringe. The reaction was refluxed at 90 °C for 2 days. Upon cooling to room temperature, residual LDA was quenched with a copious amount of water. The product was extracted into diethyl ether, and dried over anhydrous sodium sulfate. The crude product was taken to dryness on a rotary evaporator and purified by vacuum distillation. Unreacted starting material came off first followed by the product at elevated temperatures (∼150 °C) to yield a pure yellow oil, 6.64 g (70%). ^1^H NMR (CDCl_3_, 400 MHz): *δ* 8.61 (d, *J* = 1.5 Hz, 2H), 8.49 (dd, *J* = 2.6, 1.6 Hz, 2H), 8.42 (d, *J* = 2.5 Hz, 2H), 4.51 (q, *J* = 7.2 Hz, 1H), 1.79 (d, *J* = 7.2 Hz, 3H). ^13^C NMR (CDCl_3_, 101 MHz): *δ* 157.90 (s), 144.09 (s), 143.89 (s), 142.71 (s), 45.07 (s), 19.05 (s). HR-ESI-MS (M^+^) *m*/*z* calc. for [**3a** + H^+^], 187.0978, found, 187.0979.

#### 2,2′-(1-(6-Fluoropyridin-2-yl)ethane-1,1-diyl)dipyrazine, (PZ2PYMeF, **3b**)

To a 2-neck round bottom flask equipped with reflux condenser was added compound **3a** (4.62 g, 24.8 mmol), which was thoroughly dried and degassed before the flask was charged with 50 mL anhydrous 1,4-dioxane. The mixture was cooled to 0 °C and a 1.8 M solution of LDA (14.5 mL, 26.1 mmol, 1.05 equivalents) was added by syringe. The mixture was allowed to react for 15 min before it was warmed to room temperature, after which 2,6-difluoropyridine (0.649 g, 0.512 mL, 5.64 mmol, 4.4 equivalents) was added and the mixture was refluxed at 115 °C for 2 days. Upon cooling, residual LDA was quenched with an excess of water, and the product was extracted into diethyl ether. The organic phased was dried over anhydrous sodium sulfate, taken to dryness on a rotary evaporator, and finally purified by silica gel chromatography eluting with 99 : 1 dichloromethane : triethylamine. The purified product was washed with water and dried to yield an off-white solid, 1.44 g (91%). No trace of the di-substituted product was observed. ^1^H NMR (CDCl_3_, 400 MHz): *δ* 8.51 (dd, *J* = 2.5, 1.6 Hz, 2H), 8.48 (d, *J* = 1.6 Hz, 2H), 8.45 (d, *J* = 2.5 Hz, 2H), 7.76 (q, *J* = 8.0 Hz, 1H), 7.04 (dd, *J* = 7.6, 2.5 Hz, 1H), 6.84 (dd, *J* = 8.2, 3.1 Hz, 1H), 2.31 (s, 3H). ^13^C NMR (CDCl_3_, 101 MHz): *δ* 159.91 (s), 145.35 (s), 143.34 (s), 142.51 (s), 141.68 (s), 141.60 (s), 120.28 (s), 108.33 (s), 107.96 (s), 57.14 (s), 26.28 (s). Elem. Anal. calc. for C_15_H_12_FN_5_: C, 64.05; H, 4.30; N, 24.90. Found: C, 63.88; H, 4.39; N, 24.68. HR-ESI-MS (M^+^) *m*/*z* calc. for [**3b** + H^+^], 282.1150, found, 282.1151.

#### 2,2′-(1-(6-(1,1-Di(pyridin-2-yl)ethyl)pyridin-2-yl)ethane-1,1-diyl)dipyrazine, (PY3PZ2Me_2_, **3**)

To a 2-neck round bottom flask equipped with reflux condenser was added 2,2′-(ethane-1,1-diyl)dipyridine (1.31 g, 7.11 mmol) in 50 mL anhydrous 1,2-dimethoxyethane, which was cooled to 0 °C before addition of a 1.8 M LDA solution (4.15 mL, 7.47 mmol, 1.05 equivalents). After letting the reaction stir for 15 min, compound **3b** (1.00 g, 3.56 mmol) was added prior to refluxing the mixture at 105 °C for 1.5 days. It was allowed to cool to room temperature before residual LDA was quenched with a copious amount of water. Organics were extracted with diethyl ether, dried over anhydrous sodium sulfate, and the solvent was removed by rotary evaporation. The product was purified by alumina gel chromatography eluting with 1 : 1 ethyl acetate : hexanes with the third spot off the column being pure ligand, an off-white solid, 0.998 g (63%). ^1^H NMR (CDCl_3_, 400 MHz): *δ* 8.52 (ddt, *J* = 4.3, 2.4, 1.2 Hz, 2H), 8.42 (dd, *J* = 2.5, 1.5 Hz, 2H), 8.34 (d, *J* = 2.5 Hz, 2H), 8.20 (d, *J* = 1.5 Hz, 2H), 7.63 (t, *J* = 7.9 Hz, 1H), 7.46 (td, *J* = 7.8, 1.9 Hz, 2H), 7.18 (dd, *J* = 7.9, 0.8 Hz, 1H), 7.12 (dd, *J* = 8.0, 0.8 Hz, 1H), 7.08 (ddd, *J* = 7.6, 4.9, 1.1 Hz, 2H), 6.85 (dt, *J* = 8.0, 1.1 Hz, 2H), 2.20 (s, 3H), 2.17 (s, 3H). ^13^C NMR (CDCl_3_, 101 MHz): *δ* 165.61 (s), 165.27 (s), 161.78 (s), 160.79 (s), 148.56 (s), 145.99 (s), 142.71 (s), 141.70 (s), 137.16 (s), 135.68 (s), 123.49 (s), 121.10 (s), 120.90 (s), 119.27 (s), 60.03 (s), 57.39 (s), 26.86 (s), 25.84 (s). Elem. Anal. calc. for C_27_H_23_N_7_: C, 72.79; H, 5.20; N, 22.01. Found: C, 72.38; H, 5.33; N, 21.88. HR-ESI-MS (M^+^) *m*/*z* calc. for [**3** + H^+^], 446.2088, found, 446.2088.

### General procedure for synthesis of metal complexes

To a 20 mL scintillation vial was added 0.1 g of the desired pentadentate ligand and 1 equivalent of Zn(OTf)_2_ or Co(CH_3_CN)_2_(OTf)_2_ (anhydrous metal precursors were stored in a glovebox). Next, 5 mL of a 9 : 1 acetone : H_2_O mixture was added and the reaction mixture was stirred overnight at room temperature under an N_2_ atmosphere. Details of the recrystallization procedures and yields are provided below for each complex.

#### [(ax-PY4PZMe_2_)Co(OH_2_)](OTf)_2_, (**1-Co**)

The reaction mixture was taken to dryness under vacuum and the solid was re-dissolved in a minimal amount of 19 : 1 acetone : H_2_O. The recrystallization vessel was degassed with N_2_ before it was sealed. Golden crystals suitable for X-ray crystallography were grown by slow diffusion of diethyl ether into the concentrated solution to yield 0.147 g (80%). Elem. Anal. calc. for C_30_H_26_CoF_6_N_6_O_7_S_2_: C, 43.96; H, 3.20; N, 10.25. Found: C, 44.21; H, 2.93; N, 10.26. HR-ESI-MS (M^+^) *m*/*z* calc. for [(ax-PY4PZMe_2_)Co^2+^], 251.5692, found, 251.5692; *m*/*z* calc. for [(ax-PY4PZMe_2_)Co(OTf)^+^], 652.0909, Found, 652.0908.

#### [(ax-PY4PZMe_2_)Zn(OH_2_)](OTf)_2_, (**1-Zn**)

The reaction vial was left open to air for slow evaporation of the solvent to yield X-ray quality colorless crystals, 0.154 g (83%). ^1^H NMR (acetone-*d*_6_, 400 MHz): *δ* 9.49 (s, 2H), 9.45 (dd, *J* = 5.2, 1.6 Hz, 4H), 8.20 (dt, *J* = 8.4, 1.0 Hz, 4H), 8.13 (td, *J* = 8.0, 1.8 Hz, 4H), 7.72 (ddd, *J* = 7.5, 5.3, 1.2 Hz, 4H), 2.96 (s, 6H). ^13^C NMR (acetone-*d*_6_, minimal amt. of D_2_O for solubility), 101 MHz: 158.06 (s), 151.90 (s), 150.00 (s), 144.30 (s), 141.70 (s), 125.47 (s), 124.08 (s), 48.66 (s), 23.28 (s). Elem. Anal. calc. for C_30_H_26_F_6_N_6_O_7_S_2_Zn: C, 43.62; H, 3.17; N, 10.17. Found: C, 44.01; H, 2.87; N, 10.16. HR-ESI-MS (M^+^) *m*/*z* calc. for [(eq-PY4PZMe_2_)Zn(OTf)^+^], 657.0869, found, 657.0881.

#### [(eq-PY4PZMe_2_)Co(OH_2_)](OTf)_2_, (**2-Co**)

The reaction mixture was taken to dryness under vacuum and the solid was re-dissolved in a minimal amount of 19 : 1 acetone : H_2_O. The recrystallization vessel was degassed with N_2_ before it was sealed. Golden crystals suitable for X-ray crystallography were grown by slow diffusion of diethyl ether into the concentrated solution to yield 0.120 g (65%). Elem. Anal. calc. for C_30_H_26_CoF_6_N_6_O_7_S_2_: C, 43.96; H, 3.20; N, 10.25. Found: C, 43.43; H, 3.12; N, 10.17. HR-ESI-MS (M^+^) *m*/*z* calc. for [(eq-PY4PZMe_2_)Co^2+^], 251.5692, found, 251.5692; *m*/*z* calc. for [(eq-PY4PZMe_2_)Co(OTf)^+^], 652.0909, found, 652.0906.

#### [(eq-PY4PZMe_2_)Zn(OH_2_)](OTf)_2_, (**2-Zn**)

The reaction vial was left open to air for slow evaporation of the solvent to yield X-ray quality colorless crystals, 0.111 g (60%). ^1^H NMR (acetone-*d*_6_, 400 MHz): *δ* 9.53 (ddd, *J* = 5.1, 2.4, 1.5 Hz, 2H), 9.48 (dd, *J* = 1.8, 0.8 Hz, 1H), 9.47 (d, *J* = 1.4 Hz, 1H), 9.44 (dd, *J* = 2.8, 1.3 Hz, 1H), 9.01 (d, *J* = 2.8 Hz, 1H), 8.35 (dd, *J* = 5.9, 1.1 Hz, 1H), 8.33 (dd, *J* = 6.4, 1.2 Hz, 1H), 8.31–8.21 (m, 4H), 8.17 (qd, *J* = 8.4, 1.8 Hz, 3H), 7.82–7.72 (m, 3H), 3.04 (s, 3H), 2.92 (s, 3H). ^13^C NMR (acetone-*d*_6_, 101 MHz): *δ* 159.00 (s), 158.98 (s), 158.89 (s), 158.44 (s), 158.35 (s), 153.54 (s), 150.37 (s), 150.09 (s), 147.03 (s), 146.23 (s), 143.14 (s), 142.88 (s), 142.00 (s), 141.95 (s), 125.56 (s), 125.47 (s), 124.49 (s), 124.33 (s), 124.29 (s), 123.55 (s), 123.49 (s), 50.32 (s), 49.34 (s), 24.65 (s), 23.74 (s). Elem. Anal. calc. for C_30_H_26_F_6_N_6_O_7_S_2_Zn: C, 43.62; H, 3.17; N, 10.17. Found: C, 43.47; H, 3.22; N, 10.06. HR-ESI-MS (M^+^) *m*/*z* calc. for [(eq-PY4PZMe_2_)Zn^2+^], 254.0672, found, 254.0674; *m*/*z* calc. for [(eq-PY4PZMe_2_)Zn(OTf)^+^], 657.0869, found, 657.0880.

#### [(PY3PZ2Me_2_)Co(OH_2_)](OTf)_2_, (**3-Co**)

The reaction mixture was taken to dryness under vacuum and the solid was re-dissolved in a minimal amount of 19 : 1 acetone : H_2_O. The recrystallization vessel was degassed with N_2_ before it was sealed. Golden crystals suitable for X-ray crystallography were grown by slow diffusion of diethyl ether into the concentrated solution to yield 0.140 g (76%). Elem. Anal. calc. for C_29_H_25_CoF_6_N_7_O_7_S_2_: C, 42.45; H, 3.07; N, 11.95. Found: C, 42.08; H, 3.02; N, 11.60. HR-ESI-MS (M^+^) *m*/*z* calc. for [(PY3PZ2Me_2_)Co^2+^], 252.0668, found, 252.0670; *m*/*z* calc. for [(PY3PZ2Me_2_)Co(OTf)^+^], 653.0867, found, 653.0862.

#### [(PY3PZ2Me_2_)Zn(OH_2_)](OTf)_2_, (**3-Zn**)

The reaction vial was left open to air for slow evaporation of the solvent to yield X-ray quality colorless crystals, 0.134 (72%). ^1^H NMR (acetone-*d*_6_, 400 MHz): *δ* 9.51 (d, *J* = 1.7 Hz, 1H), 9.50 (d, *J* = 1.5 Hz, 2H), 9.47 (dd, *J* = 2.9, 1.3 Hz, 2H), 9.02 (d, *J* = 2.8 Hz, 2H), 8.38 (dd, *J* = 11.2, 0.8 Hz, 1H), 8.36 (dd, *J* = 12.0, 1.2 Hz, 2H), 8.30 (d, *J* = 8.1 Hz, 1H), 8.28–8.22 (m, 2H), 8.17 (td, *J* = 7.9, 1.8 Hz, 2H), 7.77 (ddd, *J* = 7.5, 5.2, 1.2 Hz, 2H), 3.17 (s, 3H), 2.91 (s, 3H). ^13^C NMR (acetone-*d*_6_, 101 MHz): *δ* 159.07 (s), 158.77 (s), 157.76 (s), 152.84 (s), 150.09 (s), 147.23 (s), 146.27 (s), 143.18 (s), 143.15 (s), 142.09 (s), 125.63 (s), 124.34 (s), 123.84 (s), 123.69 (s), 50.29 (s), 48.28 (s), 24.64 (s), 22.84 (s). Elem. Anal. calc. for C_29_H_25_F_6_N_7_O_7_S_2_Zn: C, 42.11; H, 3.05; N, 11.85. Found: C, 42.07; H, 3.22; N, 11.65. HR-ESI-MS (M^+^) *m*/*z* calc. for [(PY3PZ2Me_2_)Zn(OTf)^+^], 658.0821, found, 658.0836.

## Results and discussion

### Design, synthesis, and structural chemistry

With the goal of developing effective molecular catalysts for hydrogen evolution directly from water, we were inspired by the tightly regulated electronic communication found in mononuclear metalloenzymes that enable multielectron chemistry through the synergistic interplay of a single metal center with pendant redox-active cofactors.^37–43^ Seeking to transfer this design concept to synthetic systems, we reasoned that introducing redox non-innocent functionalities into specific locations within structurally well-defined ligand motifs would allow us to evaluate the relationship between geometric placement and effectiveness of electron reservoirs in catalysis.

**Scheme 1:**
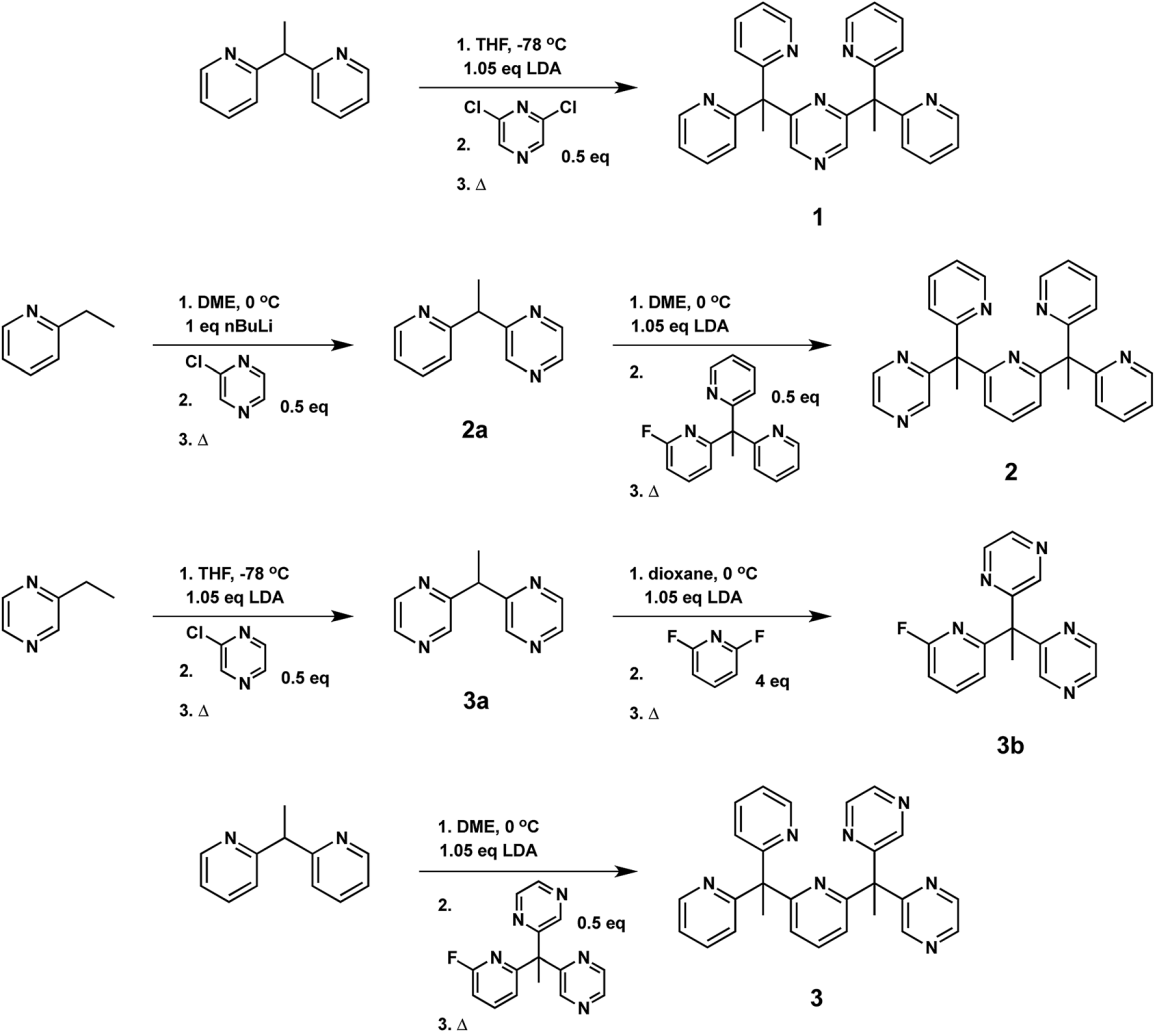
Synthesis of pentadentate PY5Me_2_-type ligands (**1–3**) containing pyrazine(s) at key positions in the framework.

Specifically, we prepared the pentadentate ligands, ax-PY4PZMe_2_ (**1**), eq-PY4PZMe_2_ (**2**), and PY3PZ2Me_2_ (**3**), as well as their ligand precursors, by standard lithiation reactions followed by nucleophilic aromatic substitution with the desired halogen-substituted heterocycle (Scheme 1). The targeted introduction of redox non-innocent pyrazines in axial *versus* equatorial positions allows for testing the effects of redox-active reservoir location on catalytic hydrogen evolution activity within a structurally homologous motif. Notably, lithiations were performed with the sterically-hindered base lithium diisopropylamide when deprotonating pyrazine-based reagents, as the more nucleophilic reagent *n*-butyl lithium resulted in alkylation of the pyrazine ring.^71^ Moreover, successful coupling of ligand fragments is governed by appropriate matching of nucleophilic and electrophilic partners, and thoughtful attention to each stepwise synthetic sequence is required. For example, several attempts were made to react **3a** with PY3MeF to form **3**, including an extended reflux in 1,4-dioxane, but no synthetically useful conversion was observed. Thus, **3a** was reacted first with 2,6-difluoropyridine to generate **3b**, which is more electrophilic than its pyridine analogue, PY3MeF. Subsequent lithiation of 2,2′-(ethane-1,1-diyl)dipyridine, a better nucleophile than the pyrazine analogue **3a**, was reacted with **3b** to produce **3** in 63% yield.

**Fig. 2:**
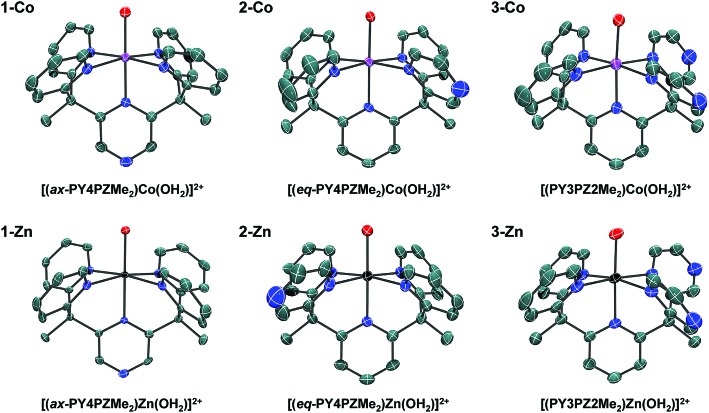
Crystal structures of cations in the following salts: **1-Co**, [(ax-PY4PZMe_2_)Co(OH_2_)](OTf)_2_; **2-Co**, [(eq-PY4PZMe_2_)Co(OH_2_)](OTf)_2_; **3-Co**, [(PY3PZ2Me_2_)Co(OH_2_)](OTf)_2_; **1-Zn**, [(ax-PY4PZMe_2_)Zn(OH_2_)](OTf)_2_; **2-Zn**, [(eq-PY4PZMe_2_)Zn(OH_2_)](OTf)_2_; **3-Zn**, [(PY3PZ2Me_2_)Zn(OH_2_)](OTf)_2_. Thermal ellipsoids are drawn at the 70% probability level. Hydrogen atoms have been omitted for clarity.

Facile metalation of the pentadentate ligands was achieved at room temperature in 9 : 1 acetone : water mixtures using Co(CH_3_CN)_2_(OTf)_2_ or Zn(OTf)_2_ metal precursors. Crystals of the resulting cobalt complexes, suitable for X-ray diffraction, were grown by ether diffusion into concentrated acetone solutions. X-ray quality crystals of the zinc complexes were grown by slow evaporation of the reaction mixture. Solid-state structures of each pair of metal complexes, Co and Zn, supported by ligands **1**, **2**, and **3** were obtained by single crystal X-ray diffraction (Fig. 2, Table 1).

**Table 1:** Selected bond distances of related pentadentate Co and Zn complexes

Complex^*a*^	Coordination environment^*b*^ (M = Co, Zn)			
	M–N_ax_	M–O	avg M–N_eq_	Tilt of axial heterocycle (°)
[(PY5Me_2_)**Co**(OH_2_)]^2+^ (ref. 10*a*)	2.103	2.055	2.139	4.60
[(PY5Me_2_)**Zn**(OH_2_)]^2+^ (ref. 10*a*)	2.116	2.056	2.162	11.26
[(CF_3_PY5Me_2_)**Co**(OH_2_)]^2+^ (ref. 10*a*)	2.132	2.050	2.129	9.88
**1-Co**, [(ax-PY4PZMe_2_)**Co**(OH_2_)]^2+^	2.1050(13)	2.0342(12)	2.1415(13)	7.58
**1-Zn**, [(ax-PY4PZMe_2_)**Zn**(OH_2_)]^2+^	2.147(3)	2.039(3)	2.160(4)	7.15
**2-Co**, [(eq-PY4PZMe_2_)**Co**(OH_2_)]^2+^	2.099(2)	2.0316(19)	2.127(2)	4.78
**2-Zn**, [(eq-PY4PZMe_2_)**Zn**(OH_2_)]^2+^	2.1368(19)	2.0608(17)	2.150(2)	2.24
**3-Co**, [(PY3PZ2Me_2_)**Co**(OH_2_)]^2+^	2.094(3)	2.016(3)	2.113(3)	5.67
**3-Zn**, [(PY3PZ2Me_2_)**Zn**(OH_2_)]^2+^	2.141(2)	2.0474(18)	2.157(2)	4.75

^*a*^All complexes recrystallized as the triflate salt.

^*b*^Bond distances are reported in angstroms (Å).

Slightly distorted octahedral geometries are adopted in each of the six metal complexes in this systematic series. As expected, the pentadentate ligands leave one open coordination site for exogenous ligands such water or acetonitrile. The equatorial pyrazine in the crystal structure of **2-Co** is disordered over two positions, with the dominant position accounting for 61% of the refined structure solution. In contrast, disorder is not observed in structures of the Co(ii) and Zn(ii) complex cations, **3-Co** and **3-Zn**, supported by the *C*_s_-symmetric ligand, PY3PZ2Me_2_ (**3**). Disordered outer-sphere solvent and/or triflate ions are observed in several of the structures. Additional details can be found in the CIF files.

In Table 1, Co–O and averaged equatorial Co–N bond distances of related complexes reveal a trend consistent with increased metal-to-ligand backbonding in pyrazine containing species. For example, the average Co-N_eq_ distances are 2.1415(13), 2.127(2), and 2.113(3) Å in **1-Co**, **2-Co**, and **3-Co**, respectively. Likewise, a Co–O distance of 2.055 Å was reported for the parent complex, [(PY5Me_2_)Co(OH_2_)]^2+^, with shorter distances, 2.0342(12) and 2.0316(19) Å, observed in [(PY4PZMe_2_)Co(OH_2_)]^2+^ isomers, and a progressively shorter distance, 2.016(3) Å, found in **3-Co**. Observed bond distances in this series of cobalt complexes are similar to those of known high-spin Co(ii) (*S* = 3/2) systems, such as [(PY5Me_2_)Co(OH_2_)]^2+^.^10^

### Electrochemistry in acetonitrile solution with glassy carbon electrodes

Non-aqueous cyclic voltammetry experiments were conducted in acetonitrile/0.1 M Bu_4_NPF_6_ solutions for each ligand and their corresponding metal complexes at concentrations of 1 mM (Fig. S1–S7†). Data from these experiments are summarized in Table 2. Notably, non-aqueous cyclic voltammograms (CVs) in CH_3_CN produced the same waves when using Co-aquo metal complexes **1-Co**, **2-Co**, and **3-Co** or complexes formed by metalating each ligand in acetonitrile (Fig. S5–S7†), consistent with facile solvent exchange of the axial aquo ligand with CH_3_CN. CVs in CH_3_CN are internally referenced to the ferrocenium/ferrocene (Fc^+^/Fc) couple.

**Table 2:** Cyclic voltammetry results (*V vs.* Fc^+^/Fc) for Co and Zn complexes in acetonitrile/0.1 M Bu_4_NPF_6_

Complex	*E*_1/2_ (Co^III/II^)	*E*_p1,c_	*E*_p2,c_	*E*_p3,c_
**1-Co**, [(ax-PY4PZMe_2_)Co(OH_2_)]^2+^	0.32	–1.22	–1.40	—
**1-Zn**, [(ax-PY4PZMe_2_)Zn(OH_2_)]^2+^	—	—	–1.69	–1.90
**2-Co**, [(eq-PY4PZMe_2_)Co(OH_2_)]^2+^	0.27	–1.30	–1.42	–2.04
**2-Zn**, [(eq-PY4PZMe_2_)Zn(OH_2_)]^2+^	—	—	–1.70	–1.83
**3-Co**, [(PY3PZ2Me_2_)Co(OH_2_)]^2+^	0.35	–1.18	–1.25	–1.95
**3-Zn**, [(PY3PZ2Me_2_)Zn(OH_2_)]^2+^	—	—	–1.45	–1.75
[(PY5Me_2_)Co(CH_3_CN)]^2+^ (ref. 10*a*)	0.24	–1.47	–2.36	—
[(PY5Me_2_)Zn(OH_2_)]^2+^ ^*a*^	—	—	—	—

^*a*^Electrochemistry in CH_3_CN was performed to confirm its electrochemical silence as previously reported in CH_2_Cl_2_ solution (ref. 10*a*).

Cyclic voltammograms of each ligand are electrochemically-silent up to potentials more negative than –2.25 V *vs.* Fc^+^/Fc (Fig. S1†). Upon metalation with Zn(ii), redox events that are irreversible on the CV time-scale are observed for each complex, consistent with ligand-based reductions (Fig. S2–S4†). Under the same conditions, the previously studied pentapyridine analogue, [(PY5Me_2_)Zn(OH_2_)](OTf)_2_, displayed no redox activity over the same potential range,^10*a*^ indicating that pyrazine moieties in **1-Zn**, **2-Zn**, and **3-Zn** are reduced. As expected, reductions for axial (**1-Zn**) and equatorial (**2-Zn**) isomers of [(PY4PZMe_2_)Zn(OH_2_)](OTf)_2_ are similar with the first reduction occurring at –1.7 V *vs.* Fc^+^/Fc. The first reduction for **3-Zn**, [(PY3PZ2Me_2_)Zn(OH_2_)](OTf)_2_, is shifted positively by 250 mV (–1.45 V) with a second reduction event located at –1.75 V *vs.* Fc^+^/Fc.

The cyclic voltammograms of the Co complexes have additional features that we ascribe to metal-based redox events. Catalyst isomers **1-Co** and **2-Co** exhibit reversible waves at 0.32 and 0.27 V (Co(iii/ii)), and irreversible reductions at –1.22 and –1.30 V *vs.* Fc^+^/Fc, respectively (Fig. S5 and S6†). Consistent with increased metal-to-ligand backbonding, the oxidative Co(iii/ii) couple of **3-Co** is shifted anodically to 0.35 V and its first reduction is shifted to –1.18 V *vs.* Fc^+^/Fc (Fig. S7†), which is positive relative to the first reductions of **1-Co** and **2-Co**. We tentatively assign the first reductions to metal-centered Co(ii/i) events on the basis of theoretical studies (see below); however, experimentally, the waves are overlapping and difficult to distinguish. Additional ligand-based reductions are seen at more negative potentials. For **1-Co**, a second peak at –1.40 V is observed with no readily distinguishable waves at more negative potentials. Second reductions are also apparent at –1.42 V and –1.25 V for **2-Co** and **3-Co**, respectively. To verify that these waves are not due to an equilibrium of species in solution, but are two closely-spaced one-electron processes, square wave voltammetry was conducted to determine the electron content of the observed waves. These results (Fig. S5B–S7B†) show a 1 : 2 ratio of integrated peaks for the well-defined Co(iii/ii) waves relative to the more negative closely-spaced redox features of each cobalt complex.

Interestingly, a third reduction is observed for each of **2-Co** (–2.04 V) and **3-Co** (–1.95 V), which are both cathodic relative to reductions of their corresponding Zn complexes, **2-Zn** and **3-Zn**, and likely a consequence of forming anionic species (eqn (1)). Density functional theory calculations suggest that the third reduction is mainly ligand-centered (see ESI†).1
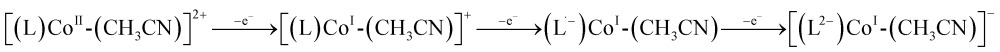



### Catalytic proton reduction in acetonitrile with chloroacetic acid

**Fig. 3:**
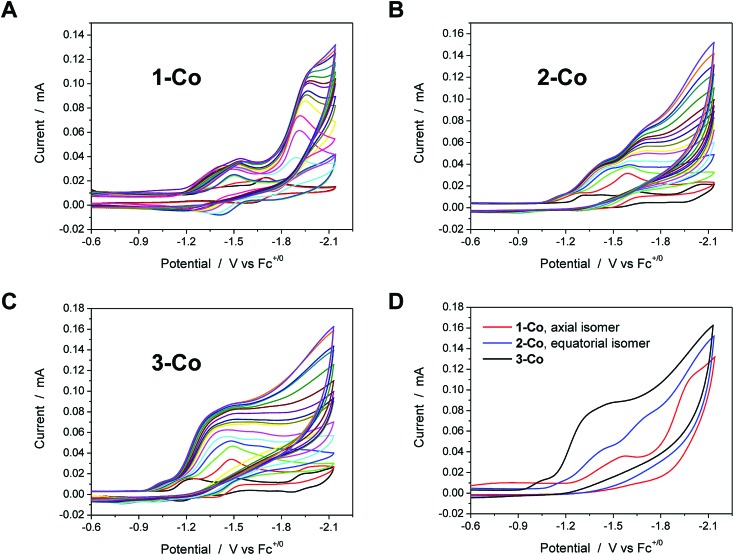
Cyclic voltammetry of catalysts (1 mM) in acetonitrile with various concentrations of chloroacetic acid (up to 20 equivalents, 20 mM). A, B, and C. Catalysts **1-Co**, **2-Co**, and **3-Co**, respectively. The CV in black in these three graphs is the indicated catalyst in the absence of acid. D. Comparison of CVs for each catalyst, **1-Co** (red), **2-Co** (blue), and **3-Co** (black), with 20 eq. of chloroacetic acid. Scan rate = 100 mV s^–1^; 3 mm dia. glassy carbon.

Electrocatalysis with cobalt complexes under homogeneous, diffusion-limited conditions was conducted initially by cyclic voltammetry with a weak acid proton source in acetonitrile solution. Significant current enhancements, relative to the bare glassy carbon electrode, are afforded with various amounts of chloroacetic acid (*E*0MeCN = –1.05 V *vs.* Fc^+^/Fc)^72^ in each catalyst solution. The current responses following addition of up to 20 equivalents of acid are shown for **1-Co**, **2-Co**, and **3-Co** in Fig. 3.

Each of the cobalt complexes is a competent electrocatalyst as verified by direct hydrogen measurements. Notably, catalysis with **1-Co** occurs at more negative potentials than its initial reductions with an onset at *ca.* –1.7 V *vs.* Fc^+^/Fc. The current profiles for catalysts with an equatorial pyrazine group, however, display catalytic current enhancements that overlap with the initial reductions and results in considerably lower overpotentials for hydrogen evolution catalysis. For **2-Co**, the first reduction does not vary significantly with acid concentration, but is followed closely by a catalytic second reduction with an onset of *ca.* –1.5 V. A small pre-feature, or first reduction event, is also observed with **3-Co**, followed by a steep rise in current at *ca.* –1.15 V, indicative of catalysis. Fig. 3D provides a comparison of CVs for each catalyst in the presence of 20 equivalents of chloroacetic acid.

Electrocatalytic hydrogen production was measured directly by gas chromatography of samples obtained from the headspace of electrochemical cells. The amount of H_2_ generated was quantified using a calibration curve based on an internal standard (CH_4_) of known volume that was injected into the headspace of degassed, airtight electrochemical cells prior to electrolysis (Fig. S8†). A glassy carbon rod was employed as the working electrode during controlled potential electrolyses (Fig. S9†) at a fixed potential of –1.5 V *vs.* Fc^+^/Fc for the hydrogen measurements. Zinc complexes do not show proton reduction activity at this potential. As expected from cyclic voltammograms, the activity of **1-Co** under these conditions with chloroacetic acid is relatively low, but surprisingly similar to that of **3-Co**. The best catalyst from the Co series is **2-Co** by a factor of ∼2. Faradaic efficiencies of all catalysts were >90% for evolved hydrogen, verifying that virtually all of the total charged passed was used efficiently to reduce protons.

### Aqueous electrochemical studies

**Fig. 4:**
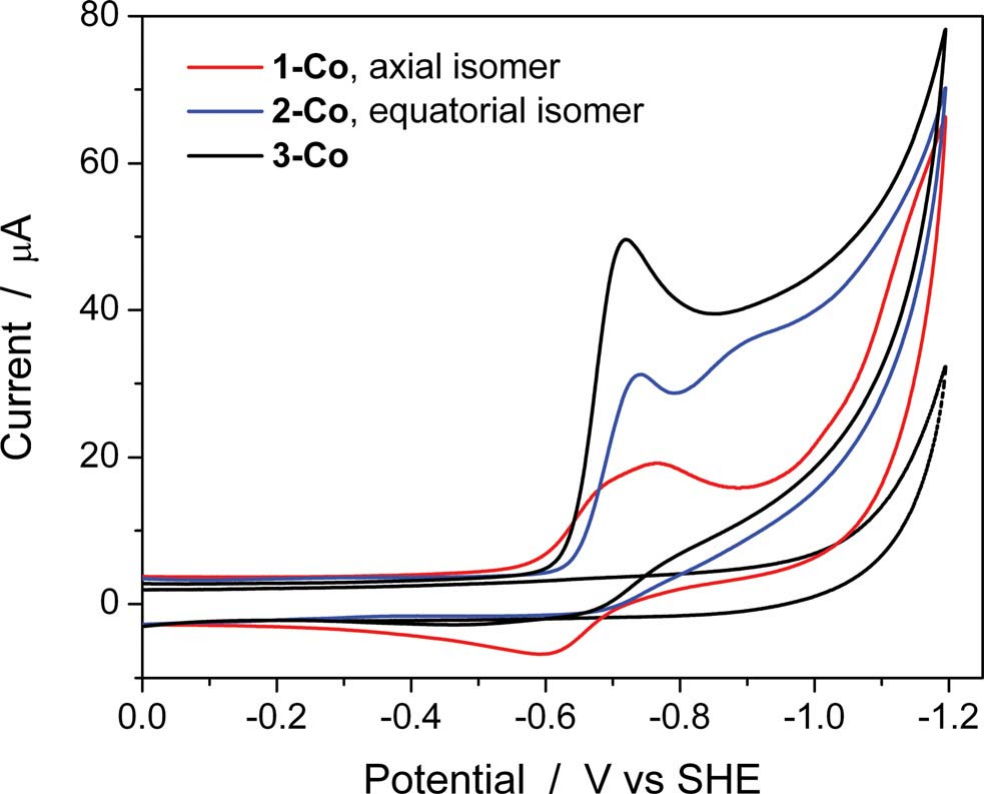
Aqueous cyclic voltammetry of catalysts **1-Co** (red), **2-Co** (blue), and **3-Co** (black) in 1 M pH 7 KPBS at glassy carbon electrode. Catalyst concentration is 1 mM and scan rate = 100 mV s^–1^. Background is shown as a dotted gray line.

Cyclic voltammetry studies were also performed in aqueous solution at physiological pH (1 M pH 7 potassium phosphate buffer, KPBS). Notably, the Zn(ii) complexes bearing the redox non-innocent pyrazine donors do show electrochemical activity in the potential window afforded by the glassy carbon electrode, but they are not catalysts for the hydrogen production reaction. This important observation highlights the fact that the redox-active cobalt ions along with the redox-active ancillary ligands **1–3** are required for catalysis. Fig. 4 displays the CVs of Co complexes **1-Co**, **2-Co**, and **3-Co** at 1 mM concentrations at pH 7 under the same conditions. Oxidative waves are also observed with *E*_1/2_ values of 0.34 V, 0.35 V, and 0.42 V *vs.* SHE, respectively. We assign these waves to Co(iii/ii) couples, consistent with structurally-related polypyridine Co complexes.^10,14,18a^

At reducing potentials, a clear demarcation in behavior is observed between axial isomer **1-Co** and its equatorial isomer **2-Co**, as well as **3-Co** which bears two pyrazine moieties in equatorial positions along the ligand framework. Quasi-reversible reductions of **1-Co** at *E*_p,c_ = –0.68 and –0.76 V are followed by a ∼250 mV separation before a catalytic wave appears with an onset at *ca.* –1.0 V *vs.* SHE. In contrast, irreversible reductions of equatorial pyrazine complexes **2-Co** and **3-Co** are observed that coincide with catalytic hydrogen production. Interestingly, the lag between initial reductions and catalytic onset is not observed with **2-Co** and **3-Co**.

**Fig. 5:**
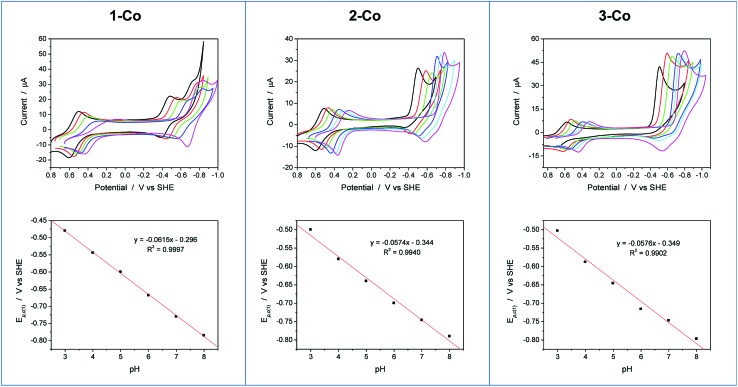
Cyclic voltammograms as a function of pH and plots of the first reductive peak potential (*E*_p,c(1)_) *vs.* pH for **1-Co**, **2-Co**, and **3-Co**. *Conditions*: 0.9 mM catalyst, 0.03 M buffer, 0.1 M KNO_3_, 100 mV s^–1^ scan rate, glassy carbon electrode (3 mm dia). Note: CV at pH 7 is omitted for clarity in series of CVs for **1-Co**. Legend: pH 3 (black), pH 4 (red), pH 5 (green), pH 6 (blue), pH 7 (cyan), pH 8 (magenta).

Cyclic voltammetry experiments as a function of pH were conducted to supplement our understanding of these redox events. Cyclic voltammograms over a pH range of 3 to 8 are plotted for each catalyst as shown in Fig. 5. At positive potentials, a reversible oxidation is observed for each catalyst that is consistent with a Co(iii)–OH/Co(ii)–OH_2_ couple. Slopes of 54, 52, and 54 mV per pH unit are obtained from the *E*_1/2_ *vs.* pH plots shown in Fig. S10† and are near the expected value of 59 mV per pH unit for a metal-centered 1H^+^/1e^–^ process. At negative potentials, two well-separated waves are observed at low pH values for **1-Co** with a reductive peak separation of ∼240 mV at pH 3. The first reduction is quasi-reversible and has a pH dependence of 62 mV per pH unit, while the second reduction is irreversible and has a pH dependence of ∼22 mV per pH unit (Fig. S11†). As the pH is increased, the waves begin to coalesce into closely-spaced, overlapping redox features. The pH-dependence of the second reduction is scattered from linearity, but is qualitatively similar to previously reported water reduction catalysts^10,13^ and suggests a 1H^+^/2e^–^ process. This result may indicate that a third reduction is necessary for initiating reactivity from **1-Co**, consistent with the higher overpotential required for catalysis following the first two redox events. In contrast, a single irreversible, catalytic reduction is observed across the pH range for **2-Co** and **3-Co**, displaying pH dependences of 57 and 58 mV per pH unit, respectively, consistent with a 1H^+^/1e^–^ process.

The pH-dependent cyclic voltammetry of **1-Zn** (Fig. S12†) indicates that ligand-based pyrazine reduction is accompanied by protonation, likely at its distal nitrogen. Skewed waves are observed with a pH dependence of 60 mV per pH unit with reductive peak potentials that are similar to the potentials of the second reduction observed in **1-Co**. On this basis, together with the nonaqueous electrochemical results described earlier, the closely-spaced reductions observed in **1-Co** are consistent with two 1-electron processes involving a metal-centered Co(ii/i) reduction followed by a ligand-based pyrazine reduction.

Electrocatalytic activity and stability of the cobalt complexes during the hydrogen evolution reaction in neutral, phosphate-buffered water was studied by controlled potential electrolysis. A mercury pool working electrode was used to minimize the background reaction, and two different potentials were analyzed. At a fixed potential of –1.0 V *vs.* SHE, **2-Co** and **3-Co** perform with nearly identical activities while far surpassing the capability of axial isomer **1-Co** (Fig. S13†). The same behavior is observed at –1.2 V *vs.* SHE, but with **3-Co** passing a charge of nearly 350 coulombs after 12 h and **2-Co** accounting for nearly 300 C over the same timeframe. The Faradaic efficiency for evolved hydrogen was monitored by gas chromatography for catalysts **1-Co**, **2-Co**, and **3-Co** at various time points during electrolysis and found to be *ca.* 100%, which confirms that all of the electrons transferred from the working electrode were used to generate hydrogen (Fig. S14†).

### Rotating disk electrode voltammetry studies

To further probe the electrochemical signatures of this series of catalysts, we employed rotating disk electrode voltammetry (RDEV). The apparent *n*_app_ value for each catalyst was determined to directly compare their potential-dependent catalytic activity. The value of *n*_app_ is a measure of the rate of electron delivery from the electrode surface to the catalyst before it diffuses away, where the diffusion coefficient is accounted for by normalizing, internally, the electrocatalytic current density (*j*_c_) to the plateau current density of the Co(iii)–OH/Co(ii)–OH_2_ couple (*j*_p_).

**Fig. 6:**
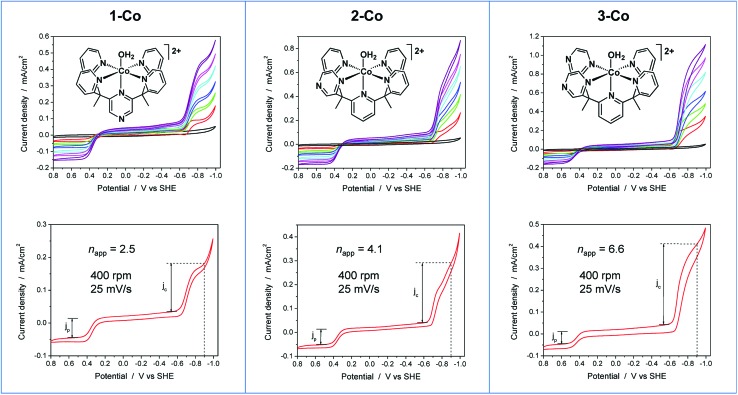
RDEV of the series of cobalt catalysts. *Panels*: axial isomer **1-Co**, [(ax-PY4PZMe_2_)Co(OH_2_)]^2+^; equatorial isomer **2-Co**, [(eq-PY4PZMe_2_)Co(OH_2_)]^2+^; and catalyst **3-Co**, [(PY3PZ2Me_2_)Co(OH_2_)]^2+^. *Top*: rotation rates of 100–3600 rpm; *bottom*: apparent *n*_app_ based on 1e^–^ Co(iii/ii) couple at 400 rpm. *Conditions*: 0.3 mM catalyst, 0.1 M pH 7 KPBS, 0.1 M KNO_3_, 25 mV s^–1^ scan rate, glassy carbon electrode (3 mm dia).

Results are shown in Fig. 6 for the catalyst series, **1-Co**, **2-Co**, and **3-Co**. The RDEV conditions and parameters employed here are analogous to those of previous studies of hydrogen evolving catalysts conducted in 0.1 M pH 7 phosphate buffer,^10c,13^ but 0.1 M NaClO_4_ was replaced with 0.1 M KNO_3_ owing to poor catalyst solubility in the former electrolyte. The top of each panel displays the steady-state voltammograms of current density *vs.* potential at a scan rate of 25 mV s^–1^ and rotation rates from 100 to 3600 rpm. The bottom of each panel shows the data obtained at 400 rpm used to calculate the potential-dependent *n*_app_ for catalysis at –0.9 V *vs.* SHE.

**Fig. 7:**
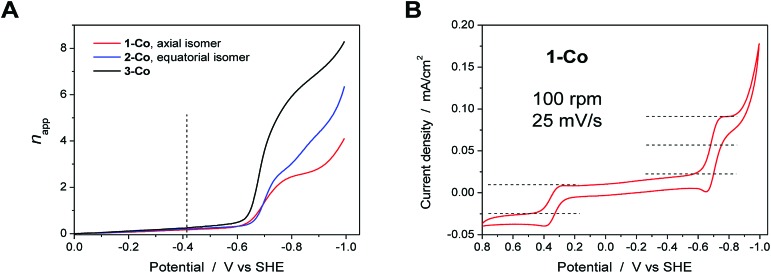
A. Comparison of *n*_app_ *vs.* applied potential for catalysts **1-Co** (red), **2-Co** (blue), and **3-Co** (black) based on steady-state voltammograms in Fig. 6 (bottom). The vertical dashed line denotes the thermodynamic potential for water reduction at pH 7. B. Steady-state voltammogram of catalyst **1-Co** at 100 rpm, 25 mV s^–1^.

The potential-dependent activity of each catalyst is compared in Fig. 7A. Fig. 7B displays the steady-state voltammogram for catalyst **1-Co** at 100 rpm to highlight the clean 2e^–^ reduction that occurs, which is followed by catalysis at more negative potentials. Apparent *n*_app_ values at –0.9 V *vs.* SHE are indicated in the bottom of each panel in Fig. 6 and shown graphically in Fig. 7A. The catalytic onset for each catalyst is consistent with the CVs shown in Fig. 4. Catalyst **1-Co** has an overpotential of ∼500 mV corresponding to a catalytic onset at *ca.* –0.9 V and a measured *n*_app_ of 2.5. In contrast, catalysts **2-Co** and **3-Co** show hydrogen evolution at significantly lower overpotentials. Indeed, *n*_app_ values of 4.1 and 6.6 were obtained for **2-Co** and **3-Co**, respectively, at –0.9 V *vs.* SHE. We note that a pyrazine moiety on the equatorial ligand plane not only results in a remarkable enhancement in catalytic activity at modest overpotentials, but installation of a second equatorial pyrazine extends this improvement. Levich plots of current density *versus* square root of rotation rate are linear, indicating that the catalysts are diffusional (Fig. S15†).

### Visible-light photoredox catalysis for generating hydrogen from water

**Fig. 8:**
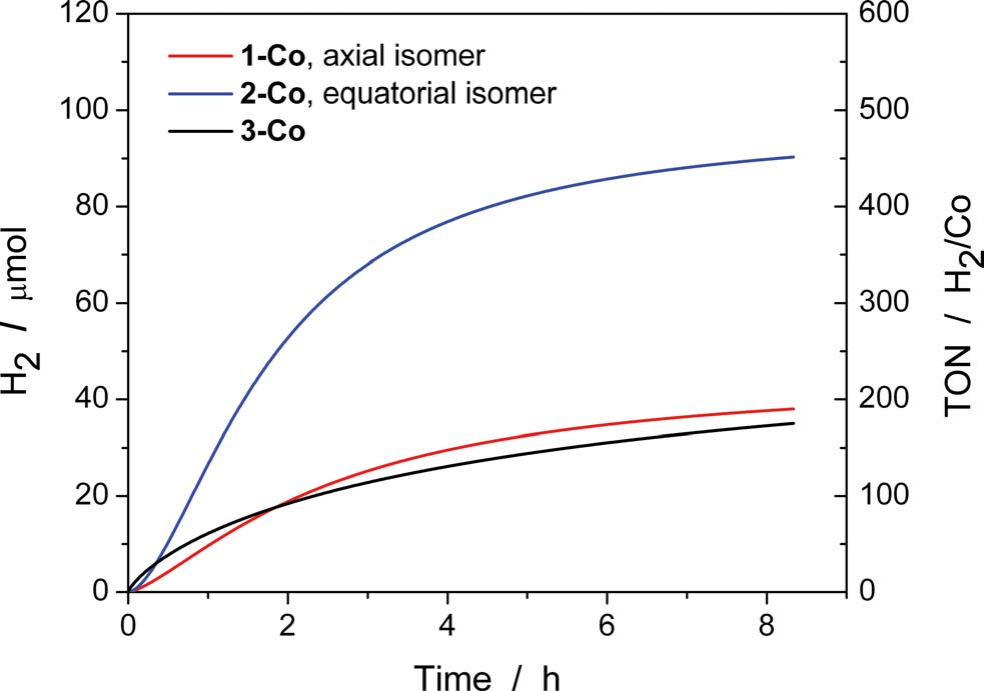
Photocatalytic H_2_ production of **1-Co**, **2-Co**, and **3-Co** with [Ru(bpy)_3_]^2+^ and ascorbic acid under 452 ± 10 nm (540 mW). H_2_ evolution and TON *vs.* time measured over 8 h of photocatalysis. Conditions: 2.0 × 10^–5^ M Co(ii) catalyst, 3.3 × 10^–4^ M [Ru(bpy)_3_]^2+^, and 0.3 M H_2_A/HA^–^ at pH 5.5.

With electrocatalytic data in hand, photocatalytic experiments were performed using [Ru(bpy)_3_]Cl_2_ as the prototypical molecular photosensitizer, ascorbic acid as the sacrificial electron donor, and the newly-prepared pyrazine-based cobalt complexes as molecular hydrogen evolution catalysts. All three components in the presence of visible light are required for hydrogen production as shown by appropriate control experiments.^10*c*^ These experiments were performed in a home-built 16-well reactor where evolved H_2_ was monitored in real-time using pressure transducers. At the end of the experiments, accumulated H_2_ was quantified by GC and MS sampling of the headspace. A thorough pH optimization study was performed revealing pH 5.5 to be optimal for all of the catalysts investigated (Fig. S16†). The kinetics of hydrogen production as well as the TONs (Fig. 8) were evaluated for each catalyst at constant ascorbic acid/ascorbate and [Ru(bpy)_3_]^2+^ concentrations. The equatorial isomer **2-Co** was shown to exhibit a greater than two-fold enhancement in catalytic hydrogen evolution activity with respect to the axial isomer **1-Co**. When compared with the previously reported catalysts [(CF_3_PY5Me_2_)Co(OH_2_)]^2+^ and [(PY5Me_2_)Co(OH_2_)]^2+^,^10*c*^ the equatorial isomer **2-Co**, [(eq-PY4PZMe_2_)Co(OH_2_)]^2+^, possesses a higher TON of ∼450 (H_2_/Co) under combinatorially optimized photocatalytic conditions over congeners that do not possess a pendant redox non-innocent ligand donor, consistent with the substantial improvement observed in its electrocatalytic behavior.

In contrast with previously reported observations on polypyridine systems,^17^ the decrease in observed hydrogen production over longer time periods is due to partial decomposition of both the [Ru(bpy)_3_]^2+^ photosensitizer and **2-Co** catalyst, as well as a slight increase in pH when catalysis is performed with ascorbate above pH 4.0, as catalysis was only restored up to 25% of the original value when an aliquot of fresh [Ru(bpy)_3_]^2+^ was added to the photolysis solution after the first cycle of irradiation. To this end, dynamic light scattering was used to confirm the absence of potentially catalytic nanoparticles above 0.5 nm in radius that could be formed by decomposition of the cobalt complexes (Fig. S17†).^73^ In addition, the mercury poisoning test was performed as previously described^20^ and no change in the hydrogen production yield was observed. The dynamic light scattering and mercury poisoning experiments confirmed the homogeneous nature of the photocatalytic mechanism. Hydrogen production using ascorbate and Ru(bpy)_3_^2+^ occurs *via* the reductive quenching mechanism as previously reported.^20^

We speculate that the decrease in activity might be attributed to the competitive binding of ascorbate to the cobalt center, since pyrazine is a weaker donor than pyridine. Indeed, anation by acetate binding has been shown to occur in the parent [(PY5Me_2_)Co(CH_3_CN)]^2+^ during electrocatalytic H_2_ production in acetonitrile and acetic acid.^10*b*^ Once the ascorbate is bound to the metal center, we anticipate the reduction for cobalt to become more negative and subsequently reduce the catalytic activity of the catalyst. While ascorbate represents a highly water-soluble reductive quencher of the [Ru(bpy)_3_]^2+^ excited state that is able to operate at a wide variety of pH conditions, we are currently working on finding alternative photosensitizers and quenchers to pair with the present cobalt catalysts to increase longer-term hydrogen generation.

Since the amount of hydrogen produced in the first 30 minutes of the photocatalytic experiments (Fig. 8) does not exceed 10 μmol H_2_, the pressure sensors utilized do not permit the precise measurement of the initial quantum yield of hydrogen production. Instead, the quantum yield of hydrogen production was measured using a He–Cd Laser as a radiation source at a wavelength of 442 nm. Solutions were irradiated through standard 1 cm path length cuvettes that possess a significantly smaller headspace volume than the photoreactors used in the high-throughput setup. The headspace was then sampled using gas chromatography *via* manual injections. Each catalyst was tested at 3 different power densities (30 mW, 45 mW, and 68 mW) for 30 minutes and the average is reported here. It was found that the quantum yield of hydrogen production^74^ is 0.26 ± 0.08% for **1-Co**, 0.49 ± 0.02% for **2-Co** and 0.10 ± 0.06% for **3-Co** in solutions containing 2.0 × 10^–5^ M Co(ii) catalyst, 3.3 × 10^–4^ M [Ru(bpy)_3_]^2+^, and 0.3 M H_2_A/HA^–^ at pH 5.5. These quantum yields are one order of magnitude smaller than the pentadentate and tetradentate cobalt polypyridine catalysts.^17,20^

Despite the low overpotential and high stability observed in electrocatalytic H_2_ production with **3-Co**, photocatalysis suffers markedly upon installation of a second equatorial pyrazine. Indeed, the data show that initial rates of H_2_ evolution by **3-Co** are higher at early time points compared to **2-Co** and **1-Co**. This observation is consistent with electrochemical CV activity measurements, where the onset of catalysis occurs at potentials near the initial metal reduction couple. Anation and lower overall stability offer a potential explanation for the observed behavior as ascorbate binding to **3-Co** is likely intensified with two relatively weak pyrazine donors.

### Electronic structure calculations

To supplement the experimental findings from electrocatalytic and photocatalytic studies, density functional theory (DFT, B3LYP-D2) calculations were performed on all three Co^2+^ catalysts and their one- and two-electron reduced species. The key structural features of the quartet Co^2+^ species **1-Co**, **2-Co**, and **3-Co** are reproduced by DFT calculations, in particular the Co–N_ax_ bond length (calc. 2.109 Å, exp. 2.1050(13) in **1-Co**; calc. 2.108 Å, exp. 2.099(2) in **2-Co**; calc. 2.111 Å, exp. 2.094(3) in **3-Co**). The main discrepancy is found in the calculated Co–O bond lengths, which are overestimated by 0.15, 0.16 and 0.17 Å, respectively. Additional test calculations on the extent to which the results are functional-dependent show similar trends (Tables S1–S3†). However, crystal packing and hydrogen bonded outer-sphere water molecules in the crystal lattice produce significant changes to the Co–O bond distances. For example, four unique positions are observed in the unit cell of Co(ii)–OH_2_ complex **3-Co** with Co–O bond distances of 1.958(4), 2.042(3), 2.016(3), and 2.049(3). Only one unique position for the cation is found in crystals of **1-Co** and **2-Co**, precluding experimental observation of the variability in Co–O bond distances due to packing effects for these species.

In order to interpret the electrochemical data in acetonitrile and provide further support of our assignments, we calculated the redox couples associated with the reduction of all three Co^2+^ complexes (*S* = 3/2) and their one- (*S* = 1) and two-electron (*S* = 3/2 and 3/2) reduced species. Experimentally, the non-aqueous cyclic voltammograms (CVs) in CH_3_CN are the same when using metal-aquo complexes or complexes formed by metalating each ligand in acetonitrile, therefore the Co-(CH_3_CN) complexes were employed in the calculations. In this case, the calculated redox potentials are in good agreement with the ones measured experimentally (Table 3). We also considered dissociation of the acetonitrile molecule after two-electron reductions, however, the redox potentials for the six-coordinate species are in better agreement (Table S7†).

**Table 3:** Calculated redox potentials (*V vs.* Fc^+^/Fc) for the Co^2+^ complexes (*S* = 3/2) and their one- (*S* = 1) and two-electron reduced species (*S* = 1/2 and 3/2) in solution (acetonitrile *via* C-PCM approach)

Complex	*E*_p1,c_ (*S* = 1)		*E*_p2,c_ (*S* = 1/2 and 3/2)		
	exptl	calcd	exptl	calcd	calcd
**1′-Co**, [(ax-PY4PZMe_2_)Co(CH_3_CN)]^2+^	–1.22	–1.28	–1.40	–1.39	–1.38
**2′-Co**, [(eq-PY4PZMe_2_)Co(CH_3_CN)]^2+^	–1.30	–1.34	–1.42	–1.42	–1.46
**3′-Co**, [(PY3PZ2Me_2_)Co(CH_3_CN)]^2+^	–1.18	–1.18^*a*^	–1.25	–1.25^*a*^	–1.25^*a*^

^*a*^This redox potential was used as reference in the isodesmic reactions, so it agrees by construction, and all other reduction potentials are calculated relative to this value.

**Fig. 9:**
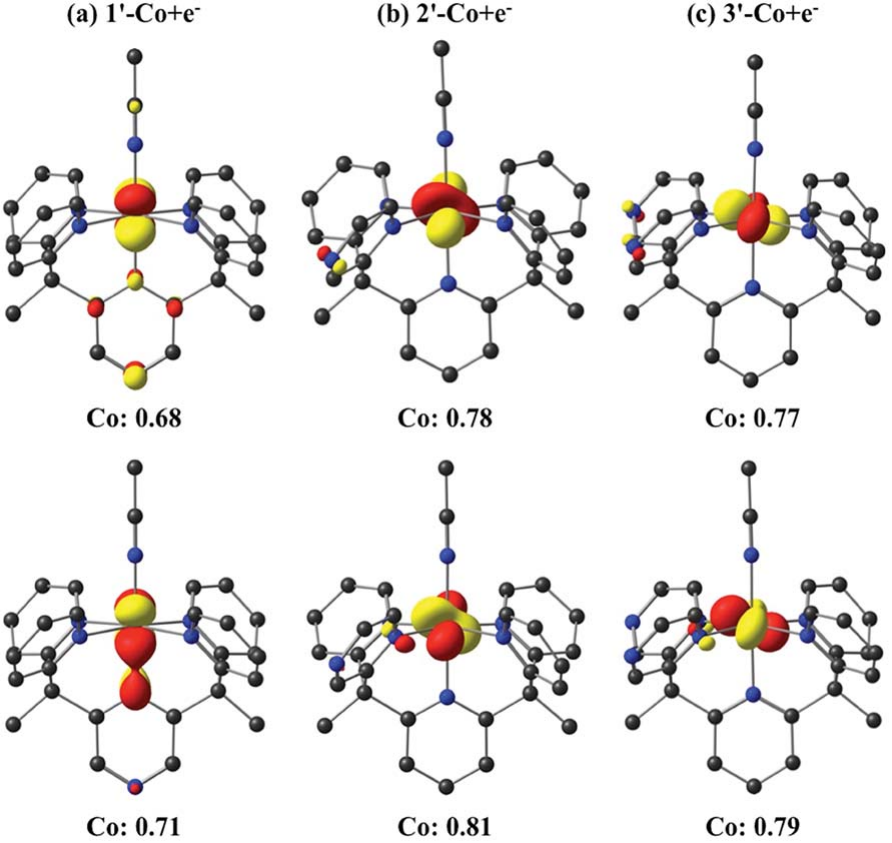
Isosurface (0.07 au) plots of the canonical highest occupied molecular orbitals (HOMOs, top) and β-spin localized orbitals (bottom) for **1′-Co + e^–^**, **2′-Co + e^–^** and **3′-Co + e^–^** (*S* = 1). The Löwdin population analyses are given for cobalt.

The one-electron reduced triplet species (**1′-Co** to **1′-Co + e^–^**, **2′-Co** to **2′-Co + e^–^**, and **3′-Co** to **3′-Co + e^–^**) was calculated to have the lowest free energy in acetonitrile for all three complexes (Table S5†). The canonical molecular orbitals, and the Löwdin population analysis^75^ suggest that reduction occurs at the metal-centered (Fig. S24–S26†). Interestingly, calculations suggest that the metal character of the highest occupied molecular orbital (HOMO) in **2′-Co + e^–^** (0.78) and **3′-Co + e^–^** (0.77) is dominant compared to **1′-Co + e^–^** (0.68, Fig. 9). The localized orbital bonding analysis (LOBA)^76^ using the Edmiston–Ruedenberg localized orbitals (Fig. S21–S23†)^77^ as well as the Mulliken spin population (Fig. S51–S53†) confirm that the one electron in **1-Co + e^–^** is shared between the cobalt center and the pyrazine ligand. Dissociation of the solvent molecule after the first reduction was also considered. In this case, the five-coordinate complexes were found to be slightly higher in energy in solution (Δ*G*_B3LYP/C-PCM_ ∼ +1.0 kcal mol^–1^; Δ*G*_B3LYP-D2/C-PCM_ ∼ +8.0 kcal mol^–1^), which may suggest that the Co^1+^ complexes remain six-coordinate in solution.

Following this result, we performed calculations on the six-coordinate Co^1+^ complexes and their one-electron reduced species (**1′-Co + 2e^–^**, **2′-Co + 2e^–^** and **3′-Co + 2e^–^**, respectively). In this case, the cobalt complexes can exist in both doublet and quartet states (Table S6†). DFT calculations suggest that these states are degenerate, however, the spin contamination for the doublet state is close to 2, which implies a mixing between the doublet and quartet states (*S* ^2^ ∼ 3.9 for the quartet state). The canonical molecular orbitals (Fig. S33–S38†) and the Mulliken spin population (Fig. S51–S53†) for all three cobalt complexes show that reduction occurs at the pyrazine ligand for both doublet and quartet states to yield a Co^I^L˙^–^ species. LOBA calculations also imply that the cobalt center remains in the +1 oxidation state (Fig. S27–S32†), which confirms that the extra electron goes onto the ligand. We also considered dissociation of the acetonitrile molecule for **1′-Co + 2e^–^**, **2′-Co + 2e^–^**, and **3′-Co + 2e^–^**. Calculations indicate that formation of a five-coordinate species can be competitive or lower in energy than the six-coordinate complexes. The precise numbers are somewhat functional-dependent (Table S6†). With B3LYP-D2 (*S* = 3/2), dissociation of the acetonitrile molecule from **1′-Co + 2e^–^**, **2′-Co + 2e^–^** and **3′-Co + 2e^–^** is found to be 4.7, 3.5 and 3.8 kcal mol^–1^ higher in energy, respectively, than the six coordinate species, although it is preferred with B3LYP. These observations suggest that a competition between five- and six-coordinate species may exist in solution and play a role in catalysis. Again, DFT calculations on the five-coordinate cobalt species show that reduction occurs at the pyrazine ligand for all three cobalt species (Fig. S39–S53†). It is worth mentioning that the lowest unoccupied molecular orbitals (LUMOs) for all three-cobalt complexes (before or after dissociation of the solvent molecule) are ligand-centered, which implies that the three-electron reduced species will give a Co^I^L^2–^ complex.

Note that in the present study, dissociation of one equatorial ligand (pyrazine or pyridine) was not considered. The full examination of these steps is beyond the scope of this study and a more comprehensive follow-up computational study is underway.

## Concluding remarks

Inspired by mononuclear metalloenzymes that operate in concert with precisely positioned redox-active cofactors to orchestrate multielectron reactions, we have presented the synthesis and evaluation of a series of mononuclear cobalt catalysts for proton reduction bearing redox-active pyrazine reservoirs at well-defined positions within a structurally homologous motif. Notably, these systems operate under protein-compatible conditions in water at neutral, physiological pH. We also prepared and characterized the isostructural analogs coordinated by redox-inactive zinc(ii) to disentangle the contributions of ligand-based and metal-based redox chemistry of these systems. The cobalt complexes with an equatorial pyrazine functionality are competent molecular electrocatalysts under soluble, diffusion-limited conditions using a glassy carbon electrode and in aqueous media with a Hg pool electrode. No sign of deactivation is observed in pH 7 phosphate buffer, and the Faradaic efficiency has been quantified to ∼100% for hydrogen production for the series of cobalt catalysts. More importantly, they can function as molecular photocatalysts for hydrogen production from aqueous protons under visible light irradiation when combined with a molecular photosensitizer [Ru(bpy)_3_]^2+^.

In contrast to Zn(ii) complexes of the parent PY5Me_2_ ligand and derivatives containing peripheral substitutions on the pyridine ring, which are electrochemically silent in non-aqueous cyclic voltammetry studies,^10^ the redox non-innocent nature of the pyrazine donors is clearly observed in a family of complexes, **1-Zn**, **2-Zn**, and **3-Zn**, that are isostructural but vary in the location of the redox-active pyrazine reservoir(s). Moreover, from pH-dependent cyclic voltammetry measurements on these complexes containing redox-inactive Zn(ii) centers, we have shown that reduction of pyrazine moieties in the ligand framework is concomitant with protonation, likely at the distal pyrazine nitrogen. Additionally, square wave voltammetry of the cobalt complexes was used to authenticate the assignments of a closely-spaced Co(ii/i) reduction and a ligand-based reduction for a combined two electrons in comparison to the one-electron peak of the Co(iii/ii) couple.

The position of the redox non-innocent pyrazine group(s) is critical to catalyst performance, as evidenced by comparison of the axial and equatorial isomers of Co(ii) catalysts for hydrogen production containing a single pyrazine substitution in a PY5Me_2_-type scaffold. Indeed, the equatorial isomer **2-Co** is superior to the axial isomer **1-Co** for hydrogen production from protons in both organic and aqueous solution. Electronic structure differences resulting from positioning of the redox non-innocent pyrazine cofactor relative to the catalytic cobalt center play an important role in this regard. Indeed, the first reductions of **1-Co** and **2-Co** occur at similar potentials (*ca.* –0.7 V *vs.* SHE), precluding a difference in reactivity based strictly on a thermodynamic driving force argument. Interestingly, DFT calculations suggest that the one-electron reduced species **2′-Co + e^–^** and **3′-Co + e^–^** have significantly more metal character than **1′-Co + e^–^**.

The two-electron reduced species suggest a competition between five- and six-coordinate species, which may play a role in catalysis. For instance, we have previously shown that Co(ii) complexes supported by tetradentate polypyridine ligands yield more active photocatalytic compositions than similar catalysts with pentadentate ligands and still retain high catalyst stability.^20^ In particular, complexes with tetradentate ligands that promote *cis* open coordination sites appear to be more active for hydrogen evolution than catalysts with *trans* open sites.^20,21,78^ Given this structure–activity relationship and the favorable energetics for ligand dissociation upon reduction of **2-Co**, we hypothesize that two open *cis* coordination sites are available for catalysis *via* an electron transfer-induced ligand dissociation mechanism. Electro- and photocatalytic measurements are consistent with this scenario in which the equatorial isomer far outperforms the axial isomer. In contrast, dissociation of an equatorial pyridine does not appear to be readily accessible for **1-Co**, and thus the axial redox non-innocent pyrazine acts as an unproductive electron sink. In this case, protonation to form the key cobalt-hydride intermediate, postulated in structurally similar cobalt polypyridine catalysts,^10,14,17^ may be the rate-limiting step for **1-Co**. Additionally, the 1H^+^/2e^–^ pH dependence of the second reduction of **1-Co** suggests a third reduction may be necessary to initiate catalysis. On the other hand, a single, multielectron catalytic feature with pH dependence consistent with 1H^+^/1e^–^ events is observed for both **2-Co** and **3-Co**. Catalysis begins at this initial redox event rather than at the more reducing potentials required for **1-Co**. Lower catalytic overpotentials for **2-Co** and **3-Co** are indeed verified by controlled potential electrolysis and H_2_ quantification.

Finally, in addition to these electronic structure considerations, we also note that catalyst stability, particularly in aqueous media, is an important factor under both electrocatalysis and photocatalysis. This situation is illustrated by **3-Co**, which possesses two relatively weak pyrazine donors. In this case, electron transfer-induced ligand dissociation may lead to enhanced deactivation by anation. While **3-Co** showed high stability in aqueous phosphate buffer, its activity under photocatalytic conditions (as well as in acetonitrile with added chloroacetic acid) is diminished over time. As such, the comparably low overpotential of **2-Co**, coupled with its high activity and superior stability, identifies it as the best catalyst of this series. To our knowledge, this is the first example of isomers involving redox-active ligands displaying significantly different catalyst reactivities.

In closing, the collective data on this series of cobalt complexes supported by structurally homologous pentadentate ligands show marked differences in catalytic hydrogen production from water by varying the arrangement of a redox-active pyrazine ligand relative to a single metal site. This synthetic system has conceptual parallels to mononuclear metalloenzymes, such as galactose oxidase, copper amine oxidase, and [Fe]-hydrogenase, that combine a single metal center and pendant redox-active organic cofactor with strict conformational demands, and reveals the fine electronic balance between metal center and ligand in dictating reactivity and function. The utility of isostructural control complexes with redox-inactive metal ions, such as Zn(ii), has aided in disentangling the contributions from metal-centered and ligand-centered redox processes in such systems. These results highlight the importance of electronic structure considerations regarding the placement of redox non-innocent ligands in catalyst design, which has broader implications for the use of electron–hole reservoirs for multielectron chemical transformations.

## Conflict of interest

The authors declare no competing financial interest.

## References

[cit1] Meyer T. J. (1989). Acc. Chem. Res..

[cit2] Esswein A. J., Nocera D. G. (2007). Chem. Rev..

[cit3] Bhugun I., Lexa D., Saveant J.-M. (1996). J. Am. Chem. Soc..

[cit4] Fisher B. J., Eisenberg R. (1980). J. Am. Chem. Soc..

[cit5] Houlding V., Geiger T., Kölle U., Grätzel M. (1982). J. Chem. Soc., Chem. Commun..

[cit6] Kellet R. M., Spiro T. G. (1985). Inorg. Chem..

[cit7] Connolly P., Espenson J. H. (1986). Inorg. Chem..

[cit8] Bernhardt P. V., Jones L. A. (1999). Inorg. Chem..

[cit9] Bigi J. P., Hanna T. E., Harman W. H., Chang A., Chang C. J. (2010). Chem. Commun..

[cit10] Sun Y., Bigi J. P., Piro N. A., Tang M. L., Long J. R., Chang C. J. (2011). J. Am. Chem. Soc..

[cit11] Stubbert B. D., Peters J. C., Gray H. B. (2011). J. Am. Chem. Soc..

[cit12] McNamara W. R., Han Z., Yin C.-J., Brennessel W. W., Holland P. L., Eisenberg R. (2012). Proc. Natl. Acad. Sci. U. S. A..

[cit13] McCrory C. C. L., Uyeda C., Peters J. C. (2012). J. Am. Chem. Soc..

[cit14] Singh W. M., Baine T., Kudo S., Tian S., Ma X. A. N., Zhou H., DeYonker N. J., Pham T. C., Bollinger J. C., Baker D. L., Yan B., Webster C. E., Zhao X. (2012). Angew. Chem., Int. Ed..

[cit15] Leung C.-F., Ng S.-M., Ko C.-C., Man W.-L., Wu J., Chen L., Lau T.-C. (2012). Energy Environ. Sci..

[cit16] Bachmann C., Guttentag M., Spingler B., Alberto R. (2013). Inorg. Chem..

[cit17] Nippe M., Khnayzer R. S., Panetier J. A., Zee D. Z., Olaiya B. S., Head-Gordon M., Chang C. J., Castellano F. N., Long J. R. (2013). Chem. Sci..

[cit18] Zhang P., Wang M., Gloaguen F., Chen L., Quentel F., Sun L. (2013). Chem. Commun..

[cit19] Mondal B., Sengupta K., Rana A., Mahammed A., Botoshansky M., Dey S. G., Gross Z., Dey A. (2013). Inorg. Chem..

[cit20] Khnayzer R. S., Thoi V. S., Nippe M., King A. E., Jurss J. W., El Roz K. A., Long J. R., Chang C. J., Castellano F. N. (2014). Energy Environ. Sci..

[cit21] Tong L., Zong R., Thummel R. P. (2014). J. Am. Chem. Soc..

[cit22] Xie J., Zhou Q., Li C., Wang W., Hou Y., Zhang B., Wang X. (2014). Chem. Commun..

[cit23] Collin J. P., Jouaiti A., Sauvage J. P. (1988). Inorg. Chem..

[cit24] Efros L. L., Thorp H. H., Brudvig G. W., Crabtree R. H. (1992). Inorg. Chem..

[cit25] Begum A., Moula G., Sarkar S. (2010). Chem.–Eur. J..

[cit26] Luca O. R., Konezny S. J., Blakemore J. D., Colosi D. M., Saha S., Brudvig G. W., Batista V. S., Crabtree R. H. (2012). New J. Chem..

[cit27] Han Z., McNamara W. R., Eum M.-S., Holland P. L., Eisenberg R. (2012). Angew. Chem., Int. Ed..

[cit28] Kagalwala H. N., Gottlieb E., Li G., Li T., Jin R., Bernhard S. (2013). Inorg. Chem..

[cit29] Cui H.-H., Wang J.-Y., Hu M.-Q., Ma C.-B., Wen H.-M., Song X.-W., Chen C.-N. (2013). Dalton Trans..

[cit30] Gross M. A., Reynal A., Durrant J. R., Reisner E. (2014). J. Am. Chem. Soc..

[cit31] Mejia-Rodriguez R., Chong D., Reibenspies J. H., Soriaga M. P., Darensbourg M. Y. (2004). J. Am. Chem. Soc..

[cit32] Wang Z., Liu J., He C., Jiang S., Åkermark B., Sun L. (2007). Inorg. Chim. Acta.

[cit33] Gao W., Sun J., Åkermark T., Li M., Eriksson L., Sun L., Åkermark B. (2010). Chem.–Eur. J..

[cit34] Quentel F., Passard G., Gloaguen F. (2012). Energy Environ. Sci..

[cit35] Karunadasa H. I., Chang C. J., Long J. R. (2010). Nature.

[cit36] Karunadasa H. I., Montalvo E., Sun Y., Majda M., Long J. R., Chang C. J. (2012). Science.

[cit37] Denisov I. G., Makris T. M., Sligar S. G., Schlichting I. (2005). Chem. Rev..

[cit38] Jazdzewski B. A., Tolman W. B. (2000). Coord. Chem. Rev..

[cit39] Verma P., Pratt R. C., Storr T., Wasinger E. C., Stack T. D. P. (2011). Proc. Natl. Acad. Sci. U. S. A..

[cit40] Dawkes H. C., Phillips S. E. V. (2001). Curr. Opin. Struct. Biol..

[cit41] Bartoschek S., Buurman G., Thauer R. K., Geierstanger B. H., Weyrauch J. P., Griesinger C., Nilges M., Hutter M. C., Helms V. (2001). ChemBioChem.

[cit42] Shima S., Pilak O., Vogt S., Schick M., Stagni M. S., Meyer-Klaucke W., Warkentin E., Thauer R. K., Ermler U. (2008). Science.

[cit43] Stubbe J., van der Donk W. A. (1998). Chem. Rev..

[cit44] Toscano M. D., Woycechowsky K. J., Hilvert D. (2007). Angew. Chem., Int. Ed..

[cit45] Nenner I., Schulz G. J. (1975). J. Chem. Phys..

[cit46] Song J. K., Lee N. K., Kim S. K. (2002). J. Chem. Phys..

[cit47] Kershaw Cook L. J., Tuna F., Halcrow M. A. (2013). Dalton Trans..

[cit48] Ford P., Rudd D. F. P., Gaunder R., Taube H. (1968). J. Am. Chem. Soc..

[cit49] Toma H. E., Malin J. M. (1973). Inorg. Chem..

[cit50] Schrauzer G. N., Mayweg V. (1962). J. Am. Chem. Soc..

[cit51] Königsmann M., Donati N., Stein D., Schonberg H., Harmer J., Sreekanth A., Grutzmacher H. (2007). Angew. Chem., Int. Ed..

[cit52] Praneeth V. K. K., Ringenberg M. R., Ward T. R. (2012). Angew. Chem., Int. Ed..

[cit53] Blanchard S., Derat E., Murr M. D.-E., Fensterbank L., Malacria M., Mouriès-Mansuy V. (2012). Eur. J. Inorg. Chem..

[cit54] Luca O. R., Crabtree R. H. (2013). Chem. Soc. Rev..

[cit55] APEX2, v. 2009, Bruker Analytical X-Ray Systems, Inc, Madison, WI, 2009.

[cit56] Sheldrick G. M., SADABS, Version 2.03, Bruker Analytical X-ray Systems, Inc, Madison, WI, 2000.

[cit57] (c) Sheldrick G. M., SHELXL-97: program for crystal structure determination, University of Göttingen, Göttingen, Germany, 1997.

[cit58] Shao Y., Gan Z., Epifanovsky E., Gilbert A. T. B., Wormit M., Kussmann J., Lange A. W., Behn A., Deng J., Feng X., Ghosh D., Goldey M., Horn P. R., Jacobson L. D., Kaliman I., Khaliullin R. Z., Kuś T., Landau A., Liu J., Proynov E. I., Rhee Y. M., Richard R. M., Rohrdanz M. A., Steele R. P., Sundstrom E. J., Woodcock H. L., Zimmerman P. M., Zuev D., Albrecht B., Alguire E., Austin B., Beran G. J. O., Bernard Y. A., Berquist E., Brandhorst K., Bravaya K. B., Brown S. T., Casanova D., Chang C.-M., Chen Y., Chien S. H., Closser K. D., Crittenden D. L., Diedenhofen M., DiStasio R. A., Do H., Dutoi A. D., Edgar R. G., Fatehi S., Fusti-Molnar L., Ghysels A., Golubeva-Zadorozhnaya A., Gomes J., Hanson-Heine M. W. D., Harbach P. H. P., Hauser A. W., Hohenstein E. G., Holden Z. C., Jagau T.-C., Ji H., Kaduk B., Khistyaev K., Kim J., Kim J., King R. A., Klunzinger P., Kosenkov D., Kowalczyk T., Krauter C. M., Lao K. U., Laurent A. D., Lawler K. V., Levchenko S. V., Lin C. Y., Liu F., Livshits E., Lochan R. C., Luenser A., Manohar P., Manzer S. F., Mao S.-P., Mardirossian N., Marenich A. V., Maurer S. A., Mayhall N. J., Neuscamman E., Oana C. M., Olivares-Amaya R., O'Neill D. P., Parkhill J. A., Perrine T. M., Peverati R., Prociuk A., Rehn D. R., Rosta E., Russ N. J., Sharada S. M., Sharma S., Small D. W., Sodt A., Stein T., Stück D., Su Y.-C., Thom A. J. W., Tsuchimochi T., Vanovschi V., Vogt L., Vydrov O., Wang T., Watson M. A., Wenzel J., White A., Williams C. F., Yang J., Yeganeh S., Yost S. R., You Z.-Q., Zhang I. Y., Zhang X., Zhao Y., Brooks B. R., Chan G. K. L., Chipman D. M., Cramer C. J., Goddard W. A., Gordon M. S., Hehre W. J., Klamt A., Schaefer H. F., Schmidt M. W., Sherrill C. D., Truhlar D. G., Warshel A., Xu X., Aspuru-Guzik A., Baer R., Bell A. T., Besley N. A., Chai J.-D., Dreuw A., Dunietz B. D., Furlani T. R., Gwaltney S. R., Hsu C.-P., Jung Y., Kong J., Lambrecht D. S., Liang W., Ochsenfeld C., Rassolov V. A., Slipchenko L. V., Subotnik J. E., Van Voorhis T., Herbert J. M., Krylov A. I., Gill P. M. W., Head-Gordon M. (2014). Mol. Phys..

[cit59] Becke A. D. (1993). J. Chem. Phys..

[cit60] Lee C., Yang W., Parr R. G. (1988). Phys. Rev. B.

[cit61] Weigend F., Ahlrichs R. (2005). Phys. Chem. Chem. Phys..

[cit62] Pulay P. (1980). Chem. Phys. Lett..

[cit63] Van Voorhis T., Head-Gordon M. (2002). Mol. Phys..

[cit64] Lange A. W., Herbert J. M. (2010). J. Chem. Phys..

[cit65] Grimme S. (2006). J. Comput. Chem..

[cit66] Grimme S. (2012). Chem.–Eur. J..

[cit67] Khnayzer R. S., McCusker C. E., Olaiya B. S., Castellano F. N. (2013). J. Am. Chem. Soc..

[cit68] Bechlars B. D., D'Alessandro M., Jenkins D. M., Iavarone A. T., Glover S. D., Kubiak C. P., Long J. R. (2010). Nat. Chem..

[cit69] Ünal E. A., Wiedemann D., Seiffert J., Boyd J. P., Grohmann A. (2012). Tetrahedron Lett..

[cit70] Seitz M., Kaiser A., Powell D. R., Borovik A. S., Reiser O. (2004). Adv. Synth. Catal..

[cit71] Klein B., Spoerri P. E. (1951). J. Am. Chem. Soc..

[cit72] Felton G. A. N., Glass R. S., Lichtenberger D. L., Evans D. H. (2006). Inorg. Chem..

[cit73] Anxolabéhère-Mallart E., Costentin C., Fournier M., Nowak S., Robert M., Savéant J.-M. (2012). J. Am. Chem. Soc..

[cit74] Khnayzer R. S., Martin D. R., Codding C. L., Castellano F. N. (2015). Rev. Sci. Instrum..

[cit75] Löwdin P. O. (1950). J. Chem. Phys..

[cit76] Thorn A. J. W., Sundstrom E. J., Head-Gordon M. (2009). Phys. Chem. Chem. Phys..

[cit77] Edmiston C., Ruedenberg K. (1963). Rev. Mod. Phys..

[cit78] Additional investigations on this point may be necessary as catalyst stability could explain lower activities observed for representative catalysts having *trans* open coordination sites

