# Supplementary material for: Bioinspired design of redox-active ligands for multielectron catalysis: effects of positioning pyrazine reservoirs on cobalt for electro- and photocatalytic generation of hydrogen from water
Source: Chem Sci. 2015 Jun 9;6(8):4954–72. doi: 10.1039/c5sc01414j (PMC5664355; doi:10.1039/c5sc01414j)
Supplement: Supplementary file 1 [file SC-006-C5SC01414J-s001.pdf]

Electronic Supplementary Material (ESI) for Chemical Science.
This journal is © The Royal Society of Chemistry 2015

Supporting Information

Bioinspired Design of Redox-Active Ligands for Multielectron Catalysis: Effects of Positioning Pyrazine Reservoirs on Cobalt for Electro- and Photocatalytic Generation of Hydrogen from Water

Jonah W. Jurss,[a,c,e] Rony S. Khnayzer,[f,g] Julien A. Panetier,[a,d] Karim A. El Roz,[f] Eva M. Nichols,[a,e] Martin Head-Gordon,*[a,e] Jeffrey R. Long,*[a,d] Felix N. Castellano,*[f] Christopher J. Chang*[a,b,e,h]

[a]Department of Chemistry and [b]Department of Molecular and Cell Biology, University of California, Berkeley, California 94720, USA; [c]Department of Chemistry and Biochemistry, University of Mississippi, University, MS 38677, USA; [d]Materials Sciences Division and [e]Chemical Sciences Division, Lawrence Berkeley National Laboratory, Berkeley, California 94720, USA; [f]Department of Chemistry, North Carolina State University, Raleigh, NC 27695-8204; [g]Department of Natural Sciences, Lebanese American University, Chouran, Beirut 1102-2801, Lebanon; [h]Howard Hughes Medical Institute, University of California, Berkeley, California 94720, USA.

chrischang@berkeley.edu, jrlong@berkeley.edu, fncastel@ncsu.edu, mhg@cchem.berkeley.edu

1

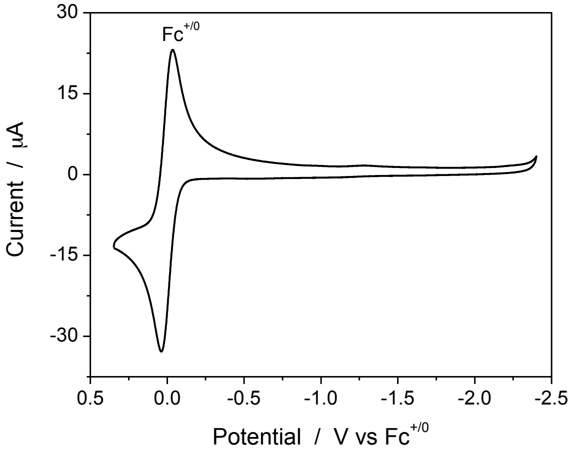

2

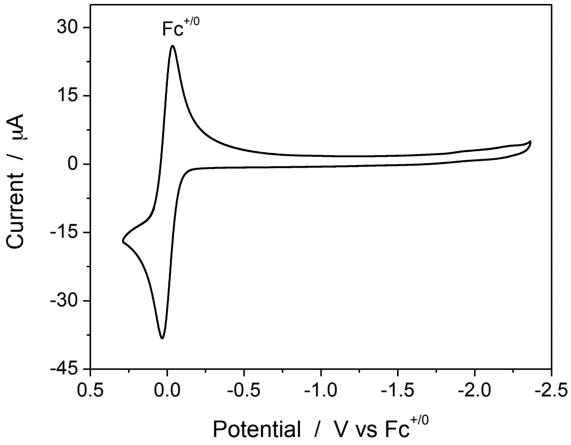

3

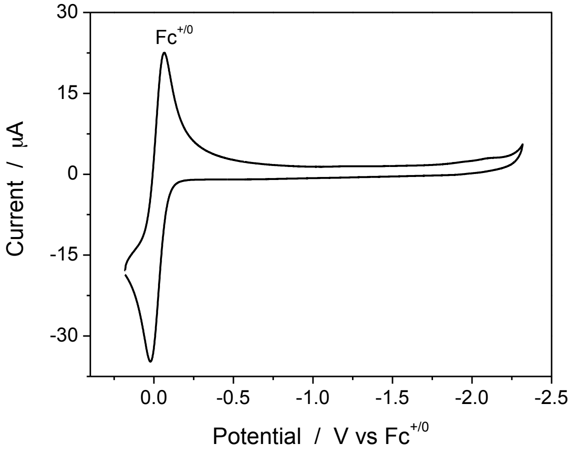

Figure S1. Cyclic voltammograms of ligands **1**, **2**, and **3** at 1 mM concentrations in 0.1 M Bu_4PF_6 CH_3CN with the ferrocene peak included as the internal reference, scan rate = 100 mV/s.

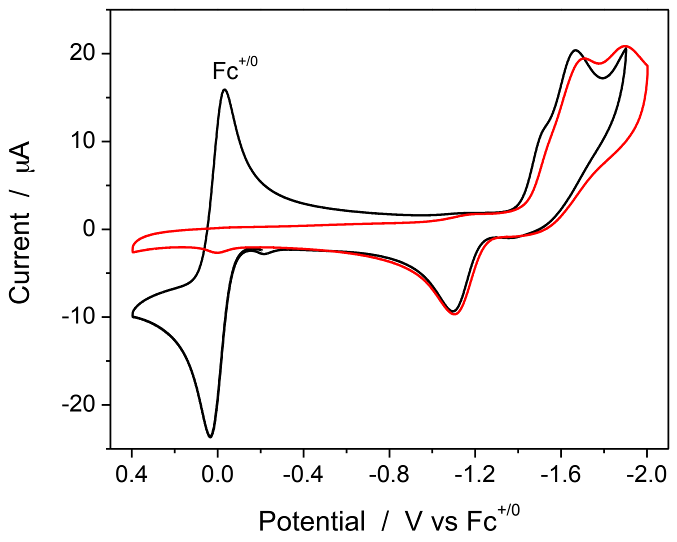

Figure S2. Cyclic voltammograms of 1 mM [(*ax*-PY4PZMe$_2$)Zn(OH$_2$)](OTf)$_2$ (**1-Zn**) in 0.1 M *n*Bu$_4$PF$_6$ CH$_3$CN with and without the internal ferrocene reference, scan rate = 100 mV/s.

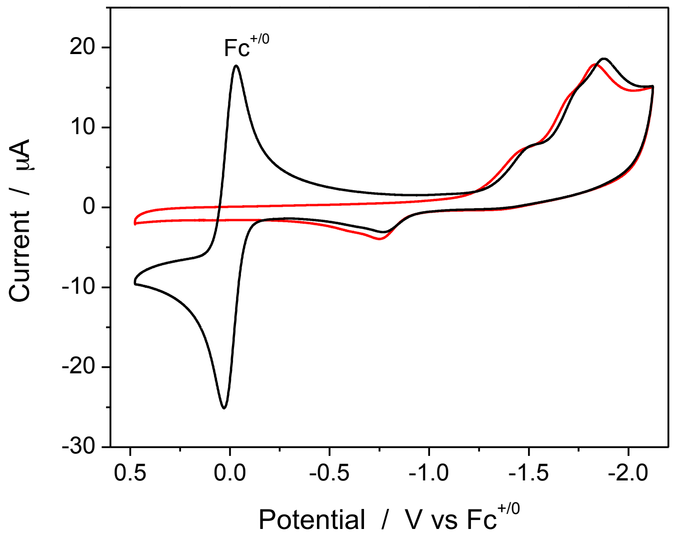

Figure S3. Cyclic voltammograms of 1 mM [(*eq*-PY4PZMe$_2$)Zn(OH$_2$)](OTf)$_2$ (**2-Zn**) in 0.1 M *n*Bu$_4$PF$_6$ CH$_3$CN with and without the internal ferrocene reference, scan rate = 100 mV/s.

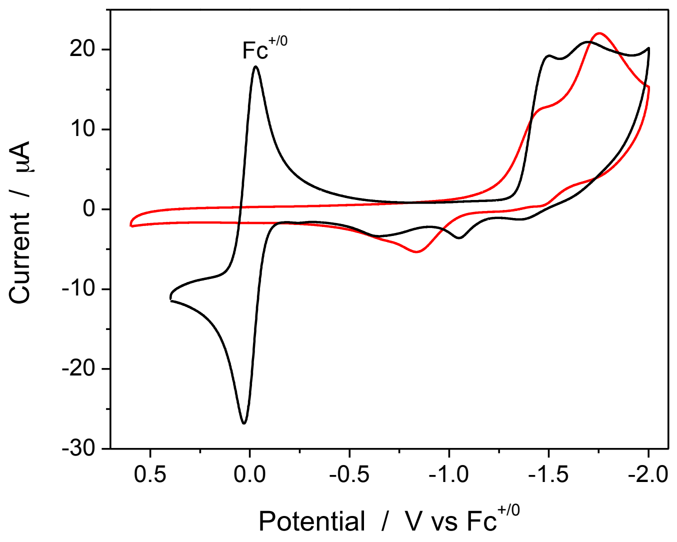

Figure S4. Cyclic voltammograms of 1 mM [(PY3PZ2Me$_2$)Zn(OH$_2$)](OTf)$_2$ (**3-Zn**) in 0.1 M *n*Bu$_4$PF$_6$ CH$_3$CN with and without the internal ferrocene reference, scan rate = 100 mV/s.

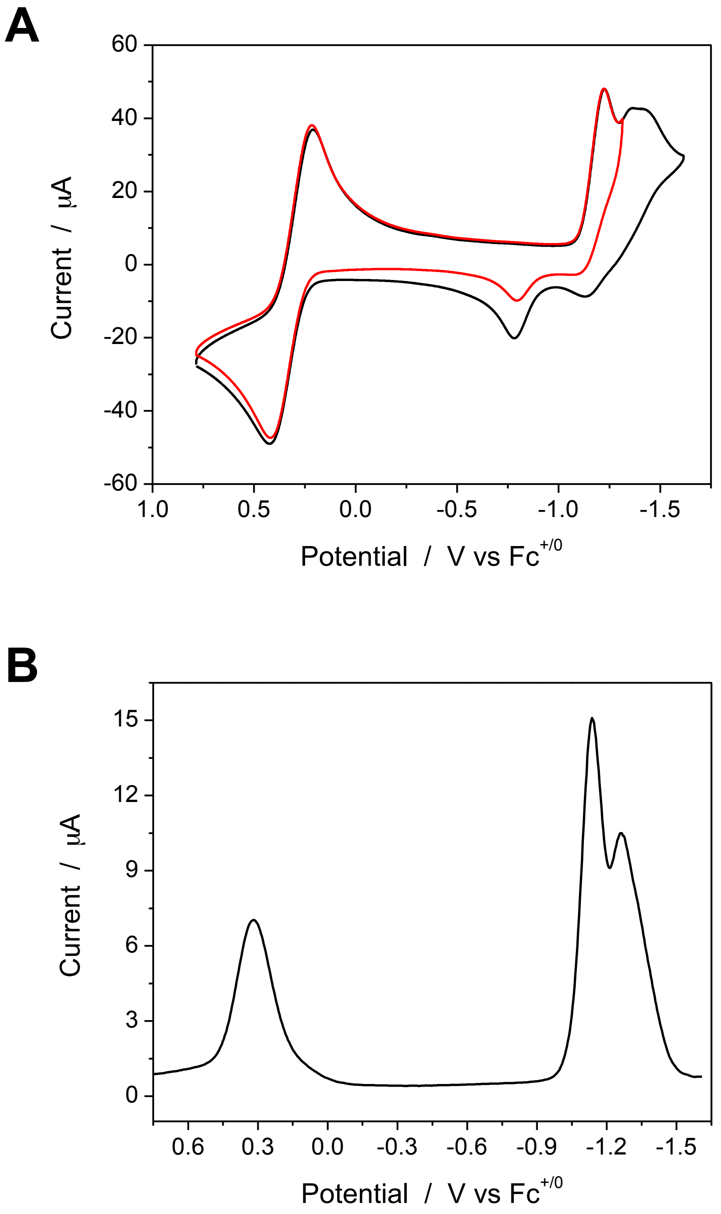

Figure S5, A. Cyclic voltammograms of 1 mM [(*ax*-PY4PZMe$_2$)Co(CH$_3$CN)](OTf)$_2$ (**1-Co**) in 0.1 M *n*Bu$_4$PF$_6$ CH$_3$CN, scan rate = 100 mV/s. **B.** Square wave voltammogram of the same solution.

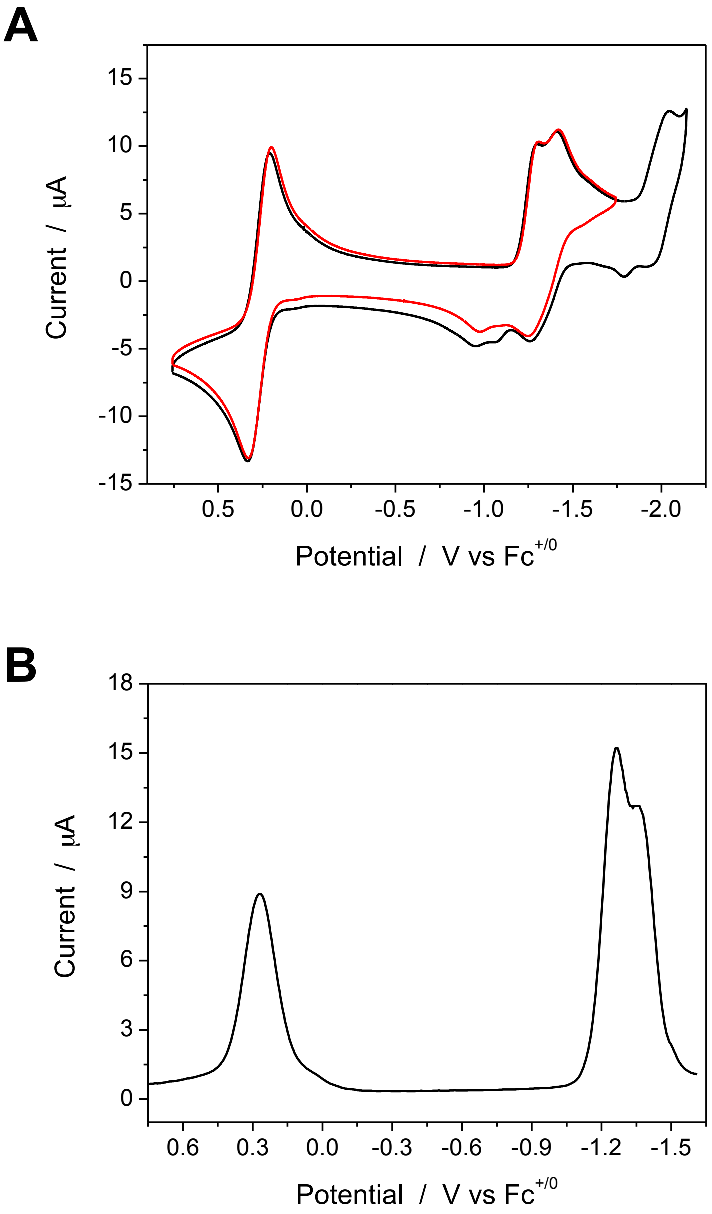

Figure S6, A. Cyclic voltammograms of 1 mM [(*eq*-PY4PZMe$_2$)Co(CH_3CN)](OTf)$_2$ (**2-Co**) in 0.1 M *n*Bu$_4$PF$_6$ CH_3CN, scan rate = 100 mV/s. **B.** Square wave voltammogram of the same solution.

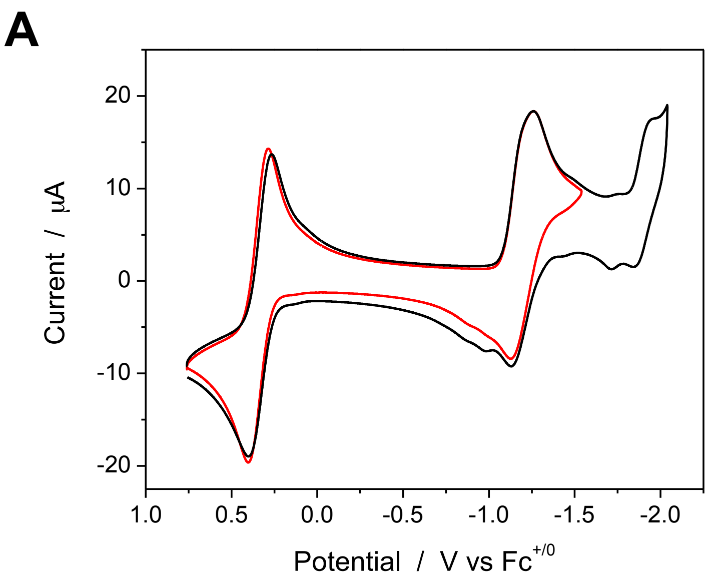

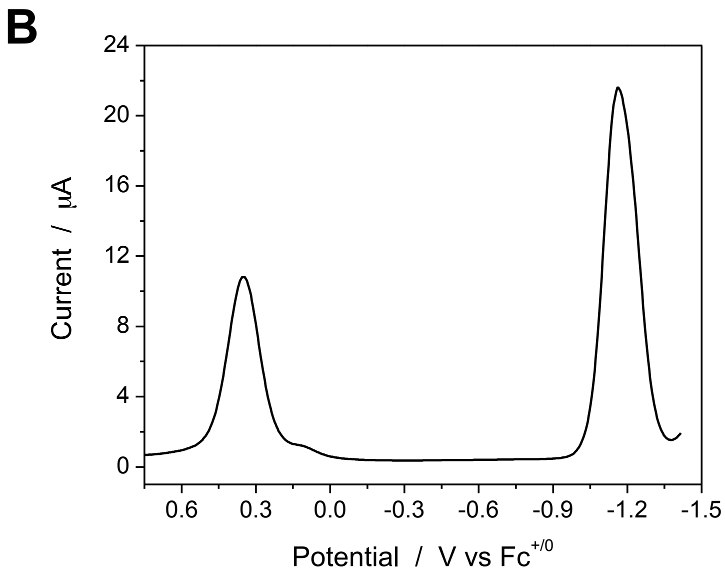

Figure S7, A. Cyclic voltammograms of 1 mM [(PY3PZ2Me$_2$)Co(CH$_3$CN)](OTf)$_2$ (**3-Co**) in 0.1 M *n*Bu$_4$PF$_6$ CH$_3$CN, scan rate = 100 mV/s. **B.** Square wave voltammogram of the same solution.

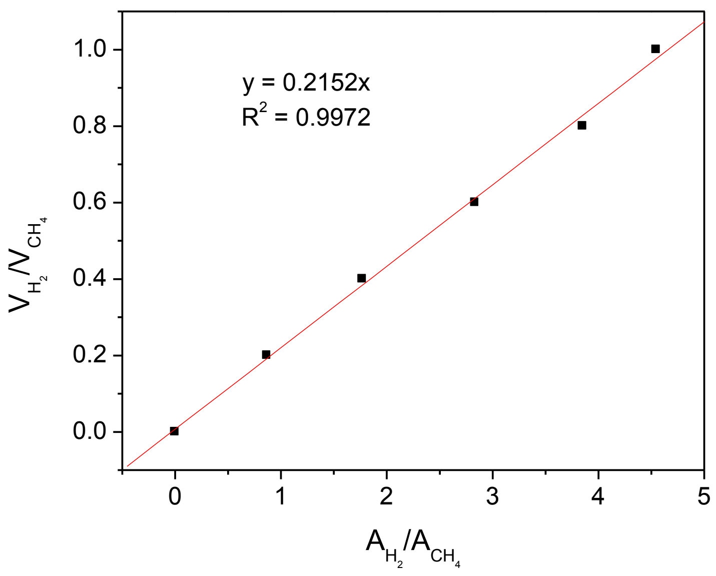

Figure S8. Calibration curve for H_2 quantification by gas chromatography using CH_4 (5 mL) as an internal standard (V: gas volume; A: integrated area of peak signal in the gas chromatogram).

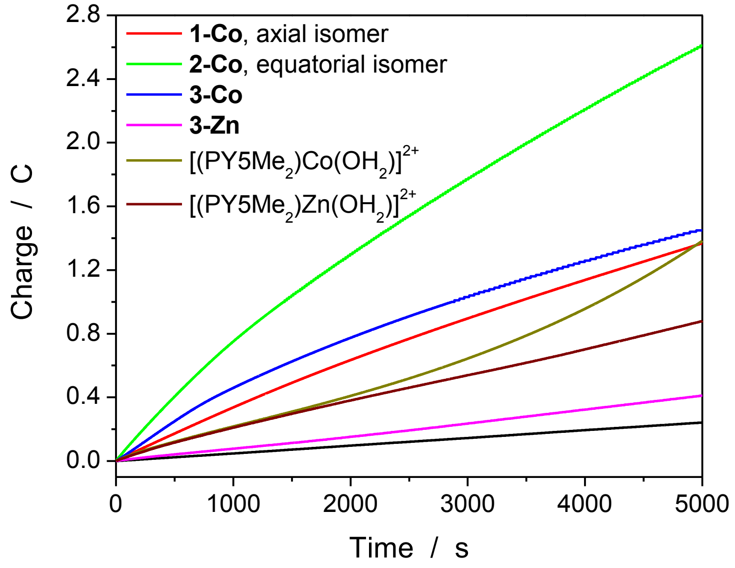

Figure S9. Controlled potential electrolyses of 1 mM complexes in 0.1 M nBu$_4$PF$_6$ CH_3CN with 100 eq. of chloroacetic acid at a fixed potential of -1.5 V vs Fc^+/Fc. The charge-time profile of **3-Zn** is representative of the series of zinc complexes. The black trace is the background under these conditions. Note: $[(PY5Me_2)Co(OH_2)](OTf)_2$ appears to adsorb onto the electrode during electrolysis and its activity begins to increase over time.

1-Co

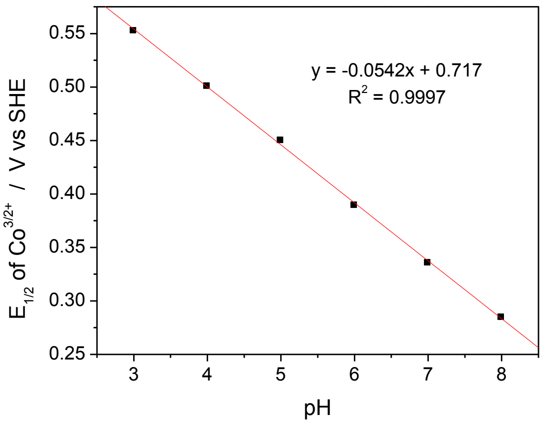

2-Co

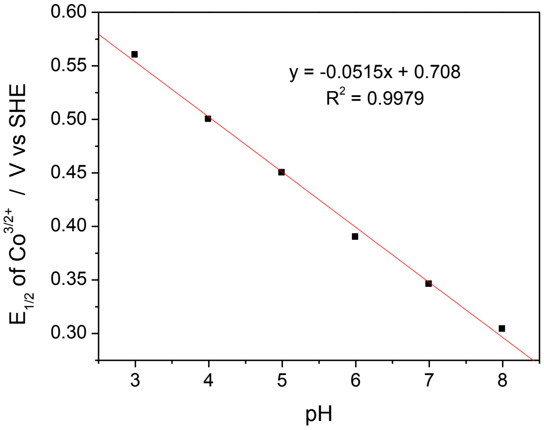

3-Co

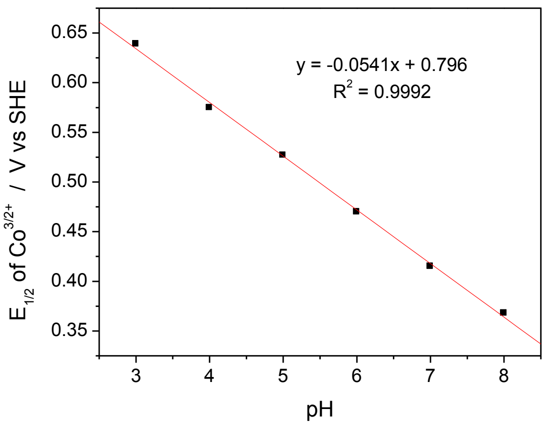

Figure S10. $E_{1/2}$ of Co(III/II) couple versus pH for **1-Co**, **2-Co,** and **3-Co**. *Conditions*: 0.9 mM catalyst, 0.03 M buffer, 0.1 M KNO_3, glassy carbon electrode (3 mm dia).

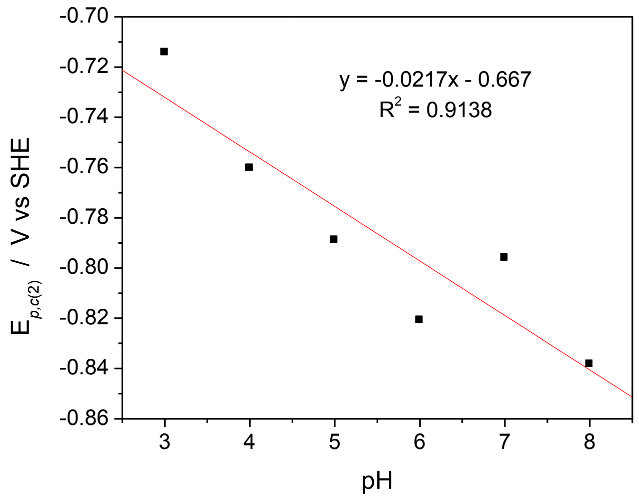

Figure S11. Second reductive peak potential ($E_{p,c(2)}$) vs pH for **1-Co**. *Conditions*: 0.9 mM catalyst, 0.03 M buffer, 0.1 M KNO_3, glassy carbon electrode (3 mm dia).

1-Zn

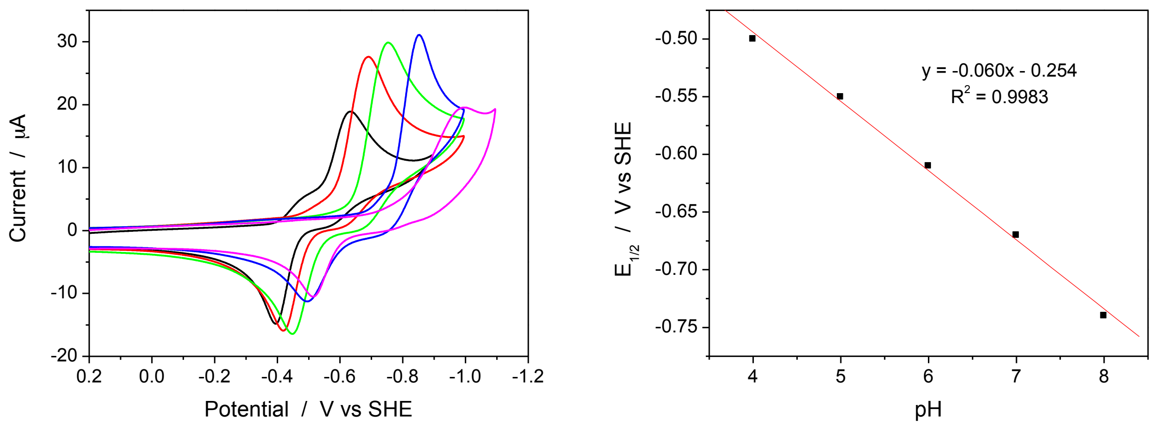

Figure S12. $E_{1/2}$ of skewed ligand-based redox couple versus pH for **1-Zn**. *Conditions*: 0.9 mM complex, 0.03 M buffer, 0.1 M KNO_3, glassy carbon electrode (3 mm dia).

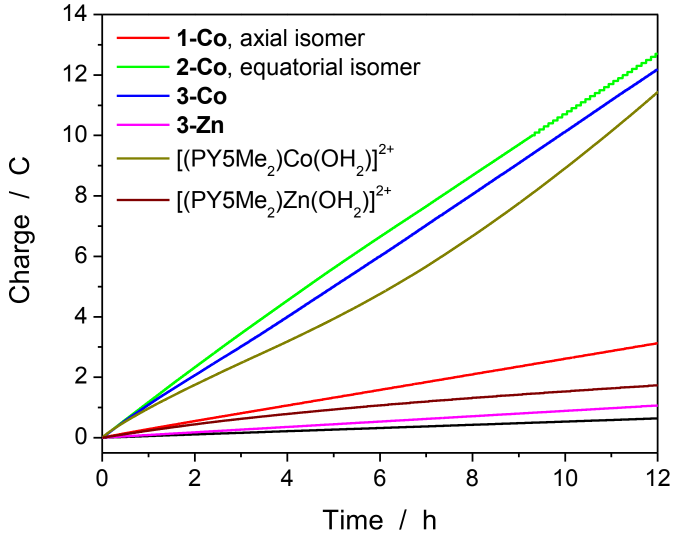

Figure S13. Long-term controlled potential electrolyses in 1M pH 7 KPBS with 10 μM complex at a fixed potential of -1.0 V vs SHE with a Hg pool working electrode. The charge-time profile of **3-Zn** is representative of the series of zinc complexes. The black trace is the background under these conditions.

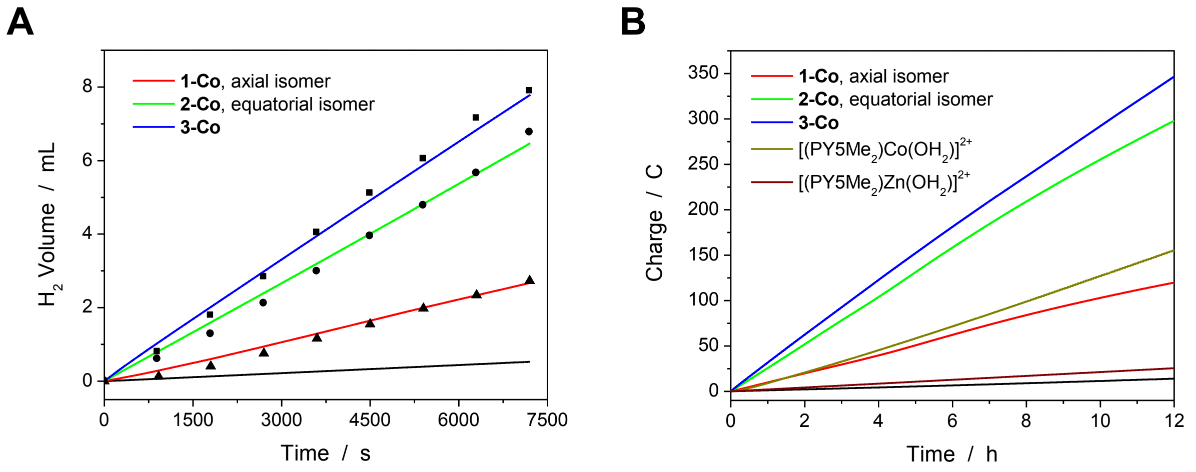

Figure S14. Controlled potential electrolyses in 1M pH 7 KPBS with 10 μM complex at a fixed potential of -1.2 V vs SHE using a Hg pool working electrode. **A.** Black symbols indicate quantified H_2 of headspace samples obtained at various time points over a 2 h period. **B.** Long-term charge-time profiles for 12 h electrolyses. The black traces are the background under these conditions.

1-Co

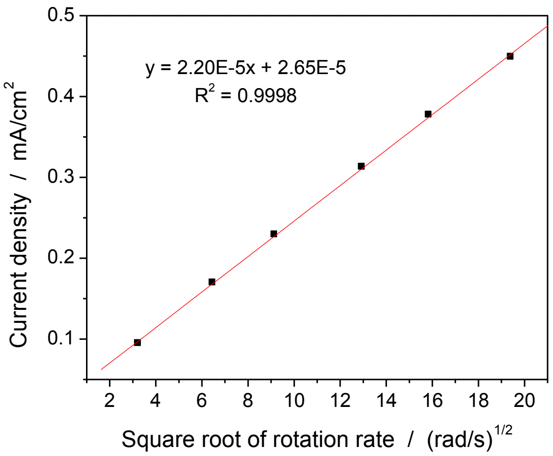

2-Co

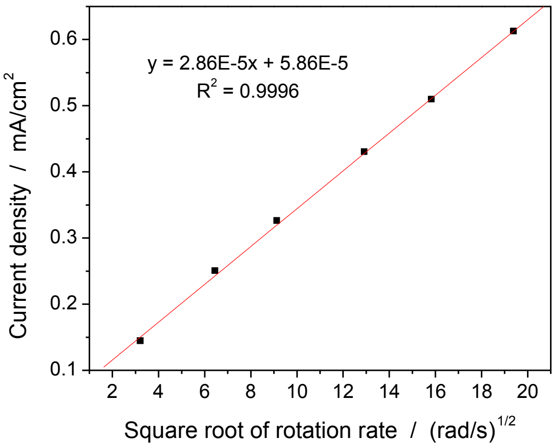

3-Co

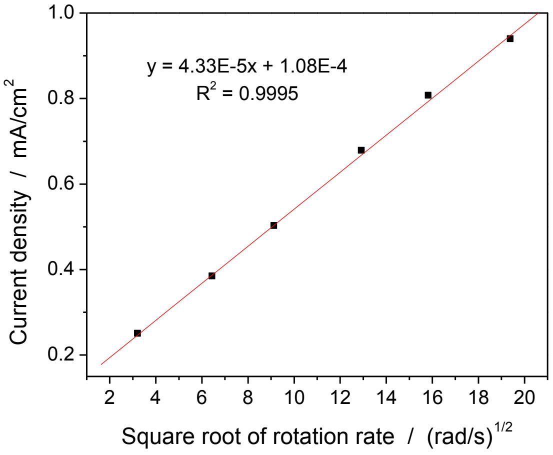

Figure S15. Levich plots are shown for catalysts **1-Co**, **2-Co**, and **3-Co** for current density at -0.85 V vs SHE versus the square root of the rotation rate. Data is shown in Figure 5. *Conditions:* 0.3 mM catalyst, 0.1 M pH 7 KPBS, 0.1 M KNO_3, glassy carbon electrode.

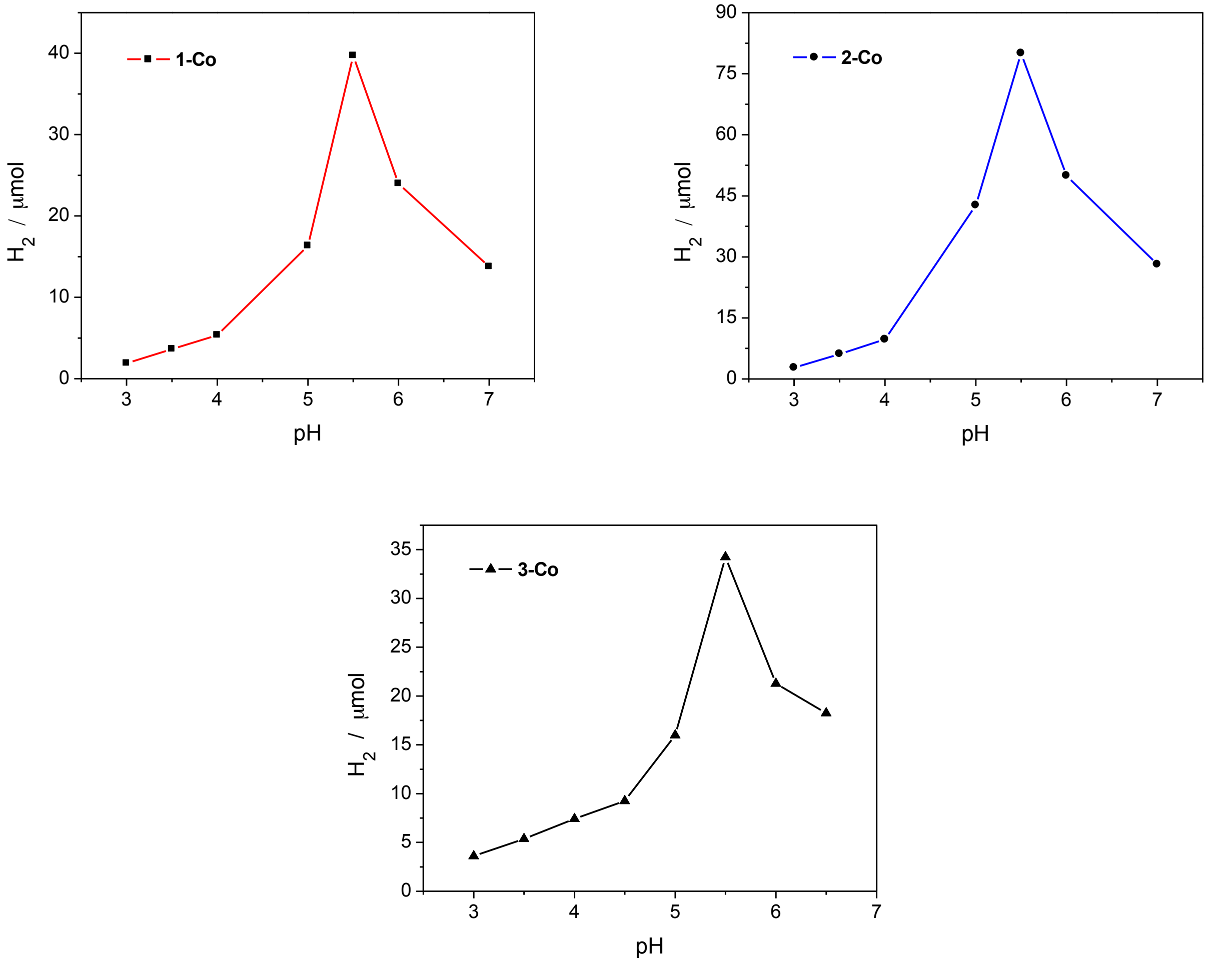

Figure S16. Final accumulated H_2 measured after 18 h of photocatalysis as a function of pH. *Conditions*: 2.0 x 10^{-5} M Co(II) catalyst, 3.3 x 10^{-4} M $[Ru(bpy)_3]^{2+}$ and 0.3 M H_2A/HA^-.

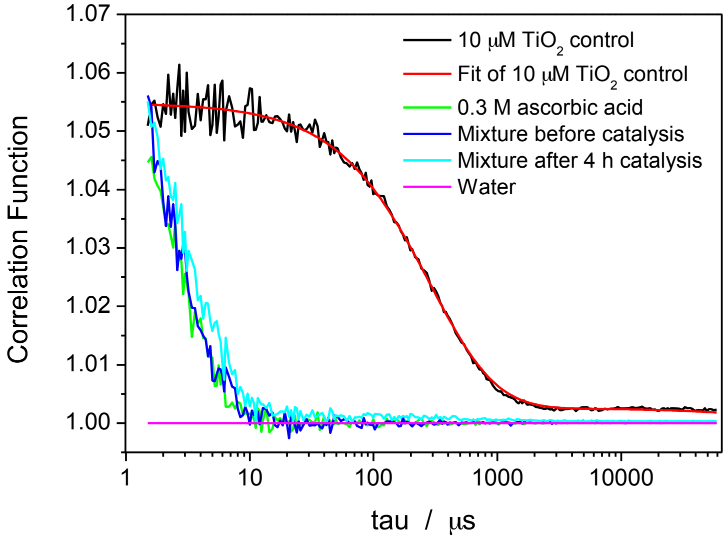

Figure S17. Dynamic Light Scattering (DLS) before and after 4 hours of irradiation under 452 ± 10 nm (540 mW). *Conditions:* 3.3 x 10^{-4} M $[Ru(bpy)_3]^{2+}$, 2 x 10^{-5} M **2-Co**, 0.3 M ascorbic acid at pH 5.5. Degussa p25 titanium dioxide (1.0 x 10^{-5} M) was used as the standard with its autocorrelation fit to detect any particles by light scattering. The absence of nanoparticles in the mixture above 0.5 nm in radius, before and after catalysis, resulted in no light scattering.

Computational Results

All free energies (calculated at 298.15 K and 1 atm) include zero-point-vibrational energy and are corrected using the modified harmonic oscillator approximation proposed by Grimme.[1] This method interpolates between vibrational S_v and rotational S_R approximations to the entropy contribution of low-lying modes. The entropy S for low-frequency modes is given by:

$$S = w(\omega)S_v + [1 - w(\omega)]S_R \tag{1}$$

where the $w(\omega)$ is the Head-Gordon damping function with $\alpha = 4$:

$$w(\omega) = \frac{1}{1 + \left(\omega_0/\omega\right)^{\alpha}} \tag{2}$$

As proposed by Grimme, a cutoff value of $\omega_0 = 100$ cm^{-1} was chosen. In this case, the vibrational entropy for all modes with frequencies lower than 100 cm^{-1} is replaced by a corresponding free-rotor entropy.

Table S1. Experimental and calculated (B3LYP, B3LYP-D2 and *ω*B97X-D) bond lengths (Ångströms) in the gas phase for **1-Co** (S = 3/2) and **1-Zn** (S = 0).

Complexes	Bond Lengths	M-N$_{ax}$	M-O	avg M-N$_{eq}$
	Exp.	2.1050(13)	2.0342(12)	2.1415(13)
**1-Co**	Calc. (B3LYP)	2.109	2.182	2.177
[(*ax*-PY4PZMe$_2$)**Co**(OH$_2$)]$^{2+}$	Calc. (B3LYP-D2)	2.110	2.136	2.157
	Calc. (*ω*B97X-D)	2.103	2.161	2.157
	Exp.	2.147(3)	2.039(3)	2.160(4)
**1-Zn**	Calc. (B3LYP)	2.117	2.240	2.190
[(*ax*-PY4PZMe$_2$)**Zn**(OH$_2$)]$^{2+}$	Calc. (B3LYP-D2)	2.121	2.189	2.170
	Calc. (*ω*B97X-D)	2.108	2.223	2.166

Table S2. Experimental and calculated (B3LYP, B3LYP-D2 and *ω*B97X-D) bond lengths (Ångströms) in the gas phase for **2-Co** (S = 3/2) and **2-Zn** (S = 0).

Complexes	Bond Lengths	M-N$_{ax}$	M-O	avg M-N$_{eq}$
	Exp.	2.099(2)	2.0316(19)	2.127(2)
**2-Co**	Calc. (B3LYP)	2.108	2.193	2.171
[(*eq*-PY4PZMe$_2$)**Co**(OH$_2$)]$^{2+}$	Calc. (B3LYP-D2)	2.108	2.147	2.151
	Calc. (*ω*B97X-D)	2.102	2.172	2.152
	Exp.	2.1368(19)	2.0608(17)	2.150(2)
**2-Zn**	Calc. (B3LYP)	2.112	2.267	2.185
[(*eq*-PY4PZMe$_2$)**Zn**(OH$_2$)]$^{2+}$	Calc. (B3LYP-D2)	2.118	2.208	2.165
	Calc. (*ω*B97X-D)	2.105	2.239	2.162

Table S3. Experimental and calculated (B3LYP, B3LYP-D2 and *ω*B97X-D) bond lengths (Ångströms) in the gas phase for **3-Co** (S = 3/2) and **3-Zn** (S = 0).

Complexes	Bond Lengths	M-N$_{ax}$	M-O	avg M-N$_{eq}$
	Exp.	2.094(3)	2.016(3)	2.113(3)
**3-Co**	Calc. (B3LYP)	2.111	2.189	2.171
[(PY3PZ2Me$_2$)**Co**(OH$_2$)]$^{2+}$	Calc. (B3LYP-D2)	2.111	2.142	2.151
	Calc. (*ω*B97X-D)	2.104	2.167	2.153
	Exp.	2.141(2)	2.0474(18)	2.157(2)
**3-Zn**	Calc. (B3LYP)	2.114	2.254	2.187
[(PY3PZ2Me$_2$)**Zn**(OH$_2$)]$^{2+}$	Calc. (B3LYP-D2)	2.119	2.200	2.166
	Calc. (*ω*B97X-D)	2.105	2.233	2.164

Table S4. Calculated relative energies between the doublet and quartet states for **1'-Co**, **2'-Co** and **3'-Co** using the B3LYP/BS2 functional with and without dispersion corrections in the gas phase and in acetonitrile (SWIG C-PCM approach). The Mulliken spin population is given for cobalt while $\langle S^2 \rangle$ corresponds to the spin contamination.

Complexes	Multiplicity	$\langle S^2 \rangle$	ρ(Co)	ΔG_{B3LYP}	$\Delta G_{B3LYP/C\text{-}PCM}$	$\Delta G_{B3LYP\text{-}D2}$	$\Delta G_{B3LYP\text{-}D2/C\text{-}PCM}$
**1'-Co**	S = 1/2	0.76	0.95	0.0	0.0	0.0	0.0
[(*ax*-PY4PZMe$_2$)**Co**(CH$_3$CN)]$^{2+}$	**S = 3/2**	**3.76**	**2.70**	**-3.7**	**-3.5**	**-2.0**	**-1.8**
**2'-Co**	S = 1/2	0.76	0.95	0.0	0.0	0.0	0.0
[(*eq*-PY4PZMe$_2$)**Co**(CH$_3$CN)]$^{2+}$	**S = 3/2**	**3.76**	**2.70**	**-3.5**	**-3.5**	**-1.8**	**-1.9**
**3'-Co**	S = 1/2	0.76	0.95	0.0	0.0	0.0	0.0
[(PY3PZ2Me$_2$)**Co**(CH$_3$CN)]$^{2+}$	**S = 3/2**	**3.76**	**2.70**	**-3.5**	**-3.5**	**-1.7**	**-1.8**

Table S5. Calculated relative energies between the singlet and triplet states for the five- and six-coordinate species of **1'-Co+e$^-$**, **2'-Co+e$^-$** and **3'-Co+e$^-$** using the B3LYP/BS2 functional with and without dispersion corrections in the gas phase and in acetonitrile (SWIG C-PCM approach). The Mulliken spin population is given for cobalt while $\langle S^2 \rangle$ corresponds to the spin contamination.

Complexes	Multiplicity	$\langle S^2 \rangle$	ρ(Co)	ΔG_{B3LYP}	$\Delta G_{B3LYP/C\text{-}PCM}$	$\Delta G_{B3LYP\text{-}D2}$	$\Delta G_{B3LYP\text{-}D2/C\text{-}PCM}$
**1'-Co+e$^-$**	S = 0	1.08	0.33	0.0	0.0	0.0	0.0
[(*ax*-PY4PZMe$_2$)**Co**(CH$_3$CN)]$^{1+}$	**S = 1**	**2.21**	**2.14**	**-18.0**	**-16.9**	**-17.7**	**-16.6**
**1'-Co+e$^-$**	S = 0	0.89	0.03	+1.3	-0.9	+7.9	+5.7
[(*ax*-PY4PZMe$_2$)**Co**]$^{1+}$	S = 1	2.14	2.08	-12.8	-15.9	-5.5	-8.6
**2'-Co+e$^-$**	S = 0	1.01	0.08	0.0	0.0	0.0	0.0
[(*eq*-PY4PZMe$_2$)**Co**(CH$_3$CN)]$^{1+}$	**S = 1**	**2.14**	**2.07**	**-16.5**	**-16.5**	**-16.5**	**-16.4**
**2'-Co+e$^-$**	S = 0	0.89	0.16	+1.8	-2.3	+8.2	+4.1
[(*eq*-PY4PZMe$_2$)**Co**]$^{1+}$	S = 1	2.12	2.06	-11.6	-16.0	-4.5	-9.0
**3'-Co+e$^-$**	S = 0	1.01	0.05	0.0	0.0	0.0	0.0
[(PY3PZ2Me$_2$)**Co**(CH$_3$CN)]$^{1+}$	**S = 1**	**2.16**	**2.10**	**-16.5**	**-16.6**	**-16.4**	**-16.6**
**3'-Co+e$^-$**	S = 0	0.87	0.15	+2.3	-2.0	+8.7	+4.3
[(PY3PZ2Me$_2$)**Co**]$^{1+}$	S = 1	2.12	2.06	-11.0	-15.8	-4.0	-8.7

Table S6. Calculated relative energies between the doublet and quartet states for the five- and six-coordinate species of **1'-Co+2e**$^-$, **2'-Co+2e**$^-$ and **3'-Co+2e**$^-$ using the B3LYP/BS2 functional with and without dispersion corrections in the gas phase and in acetonitrile (SWIG C-PCM approach). The Mulliken spin population is given for cobalt while $<S^2>$ corresponds to the spin contamination.

Complexes	Multiplicity	$<S^2>$	ρ(Co)	ΔG_{B3LYP}	$\Delta G_{B3LYP/C\text{-}PCM}$	$\Delta G_{B3LYP\text{-}D2}$	$\Delta G_{B3LYP\text{-}D2/C\text{-}PCM}$
**1'-Co+2e**$^-$	S = 1/2	1.83	2.00	0.0	0.0	0.0	0.0
[(*ax*-PY4PZMe$_2$)**Co**(CH$_3$CN)]^0	S = 3/2	3.86	2.08	-0.8	-0.9	-1.0	-1.1
**1'-Co+2e**$^-$	S = 1/2	1.94	2.09	+0.7	-0.1	+7.4	+6.6
[(*ax*-PY4PZMe$_2$)**Co**]^0	S = 3/2	3.86	2.08	-0.6	-2.0	+6.1	+4.7
**2'-Co+2e**$^-$	S = 1/2	1.94	2.10	0.0	0.0	0.0	0.0
[(*eq*-PY4PZMe$_2$)**Co**(CH$_3$CN)]^0	S = 3/2	3.87	2.09	-0.2	0.0	-0.2	0.0
**2'-Co+2e**$^-$	S = 1/2	2.00	2.18	-2.1	-3.2	+5.3	+4.2
[(*eq*-PY4PZMe$_2$)**Co**]^0	S = 3/2	3.94	2.16	-1.5	-3.5	+5.6	+3.5
**3'-Co+2e**$^-$	S = 1/2	2.00	2.15	0.0	0.0	0.0	0.0
[(PY3PZ2Me$_2$)**Co**(CH$_3$CN)]^0	S = 3/2	3.90	2.11	-0.4	-0.9	-0.5	-1.0
**3'-Co+2e**$^-$	S = 1/2	1.98	2.15	-0.9	-1.9	+6.4	+5.5
[(PY3PZ2Me$_2$)**Co**]^0	S = 3/2	3.94	2.16	-1.2	-3.3	+5.9	+3.8

DFT calculations suggest that the cobalt(II) complexes **1'-Co, 2'-Co and 3'-Co** have a quartet ground-state (Table S4). The first reduction yields a Co^{1+} species (Figures S21-26) with a triplet ground-state (Table S5). Dissociation of the acetonitrile solvent molecule has also been considered after the first reduction. Calculations suggest that formation of a five-coordinate species is higher in energy than the six-coordinate complexes in solution ($\Delta G_{B3LYP/C\text{-}PCM}$ ~ +1.0 kcal/mol; $\Delta G_{B3LYP\text{-}D2/C\text{-}PCM}$ ~ +8.0 kcal/mol). The second reduction is computed to be ligand-centered (see below) for both doublet and quartet states in which five- and six-coordinate species are competitive (Table S6). For instance, DFT calculations performed using the B3LYP functional (S = 3/2) suggest that formation of the five-coordinate complex are lower in energy ($\Delta G_{B3LYP/C\text{-}PCM}$ = -2.0 kcal/mol for **1'-Co+2e**$^-$; $\Delta G_{B3LYP/C\text{-}PCM}$ = -3.5 kcal/mol for **2'-Co+2e**$^-$; $\Delta G_{B3LYP/C\text{-}PCM}$ = -3.3 kcal/mol for **3'-Co+2e**$^-$). However, calculations using the B3LYP-D2 functional (S = 3/2) show that the six-coordinate complexes are slightly higher in energy ($\Delta G_{B3LYP\text{-}D2/C\text{-}PCM}$ = +4.7 kcal/mol for **1'-Co+2e**$^-$; $\Delta G_{B3LYP\text{-}D2/C\text{-}PCM}$ = +3.5 kcal/mol for **2'-Co+2e**$^-$; $\Delta G_{B3LYP\text{-}D2/C\text{-}PCM}$ = +3.8 kcal/mol for **3'-Co+2e**$^-$). These observations suggest that a competition between five- and six-coordinate species may play a role in catalysis.

Table S7. Calculated redox potentials (V *vs.* Fc^{+}/Fc) for the Co^{2+} complexes (S = 3/2) and their one- (S = 1) and two-electron reduced species (S =1/2 and 3/2) in solution (acetonitrile *via* C-PCM approach). In the case of the second reduction, the redox potentials were calculated for the five-coordinate species after dissociation of the acetonitrile molecule. Redox potentials for the six-coordinate species are given in the main text.

Complex	$E_{p1,c}$ (S = 1)		$E_{p2,c}$ (S = 1/2 and 3/2)		
	exptl	calcd	exptl	calcd	calcd
**1'-Co,** [(*ax*-PY4PZMe$_2$)Co(CH_3CN)]$^{2+}$	-1.22	-1.28	-1.40	-1.44[b]	-1.43[b]
**2'-Co,** [(*eq*-PY4PZMe$_2$)Co(CH_3CN)]$^{2+}$	-1.30	-1.34	-1.42	-1.36[b]	-1.41[b]
**3'-Co,** [(PY3PZ2Me$_2$)Co(CH_3CN)]$^{2+}$	-1.18	-1.18[a]	-1.25	-1.25[ab]	-1.25[ab]

[a]This redox potential was used as reference in the isodesmic reactions, so it agrees by construction, and all other reduction potentials are calculated relative to this value.

[b]Dissociation of the acetonitrile molecule.

Edmiston-Ruedenberg localized orbitals for 1'-Co, 2'-Co and 3'-Co.

In order to gain information on the oxidation state of the cobalt complexes **1'-Co**, **2'-Co** and **3'-Co**, localized orbital bonding analysis (LOBA) using the Edmiston-Ruedenberg localized orbitals and the Löwdin population analysis were employed (Figures S18-20). LOBA calculations suggest that **1'-Co**, **2'-Co** and **3'-Co** have an oxidation state of +2.

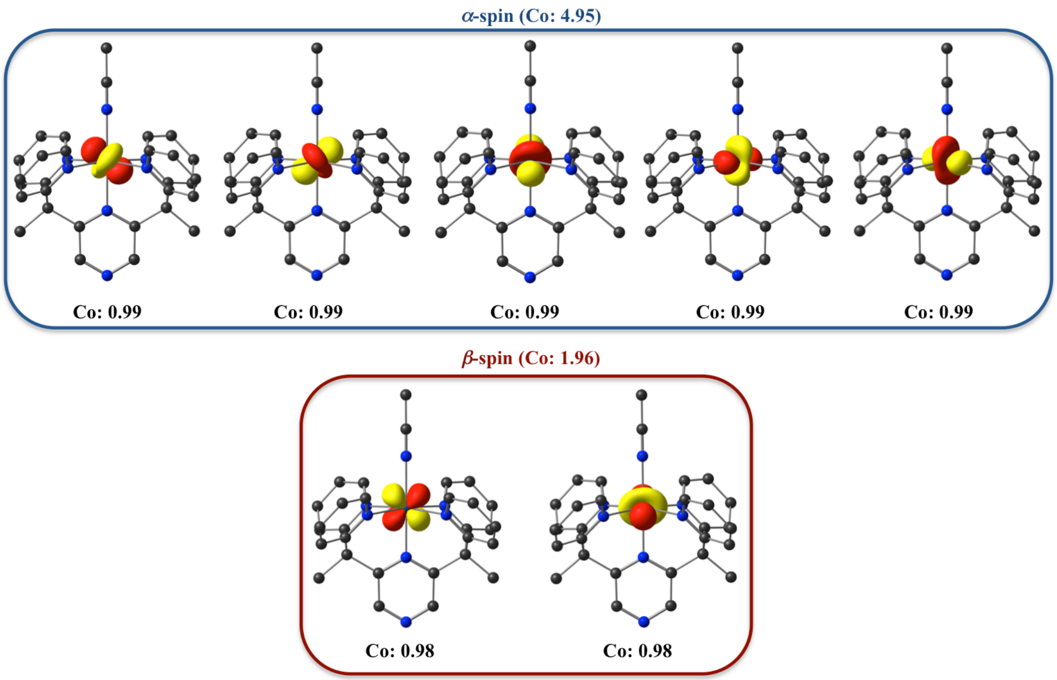

Figure S18. Isosurface (0.07 au) plots of the Edmiston-Ruedenberg localized orbitals for **1'-Co** (Co^{2+}, S = 3/2) using the B3LYP functional. The Löwdin population analyses are given for cobalt.

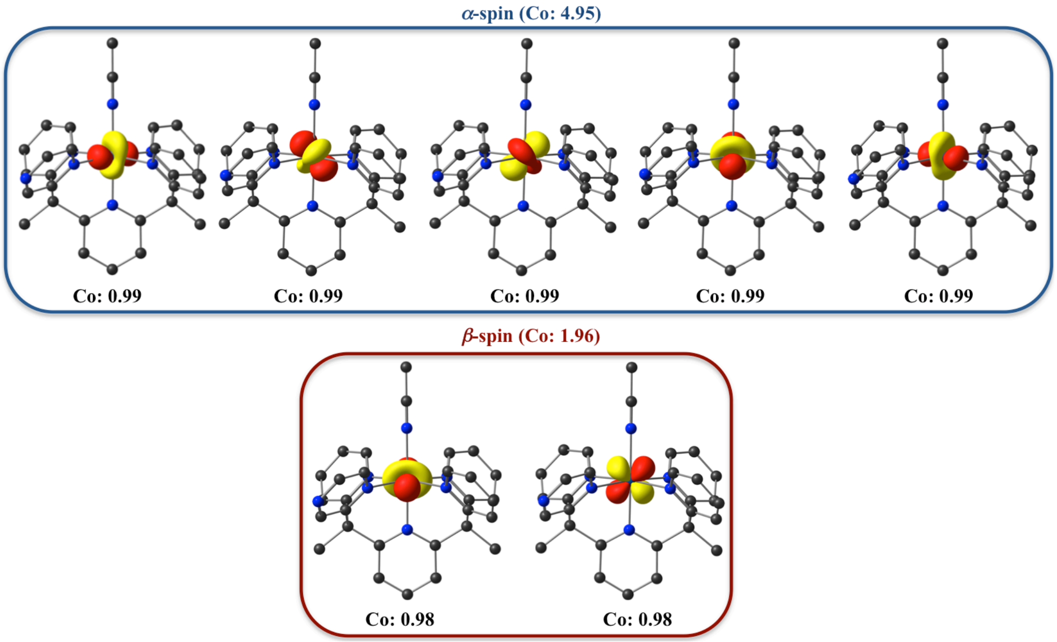

Figure S19. Isosurface (0.07 au) plots of the Edmiston-Ruedenberg localized orbitals for **2'-Co** (Co^{2+}, S = 3/2) using the B3LYP functional. The Löwdin population analyses are given for cobalt.

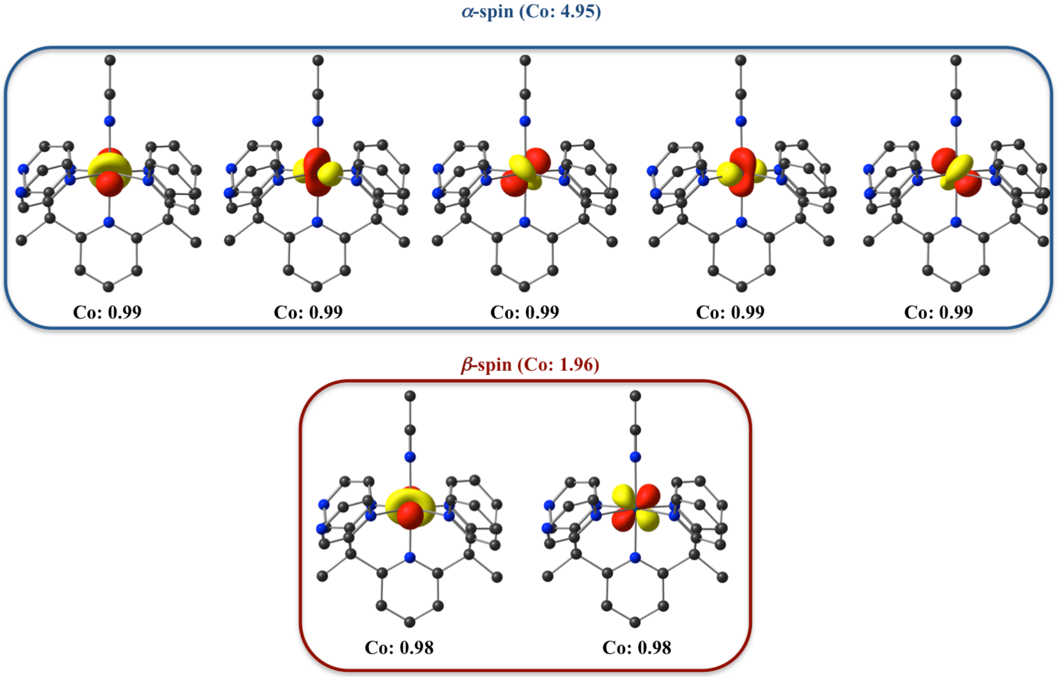

Figure S20. Isosurface (0.07 au) plots of the Edmiston-Ruedenberg localized orbitals for **3'-Co** (Co^{2+}, S = 3/2) using the B3LYP functional. The Löwdin population analyses are given for cobalt.

Edmiston-Ruedenberg localized orbitals and canonical molecular orbitals for 1'-Co+e$^-$, 2'-Co+e$^-$ and 3'-Co+e$^-$ obtained from 1'-Co, 2'-Co and 3'-Co after one-electron reduction.

Edmiston-Ruedenberg localized orbitals (Figures S21-23) and canonical molecular orbitals (Figures S24-26) have been computed for the one-electron reduced species (**1'-Co+e**$^-$, **2'-Co+e**$^-$ and **3'-Co+e**$^-$ obtained from **1'-Co**, **2'-Co** and **3'-Co**). DFT calculations (S = 1) suggest that the first reduction is mainly metal-centered.

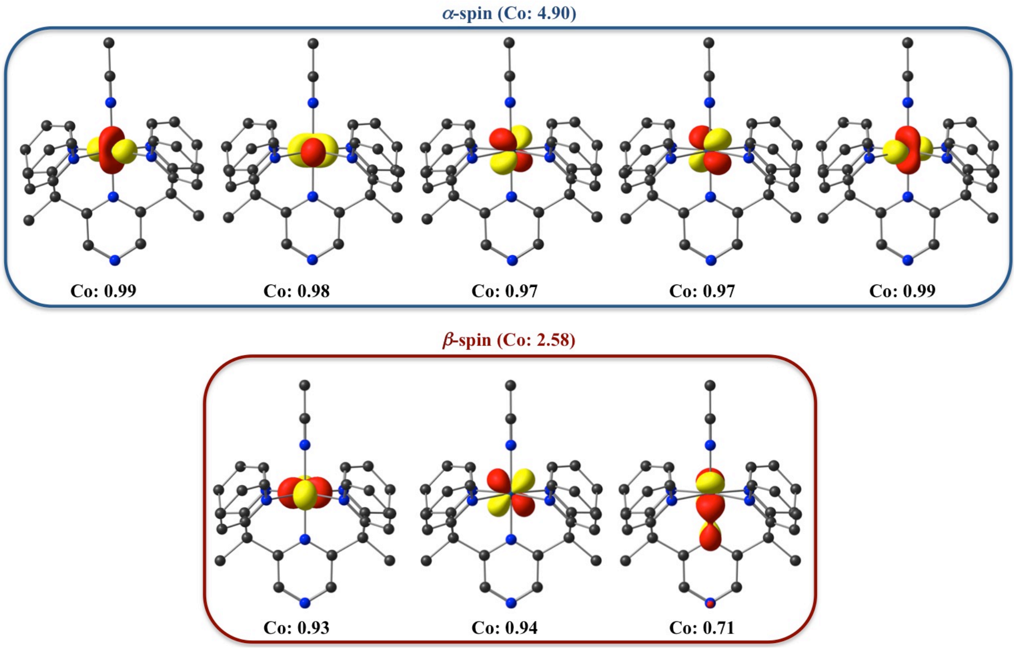

Figure S21. Isosurface (0.07 au) plots of the Edmiston-Ruedenberg localized orbitals for **1'-Co+e**$^-$ (S = 1) using the B3LYP functional. The Löwdin population analyses are given for cobalt.

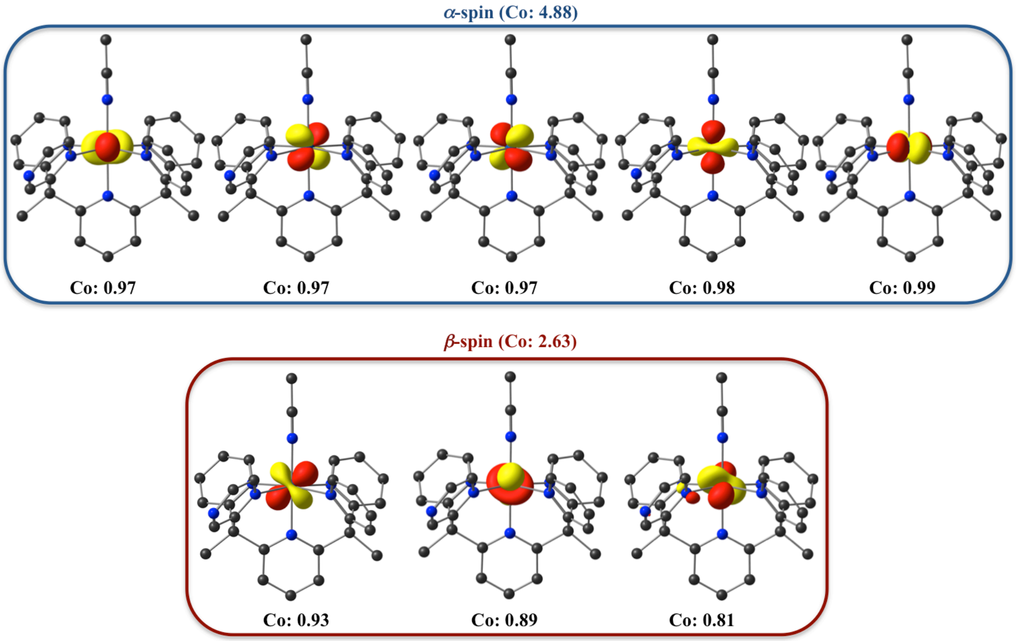

Figure S22. Isosurface (0.07 au) plots of the Edmiston-Ruedenberg localized orbitals for **2'-Co+e**$^-$ (S = 1) using the B3LYP functional. The Löwdin population analyses are given for cobalt.

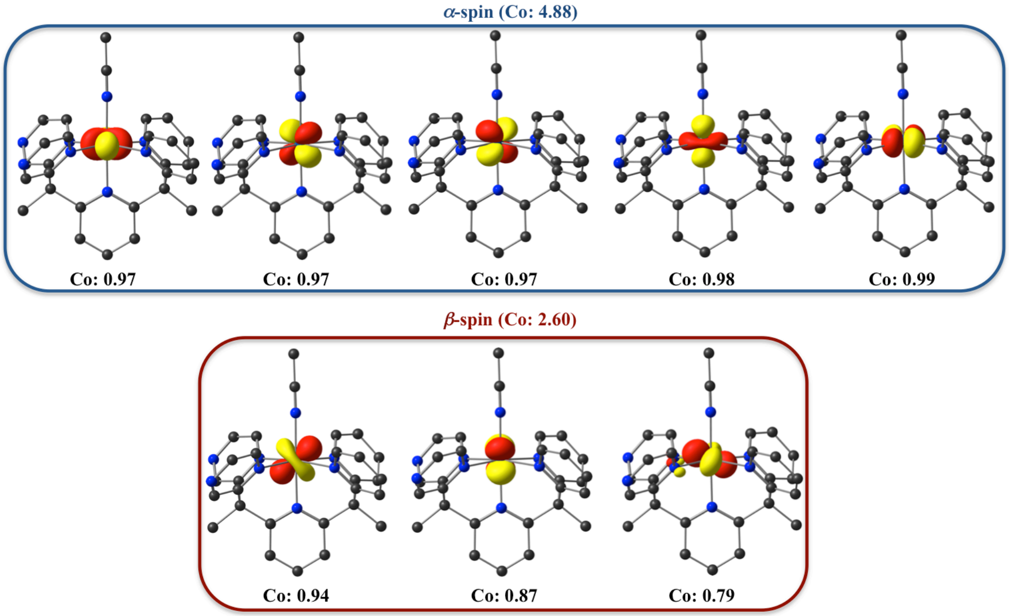

Figure S23. Isosurface (0.07 au) plots of the Edmiston-Ruedenberg localized orbitals for **3'-Co+e**$^-$ (S = 1) using the B3LYP functional. The Löwdin population analyses are given for cobalt.

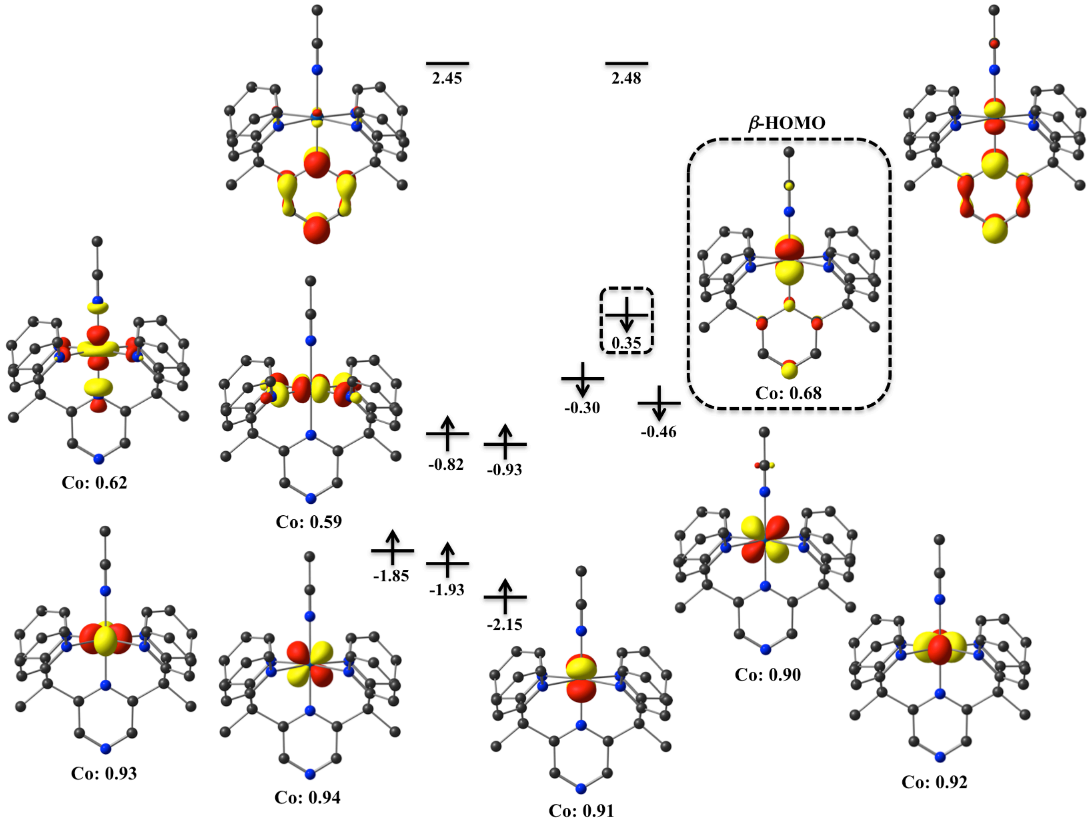

Figure S24. Isosurface (0.07 au) plots of the canonical molecular orbitals for **1'-Co+e$^-$** (S = 1) using the B3LYP functional. The Löwdin population analyses are given for cobalt. All energies (in eV) are relative to the HOMO (highest occupied molecular orbital) of **3'-Co+e$^-$** (S = 1).

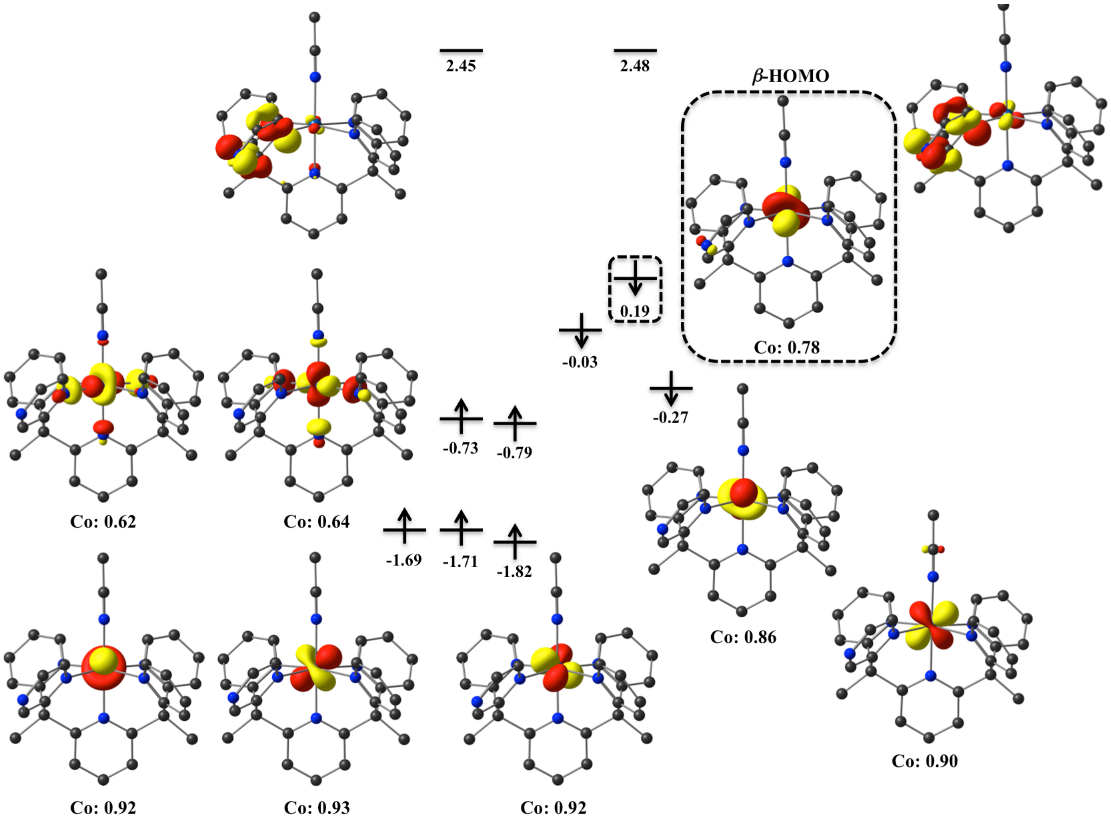

Figure S25. Isosurface (0.07 au) plots of the canonical molecular orbitals for **2'-Co+e**$^-$ (S = 1) using the B3LYP functional. The Löwdin population analyses are given for cobalt. All energies (in eV) are relative to the HOMO (highest occupied molecular orbital) of **3'-Co+e**$^-$ (S = 1).

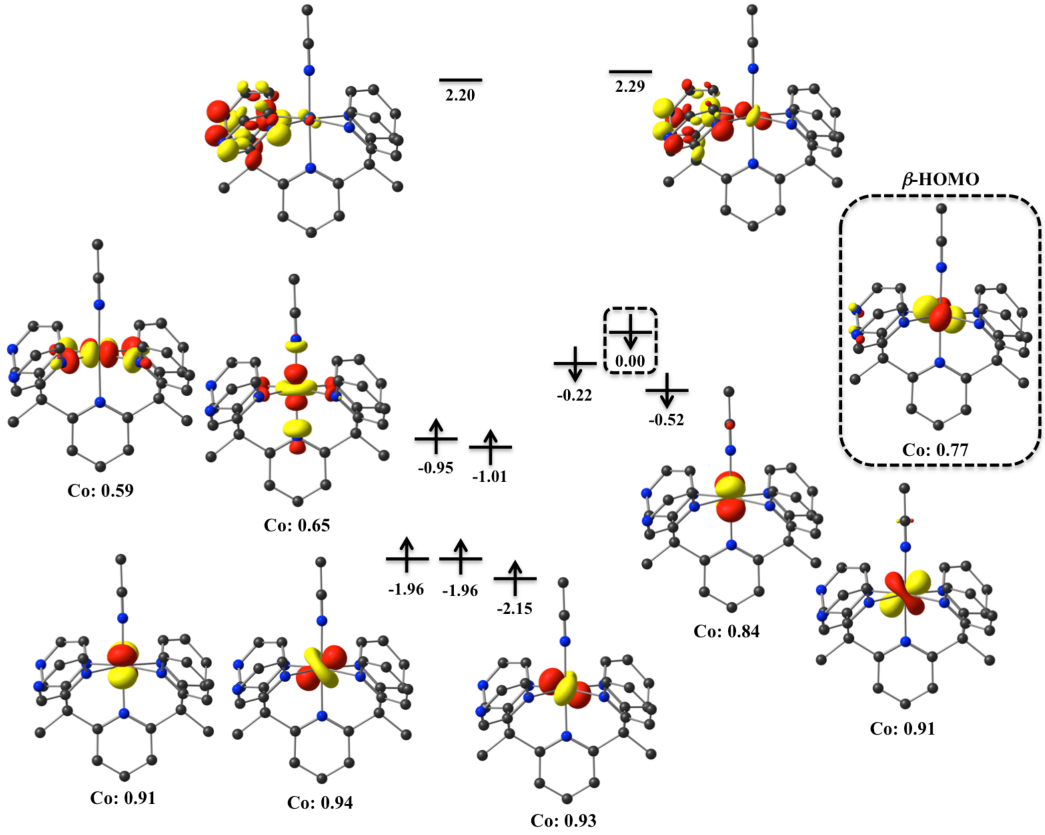

Figure S26. Isosurface (0.07 au) plots of the canonical molecular orbitals for **3'-Co+e**$^-$ (S = 1) using the B3LYP functional. The Löwdin population analyses are given for cobalt. All energies (in eV) are relative to the HOMO (highest occupied molecular orbital).

Edmiston-Ruedenberg localized orbitals and canonical molecular orbitals for the six-coordinate species 1'-Co+2e$^-$, 2'-Co+2e$^-$ and 3'-Co+2e$^-$ obtained after two-electron reduction.

Edmiston-Ruedenberg localized orbitals (Figures S27-32) and canonical molecular orbitals (Figures S33-38) have been computed for the two-electron reduced species to give **1'-Co+2e**$^-$, **2'-Co+2e**$^-$ and **3'-Co+2e**$^-$. As shown in Table S6, the doublet and quartet states are degenerate, however, the second reduction is computed to be ligand-centered in both states for all three species. Interestingly, the LUMOs (lowest unoccupied molecular orbitals) are computed to be ligand-centered. This suggests that the third reduction will be ligand-centered. Similar conclusions have been observed for the five-coordinate species (see below).

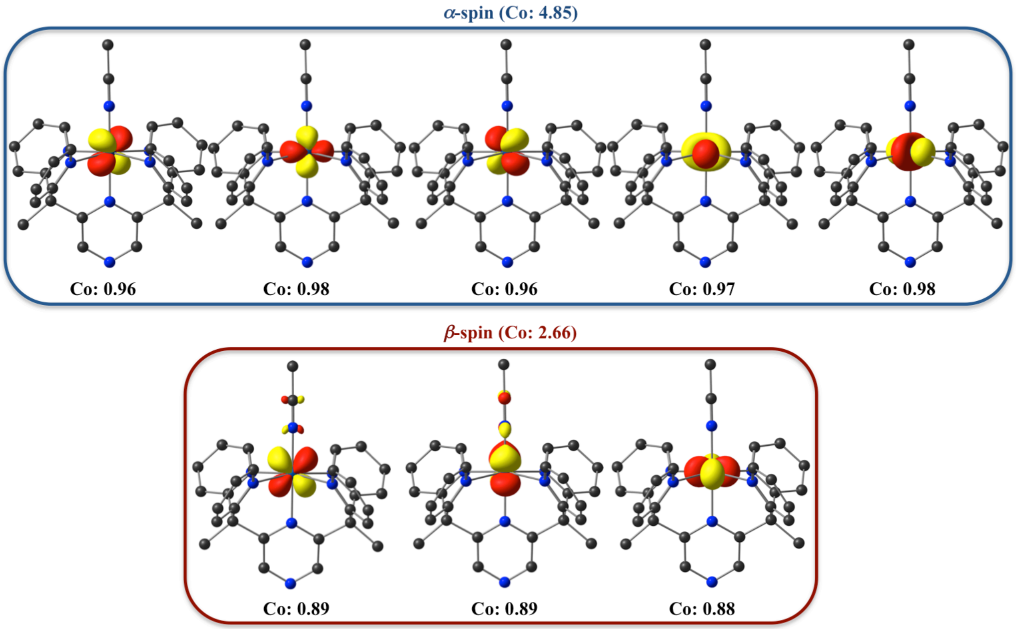

Figure S27. Isosurface (0.07 au) plots of the Edmiston-Ruedenberg localized orbitals for **1'-Co+2e$^-$** (S = 1/2) using the B3LYP functional. The Löwdin population analyses are given for cobalt.

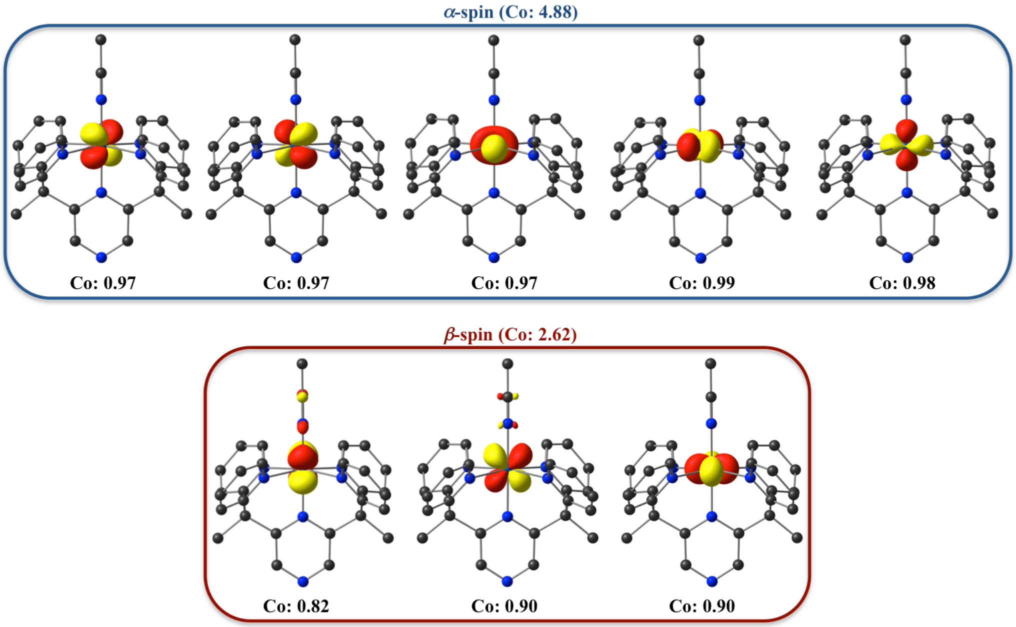

Figure S28. Isosurface (0.07 au) plots of the Edmiston-Ruedenberg localized orbitals for **1'-Co+2e$^-$** (S = 3/2) using the B3LYP functional. The Löwdin population analyses are given for cobalt.

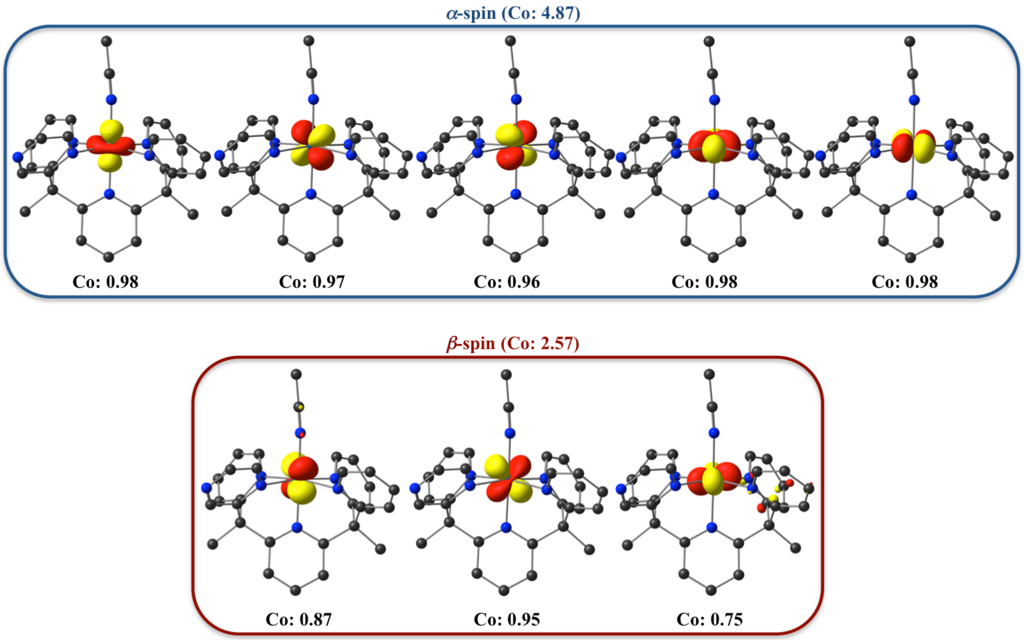

Figure S29. Isosurface (0.07 au) plots of the Edmiston-Ruedenberg localized orbitals for **2'-Co+2e**$^-$ (S = 1/2) using the B3LYP functional. The Löwdin population analyses are given for cobalt.

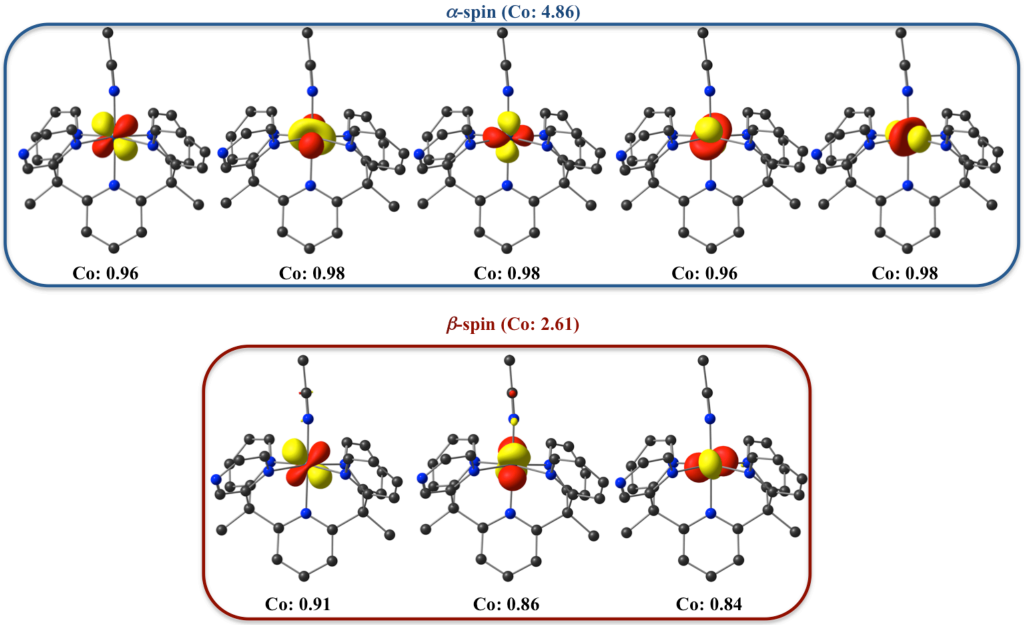

Figure S30. Isosurface (0.07 au) plots of the Edmiston-Ruedenberg localized orbitals for **2'-Co+2e**$^-$ (S = 3/2) using the B3LYP functional. The Löwdin population analyses are given for cobalt.

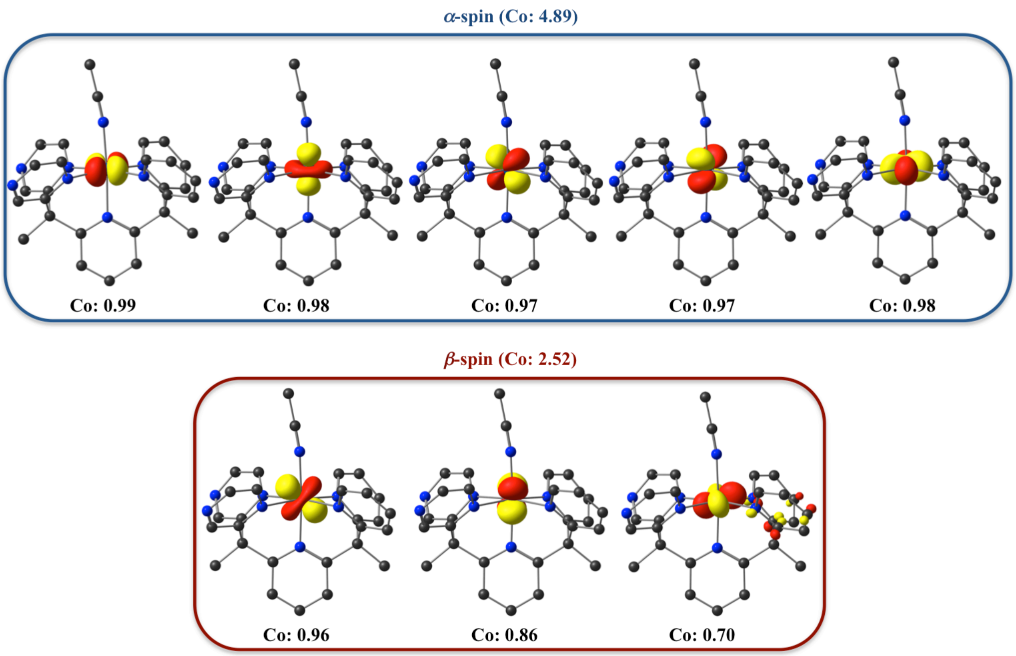

Figure S31. Isosurface (0.07 au) plots of the Edmiston-Ruedenberg localized orbitals for **3'-Co+2e**$^-$ (S = 1/2) using the B3LYP functional. The Löwdin population analyses are given for cobalt.

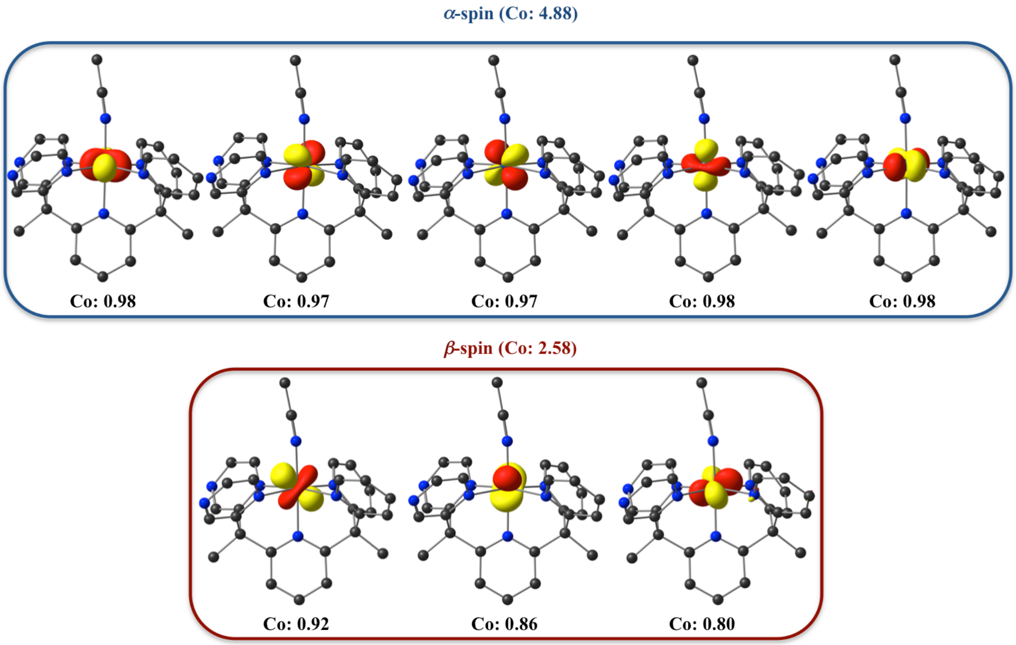

Figure S32. Isosurface (0.07 au) plots of the Edmiston-Ruedenberg localized orbitals for **3'-Co+2e**$^-$ (S = 3/2) using the B3LYP functional. The Löwdin population analyses are given for cobalt.

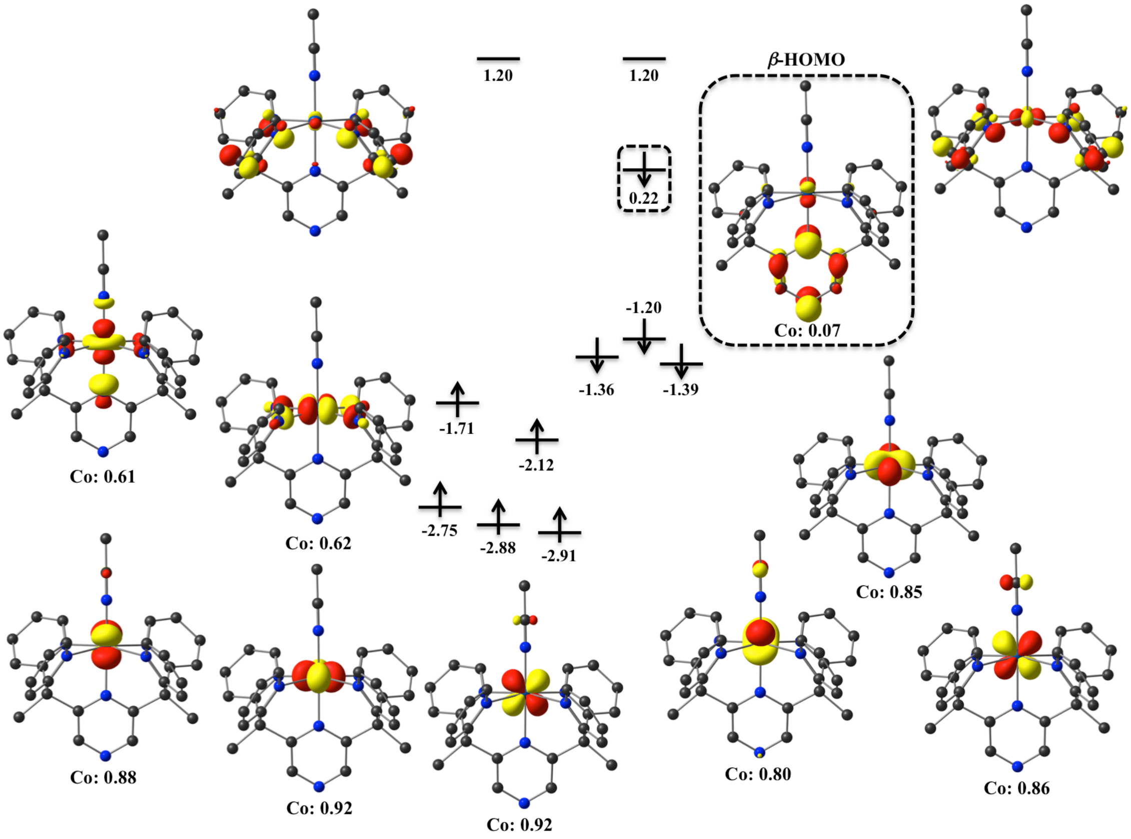

Figure S33. Isosurface (0.07 au) plots of the canonical molecular orbitals for **1'-Co+2e$^-$** (S = 1/2) using the B3LYP functional. The Löwdin population analyses are given for cobalt. All energies (in eV) are relative to the HOMO (highest occupied molecular orbital) of the six-coordinate species **3'-Co+2e$^-$** (S = 1/2).

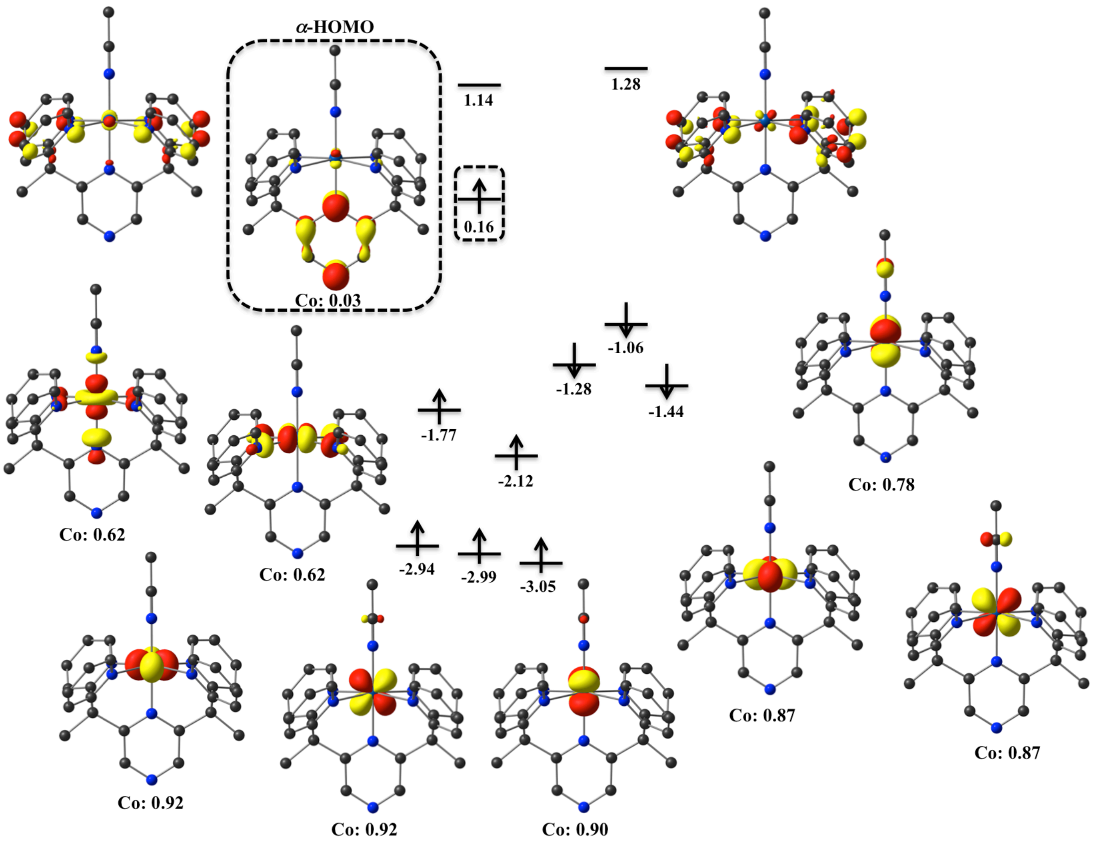

Figure S34. Isosurface (0.07 au) plots of the canonical molecular orbitals for **1'-Co+2e**$^-$ (S = 3/2) using the B3LYP functional. The Löwdin population analyses are given for cobalt. All energies (in eV) are relative to the HOMO (highest occupied molecular orbital) of the six-coordinate species **3'-Co+2e**$^-$ (S = 1/2).

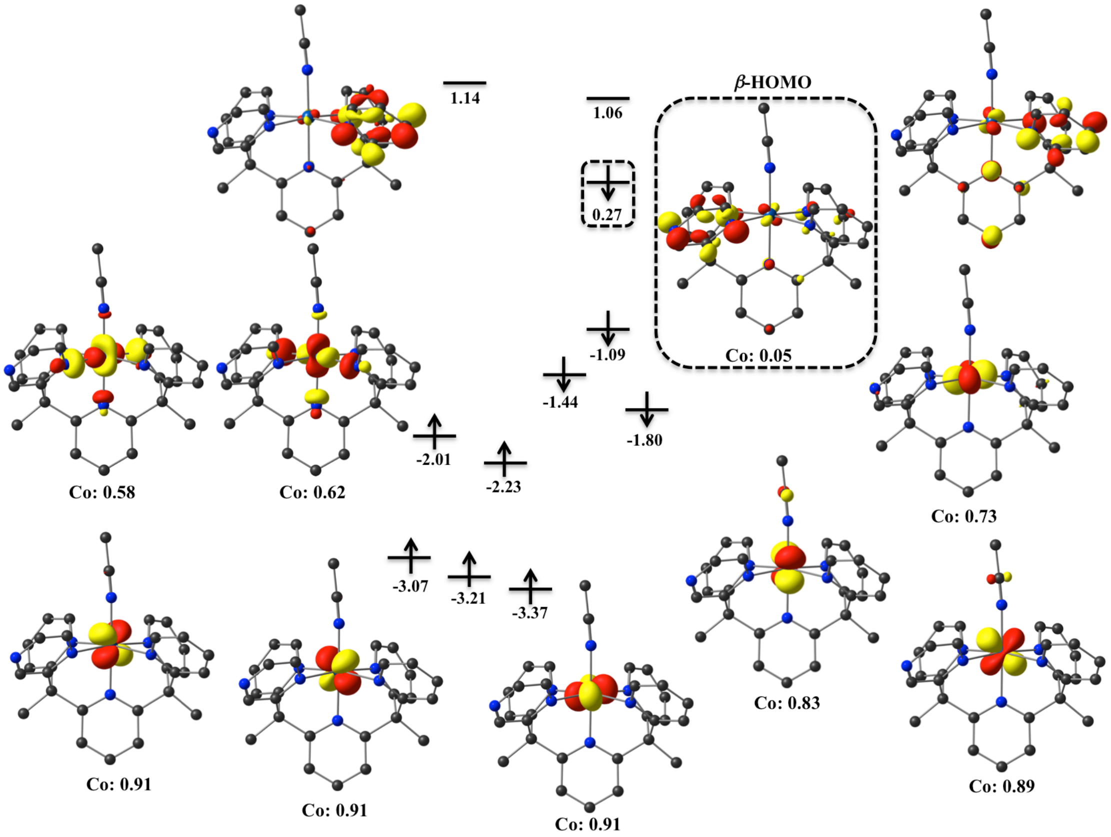

Figure S35. Isosurface (0.07 au) plots of the canonical molecular orbitals for **2'-Co+2e**$^{-}$ (S = 1/2) using the B3LYP functional. The Löwdin population analyses are given for cobalt. All energies (in eV) are relative to the HOMO (highest occupied molecular orbital) of the six-coordinate species **3'-Co+2e**$^{-}$ (S = 1/2).

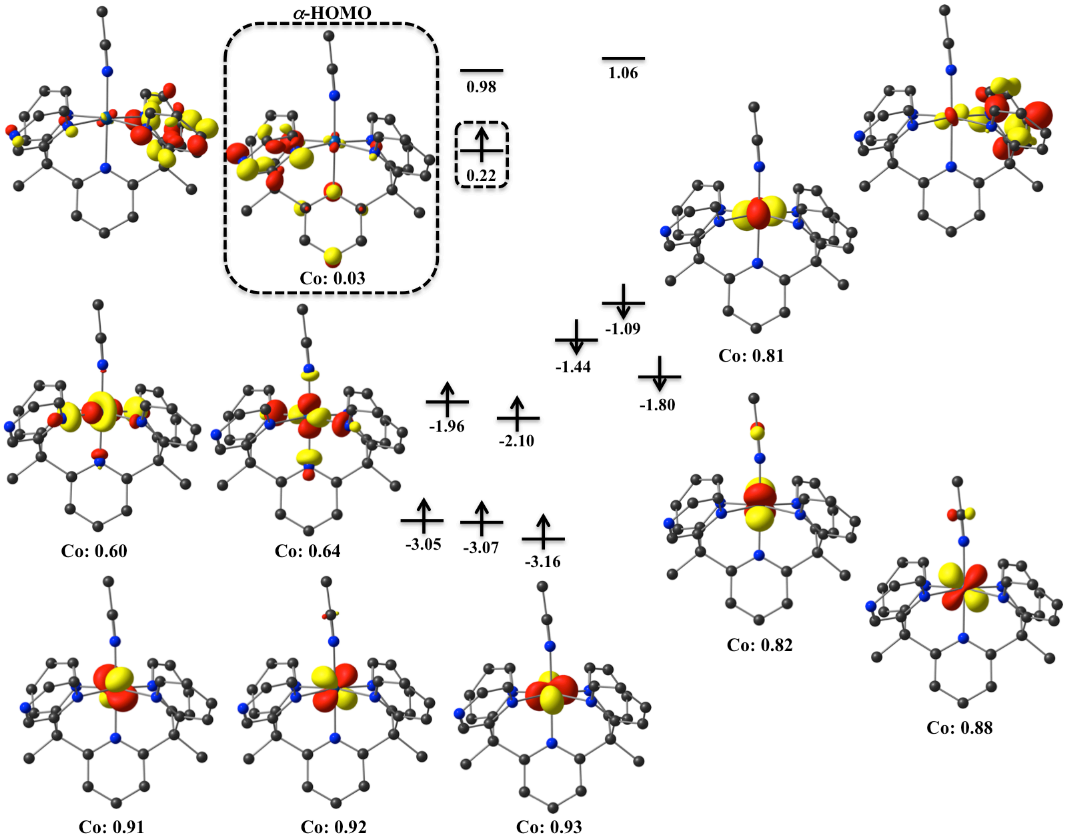

Figure S36. Isosurface (0.07 au) plots of the canonical molecular orbitals for **2'-Co+2e$^-$** (S = 3/2) using the B3LYP functional. The Löwdin population analyses are given for cobalt. All energies (in eV) are relative to the HOMO (highest occupied molecular orbital) of the six-coordinate species **3'-Co+2e$^-$** (S = 1/2).

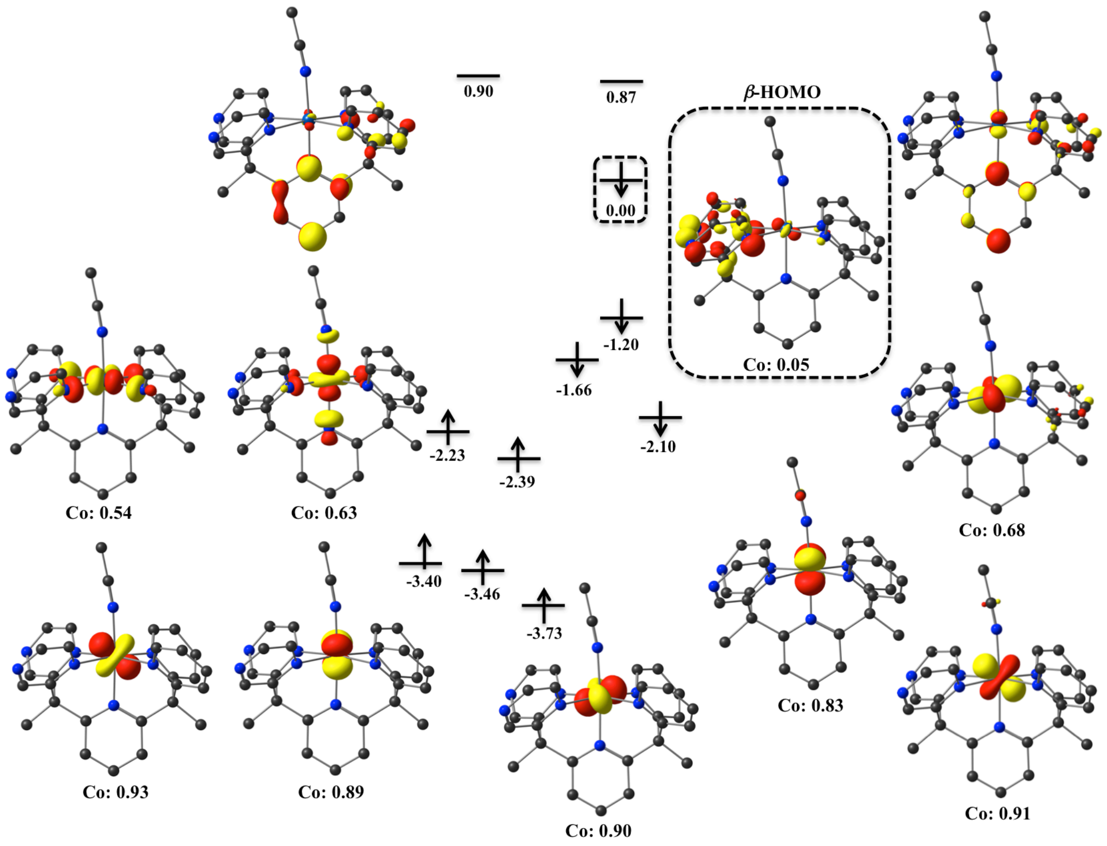

Figure S37. Isosurface (0.07 au) plots of the canonical molecular orbitals for **3'-Co+2e$^-$** (S = 1/2) using the B3LYP functional. The Löwdin population analyses are given for cobalt. All energies (in eV) are relative to the HOMO (highest occupied molecular orbital).

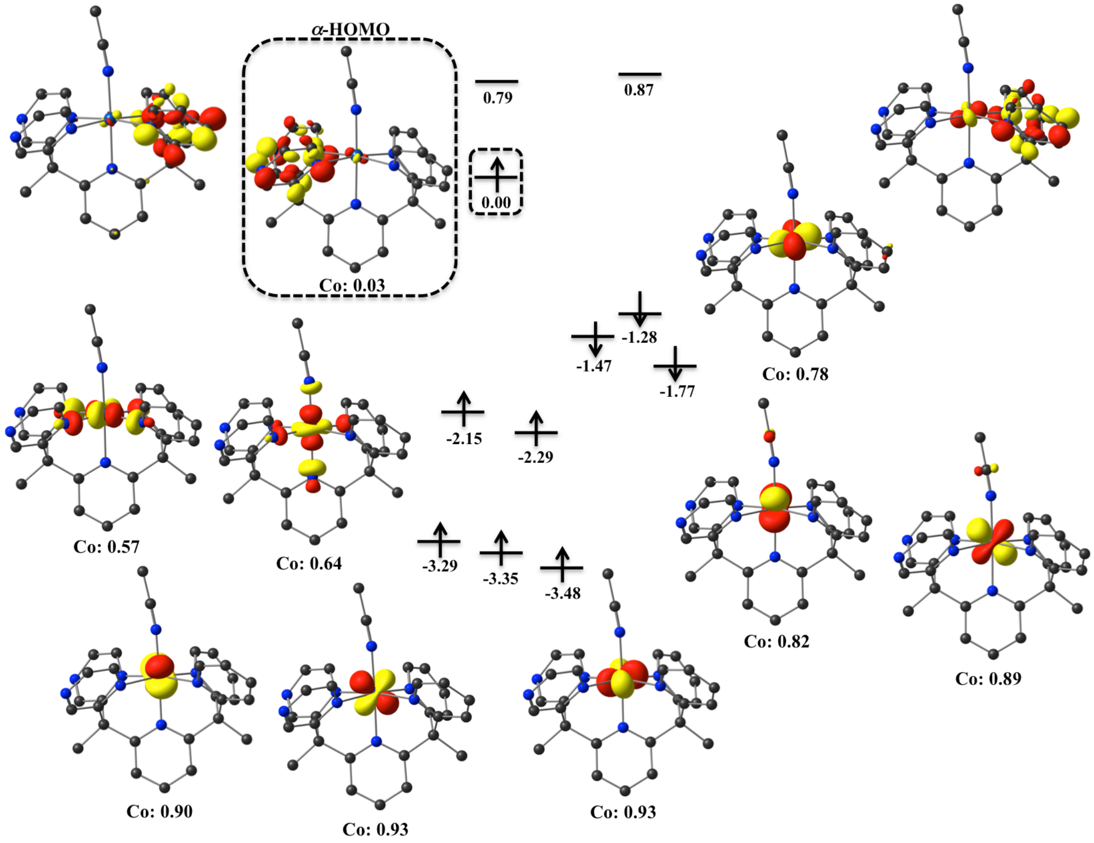

Figure S38. Isosurface (0.07 au) plots of the canonical molecular orbitals for **3'-Co+2e$^-$** (S = 3/2) using the B3LYP functional. The Löwdin population analyses are given for cobalt. All energies (in eV) are relative to the HOMO (highest occupied molecular orbital) of the six-coordinate species **3'-Co+2e$^-$** (S = 1/2).

Edmiston-Ruedenberg localized orbitals and canonical molecular orbitals for the five-coordinate species 1'-Co+2e$^-$, 2'-Co+2e$^-$ and 3'-Co+2e$^-$ obtained after two-electron reduction and dissociation of the apical acetonitrile molecule.

Edmiston-Ruedenberg localized orbitals (Figures S39-44) and canonical molecular orbitals (Figures S45-50) have been computed for the two-electron reduced species after dissociation of the solvent molecule. Again, in this case the doublet and quartet states are degenerate and the second reduction is computed to be ligand-centered. As shown for the six-coordinate species, the LUMOs (lowest unoccupied molecular orbitals) are also calculated to be ligand-based.

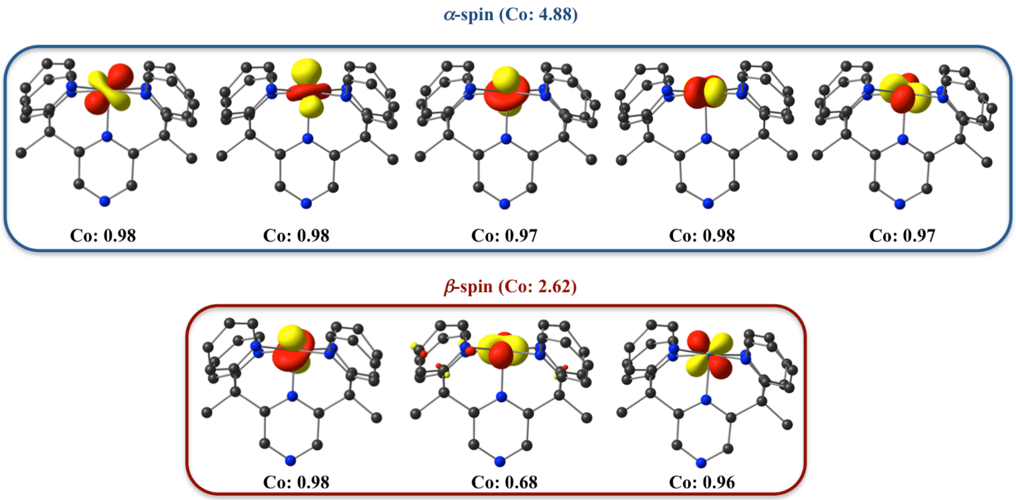

Figure S39. Isosurface (0.07 au) plots of the Edmiston-Ruedenberg localized orbitals for **1'-Co+2e**$^{-}$ (S = 1/2) after dissociation of the acetonitrile molecule using the B3LYP functional. The Löwdin population analyses are given for cobalt.

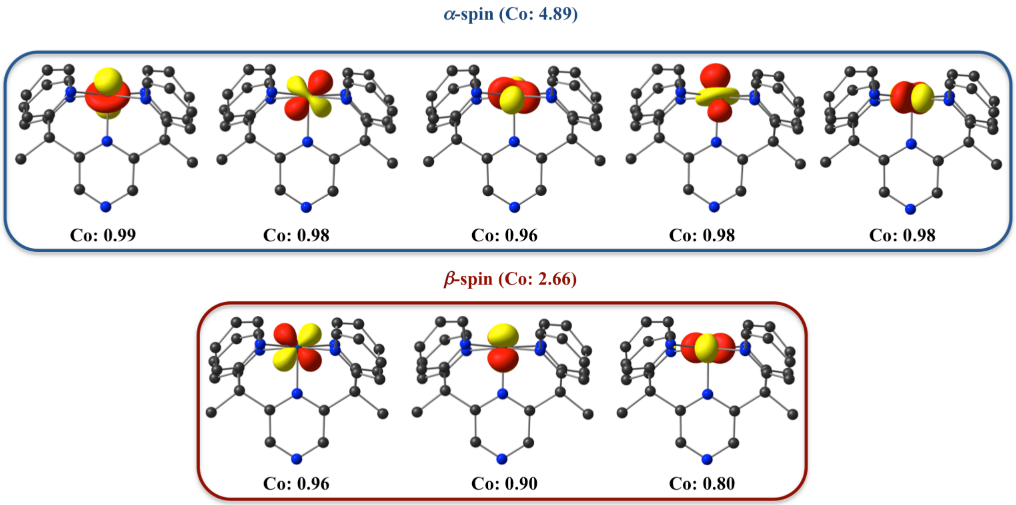

Figure S40. Isosurface (0.07 au) plots of the Edmiston-Ruedenberg localized orbitals for **1'-Co+2e**$^{-}$ (S = 3/2) after dissociation of the acetonitrile molecule using the B3LYP functional. The Löwdin population analyses are given for cobalt.

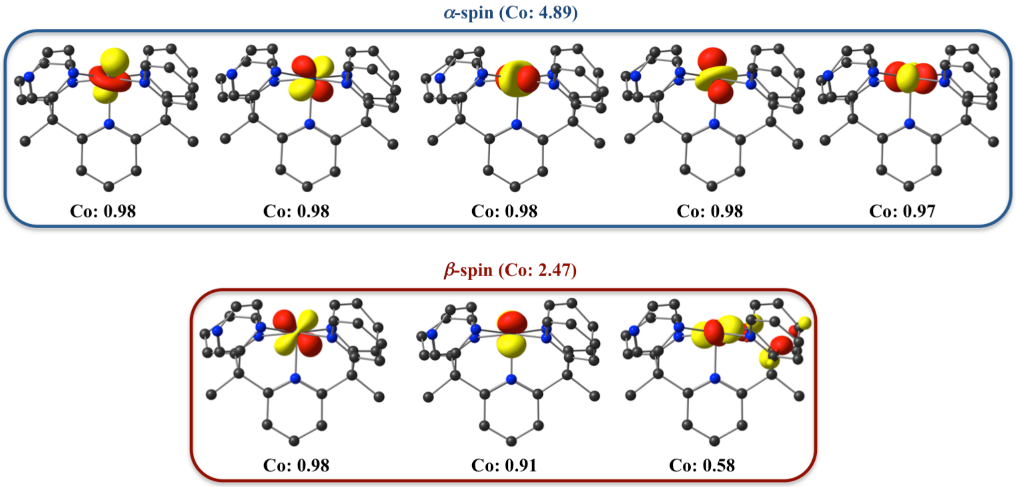

Figure S41. Isosurface (0.07 au) plots of the Edmiston-Ruedenberg localized orbitals for **2'-Co+2e**$^{-}$ (S = 1/2) after dissociation of the acetonitrile molecule using the B3LYP functional. The Löwdin population analyses are given for cobalt.

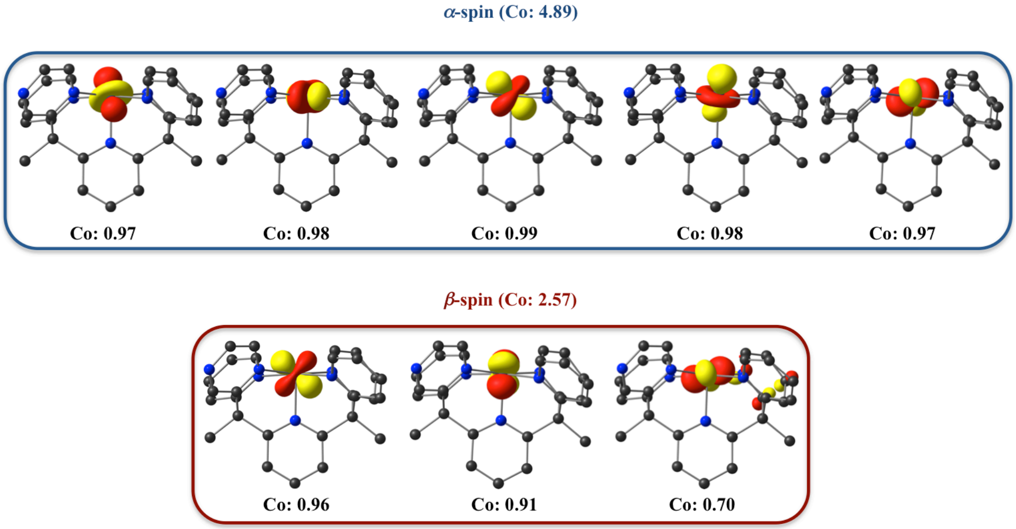

Figure S42. Isosurface (0.07 au) plots of the Edmiston-Ruedenberg localized orbitals for **2'-Co+2e**$^{-}$ (S = 3/2) after dissociation of the acetonitrile molecule using the B3LYP functional. The Löwdin population analyses are given for cobalt.

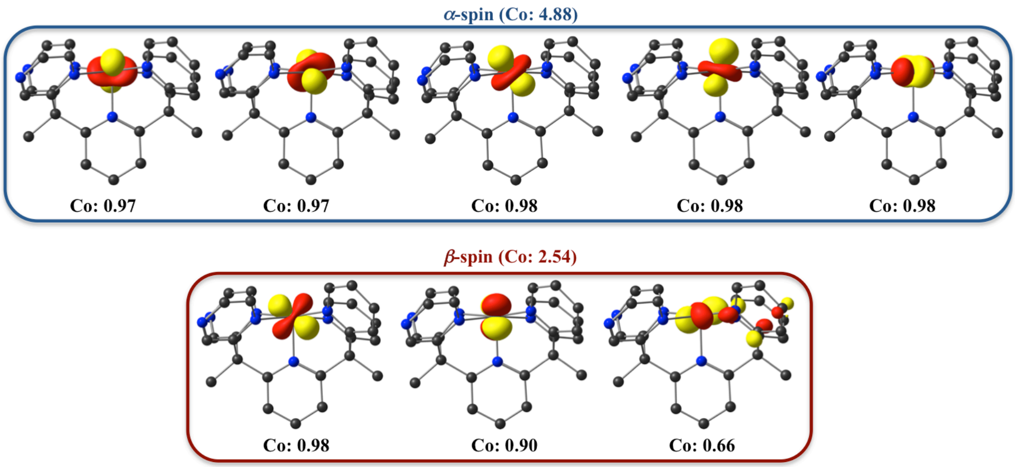

Figure S43. Isosurface (0.07 au) plots of the Edmiston-Ruedenberg localized orbitals for **3'-Co+2e**$^-$ (S = 1/2) after dissociation of the acetonitrile molecule using the B3LYP functional. The Löwdin population analyses are given for cobalt.

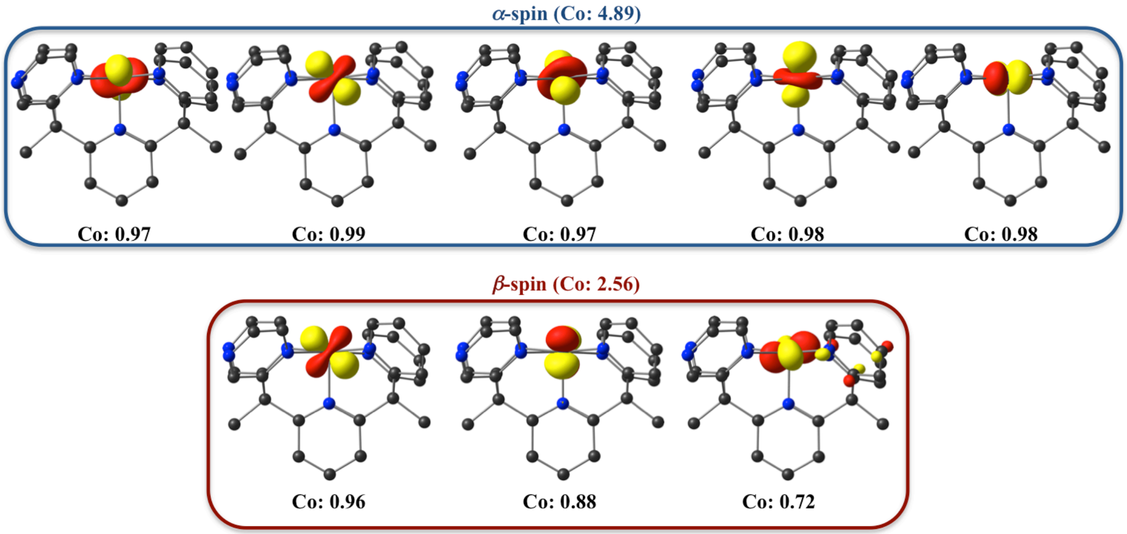

Figure S44. Isosurface (0.07 au) plots of the Edmiston-Ruedenberg localized orbitals for **3'-Co+2e**$^-$ (S = 3/2) after dissociation of the acetonitrile molecule using the B3LYP functional. The Löwdin population analyses are given for cobalt.

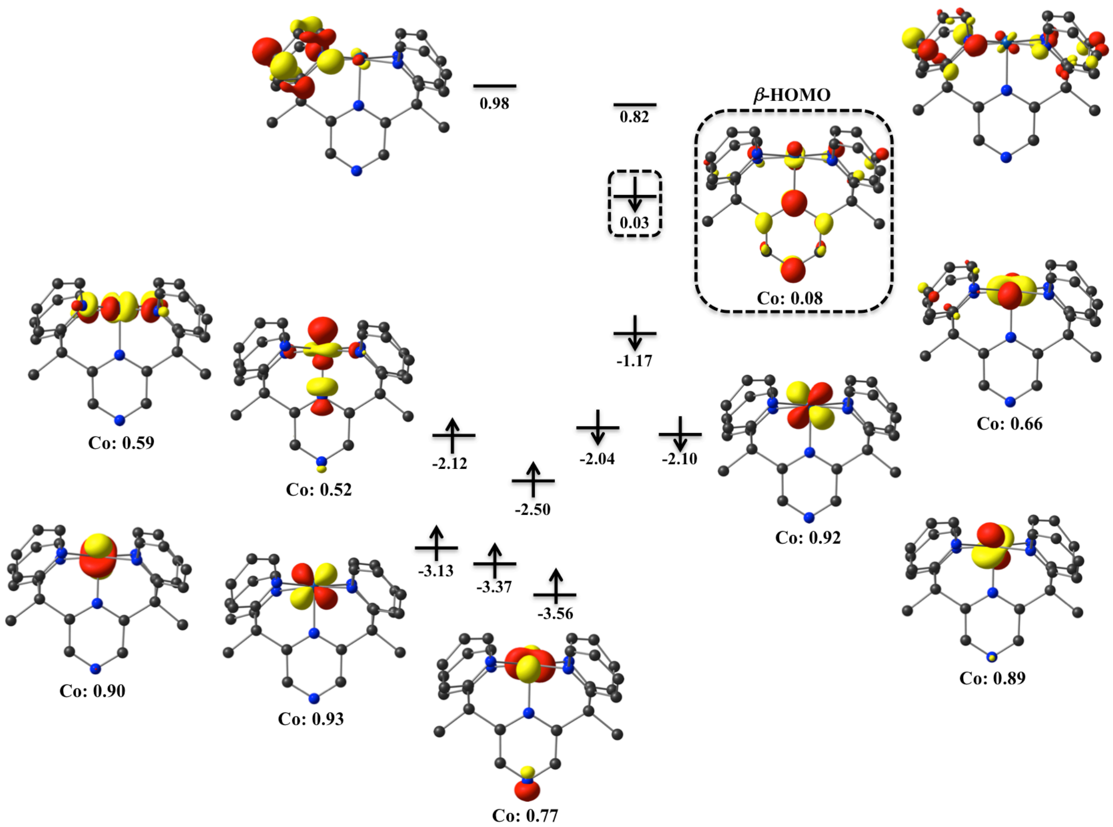

Figure S45. Isosurface (0.07 au) plots of the canonical molecular orbitals for **1'-Co+2e$^-$** (S = 1/2) after dissociation of the acetonitrile molecule using the B3LYP functional. The Löwdin population analyses are given for cobalt. All energies (in eV) are relative to the HOMO (highest occupied molecular orbital) of the six-coordinate species **3'-Co+2e$^-$** (S = 1/2).

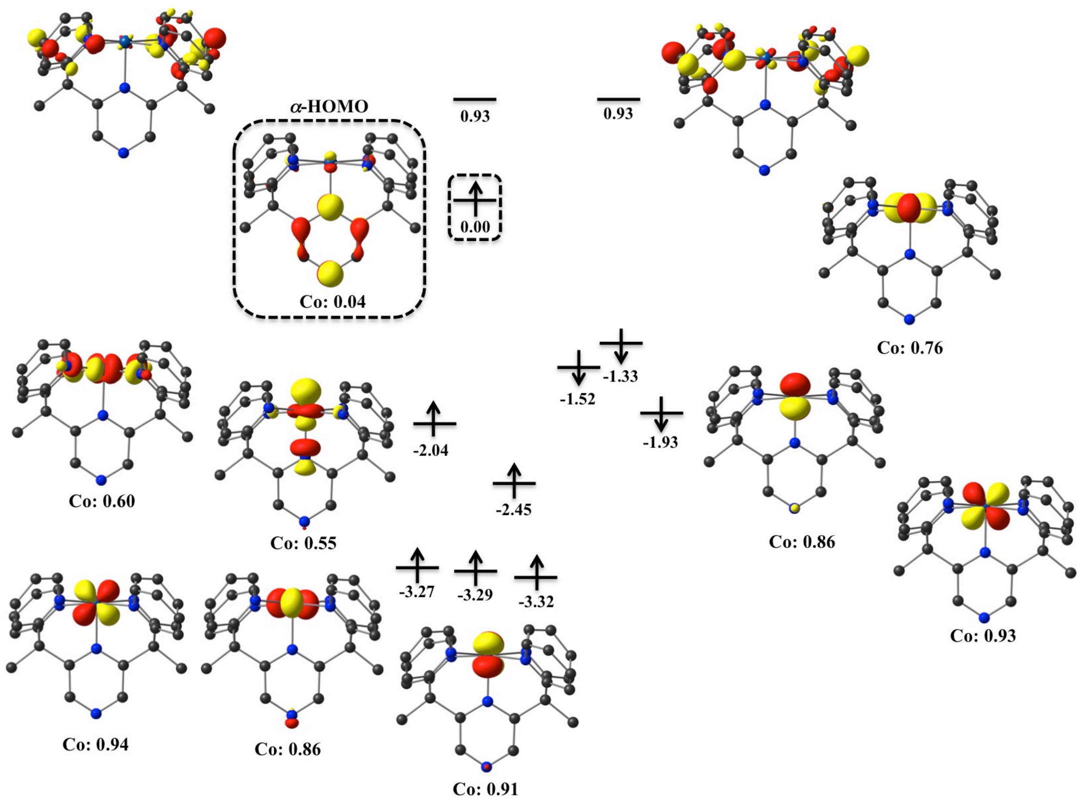

Figure S46. Isosurface (0.07 au) plots of the canonical molecular orbitals for **1'-Co+2e$^-$** (S = 3/2) after dissociation of the acetonitrile molecule using the B3LYP functional. The Löwdin population analyses are given for cobalt. All energies (in eV) are relative to the HOMO (highest occupied molecular orbital) of the six-coordinate species **3'-Co+2e$^-$** (S = 1/2).

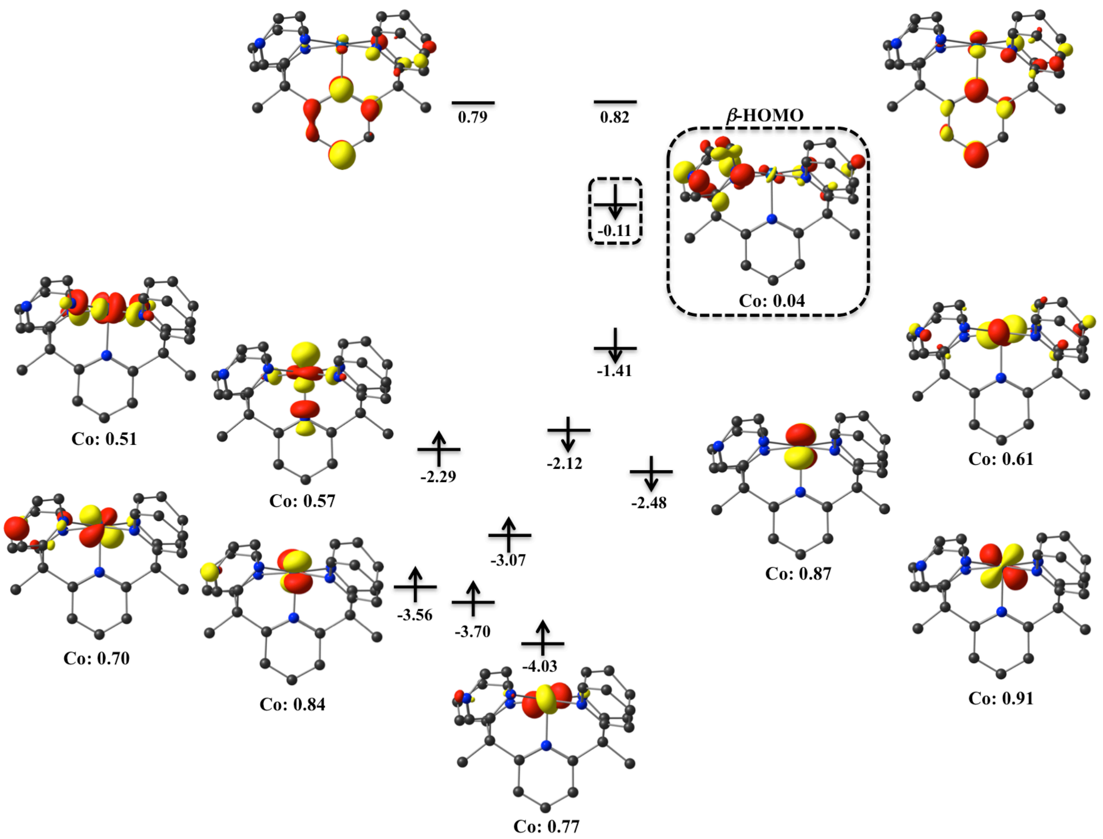

Figure S47. Isosurface (0.07 au) plots of the canonical molecular orbitals for **2'-Co+2e⁻** (S = 1/2) after dissociation of the acetonitrile molecule using the B3LYP functional. The Löwdin population analyses are given for cobalt. All energies (in eV) are relative to the HOMO (highest occupied molecular orbital) of the six-coordinate species **3'-Co+2e⁻** (S = 1/2).

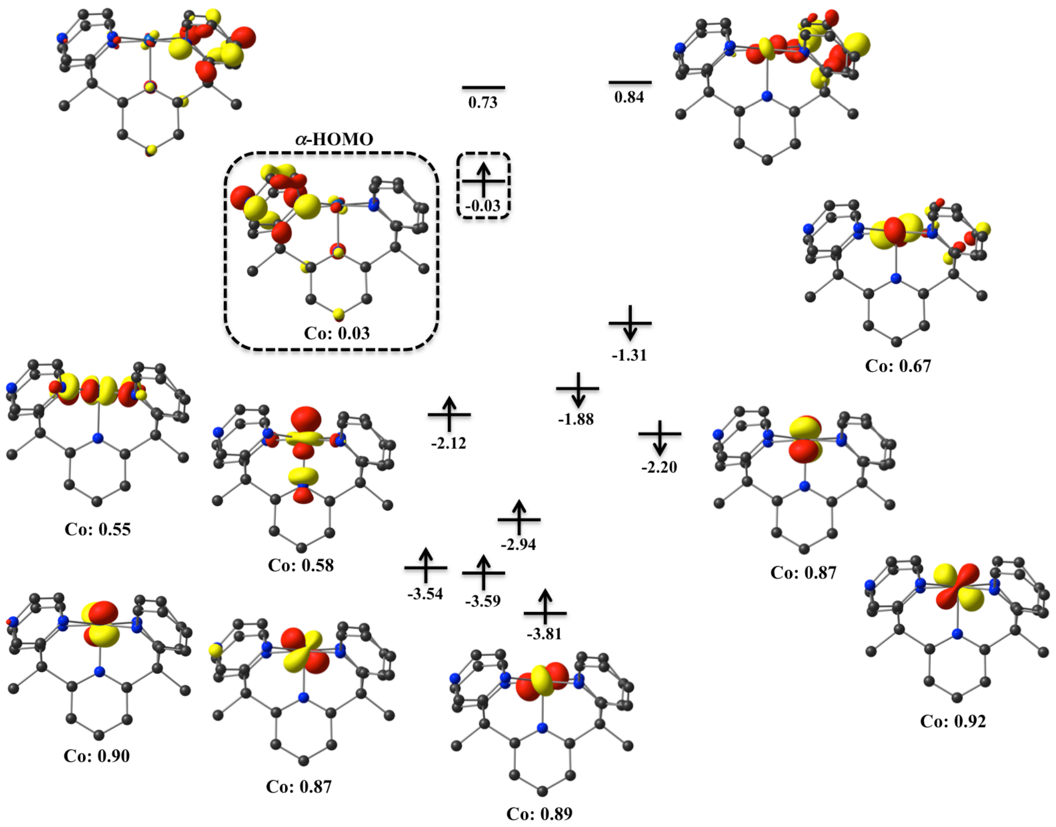

Figure S48. Isosurface (0.07 au) plots of the canonical molecular orbitals for **2'-Co+2e$^-$** (S = 3/2) after dissociation of the acetonitrile molecule using the B3LYP functional. The Löwdin population analyses are given for cobalt. All energies (in eV) are relative to the HOMO (highest occupied molecular orbital) of the six-coordinate species **3'-Co+2e$^-$** (S = 1/2).

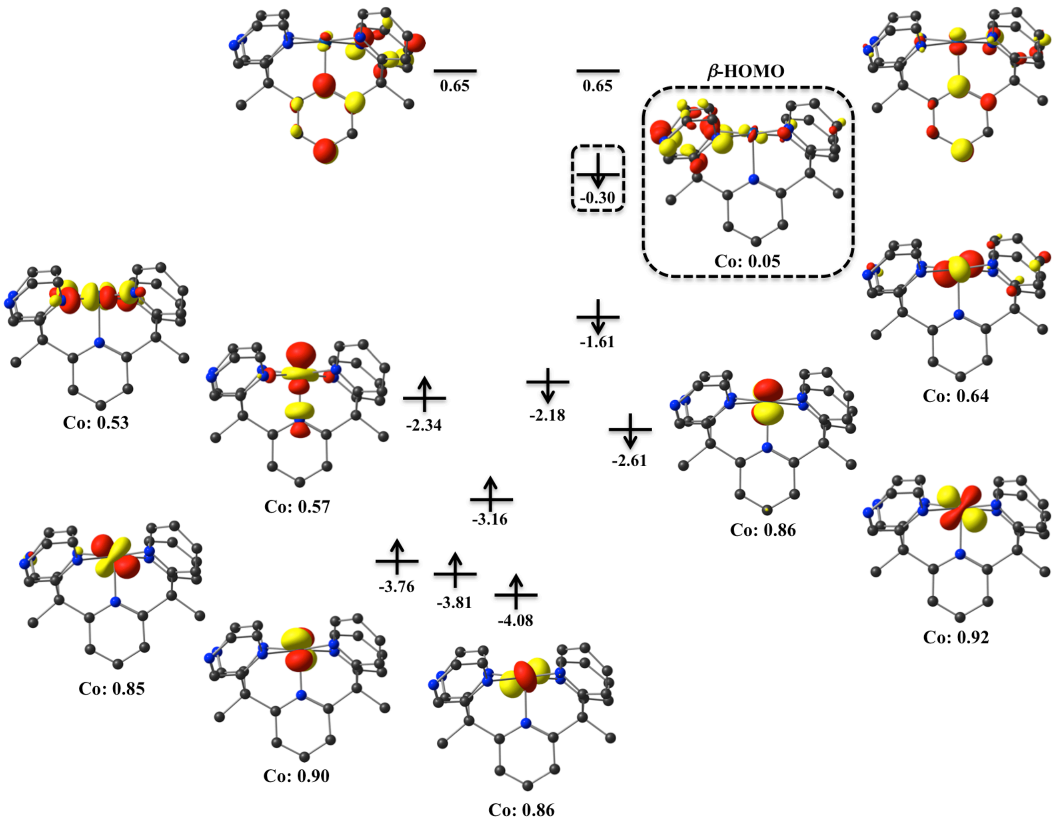

Figure S49. Isosurface (0.07 au) plots of the canonical molecular orbitals for **3'-Co+2e**$^-$ (S = 1/2) after dissociation of the acetonitrile molecule using the B3LYP functional. The Löwdin population analyses are given for cobalt. All energies (in eV) are relative to the HOMO (highest occupied molecular orbital) of the six-coordinate species **3'-Co+2e**$^-$ (S = 1/2).

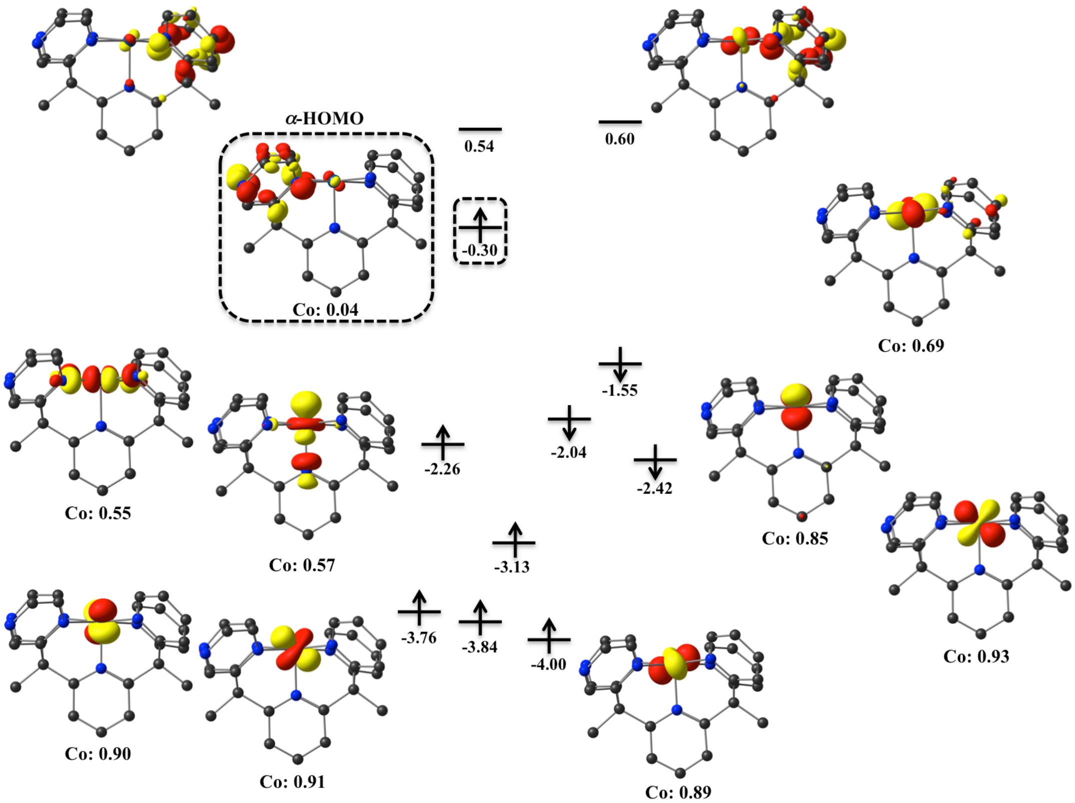

Figure S50. Isosurface (0.07 au) plots of the canonical molecular orbitals for **3'-Co+2e$^-$** (S = 3/2) after dissociation of the acetonitrile molecule using the B3LYP functional. The Löwdin population analyses are given for cobalt. All energies (in eV) are relative to the HOMO (highest occupied molecular orbital) of the six-coordinate species **3'-Co+2e$^-$** (S = 1/2).

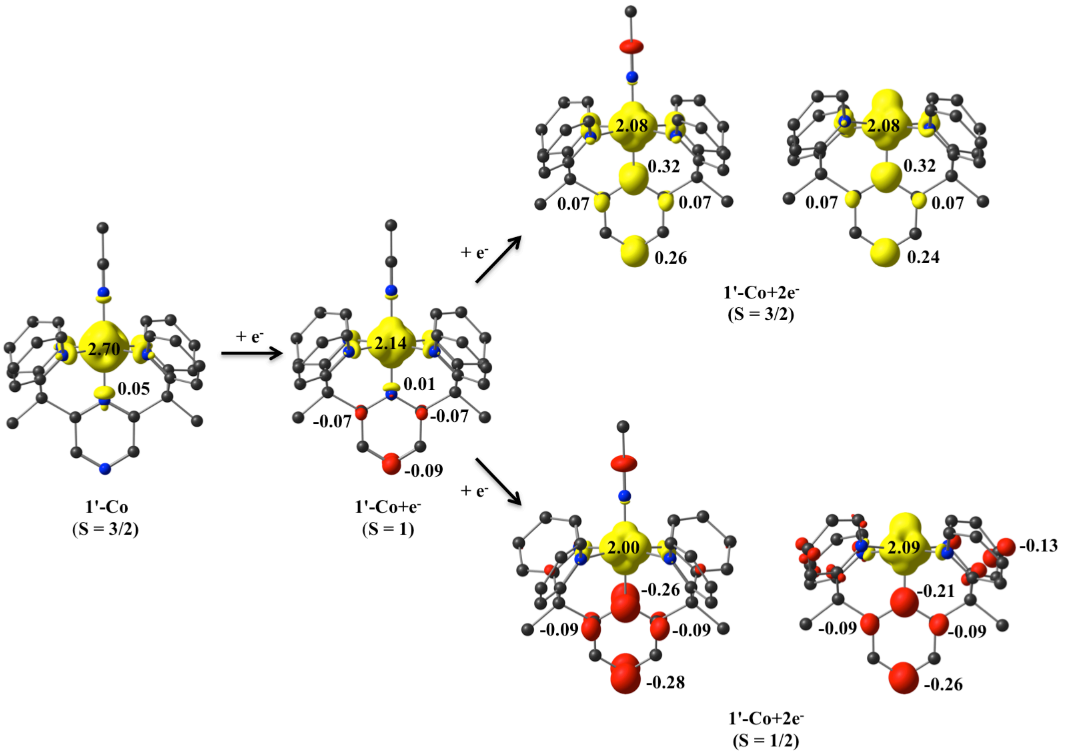

Figure S51. Isosurface (0.007 au) plots of the Mulliken spin population for **1'-Co** and its one and two-electron reduced species.

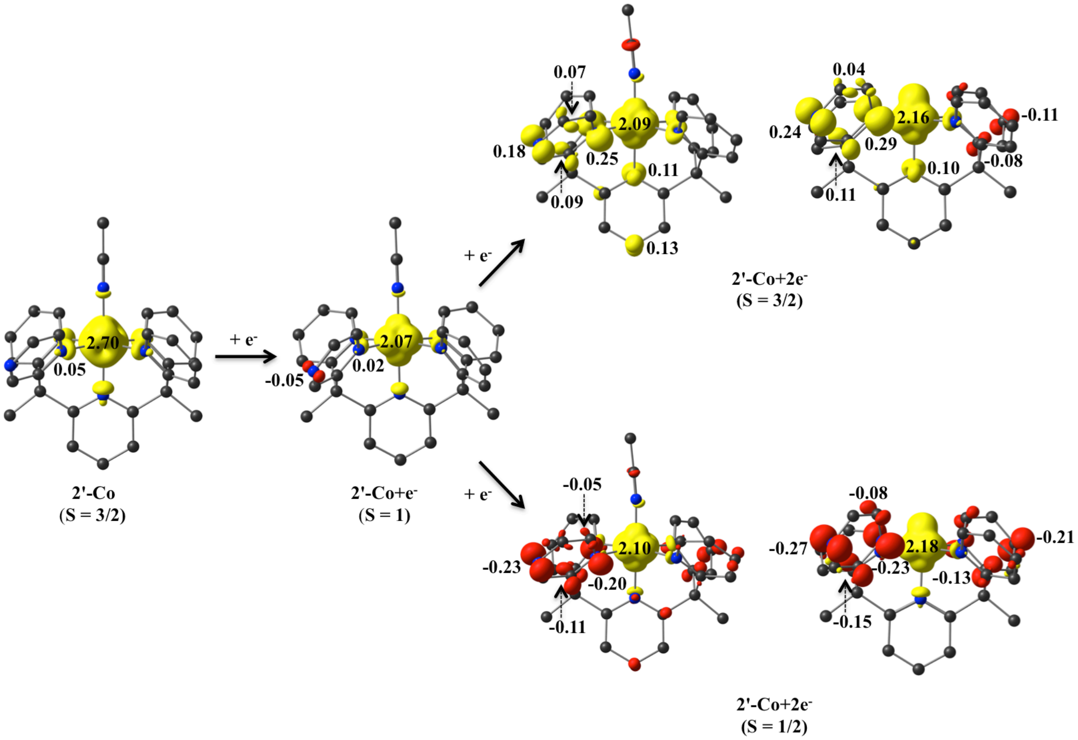

Figure S52. Isosurface (0.007 au) plots of the Mulliken spin population for **2'-Co** and its one and two-electron reduced species.

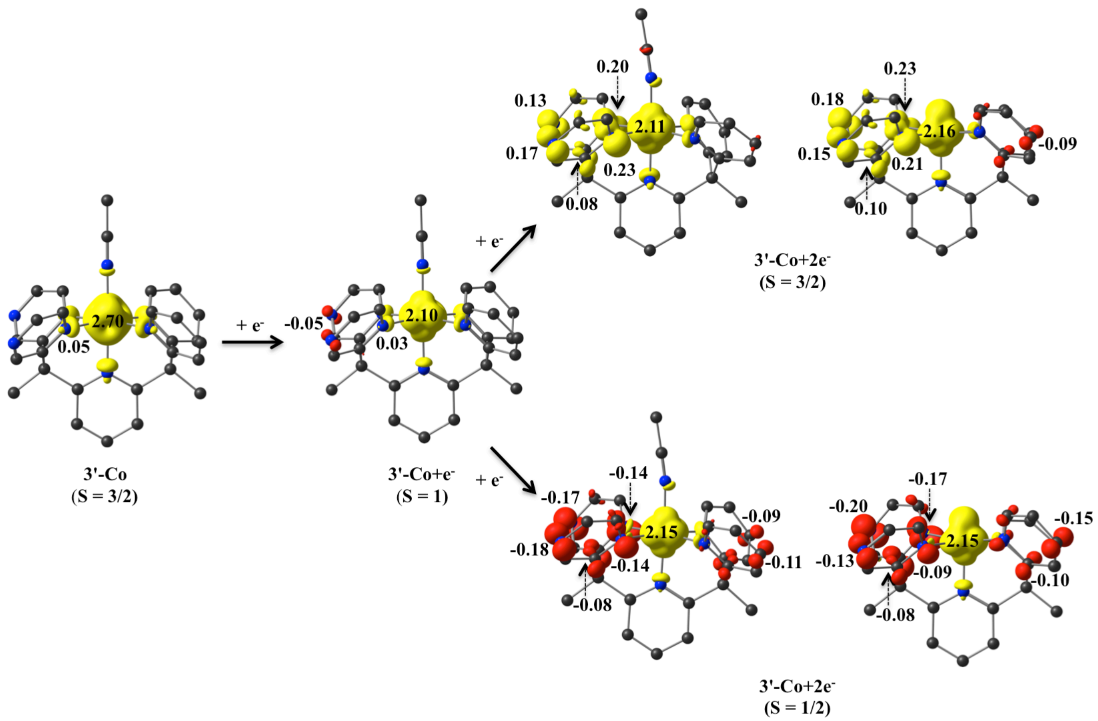

Figure S53. Isosurface (0.007 au) plots of the Mulliken spin population for **3'-Co** and its one and two-electron reduced species.

400 MHz ^{1}H NMR ($CDCl_3$)

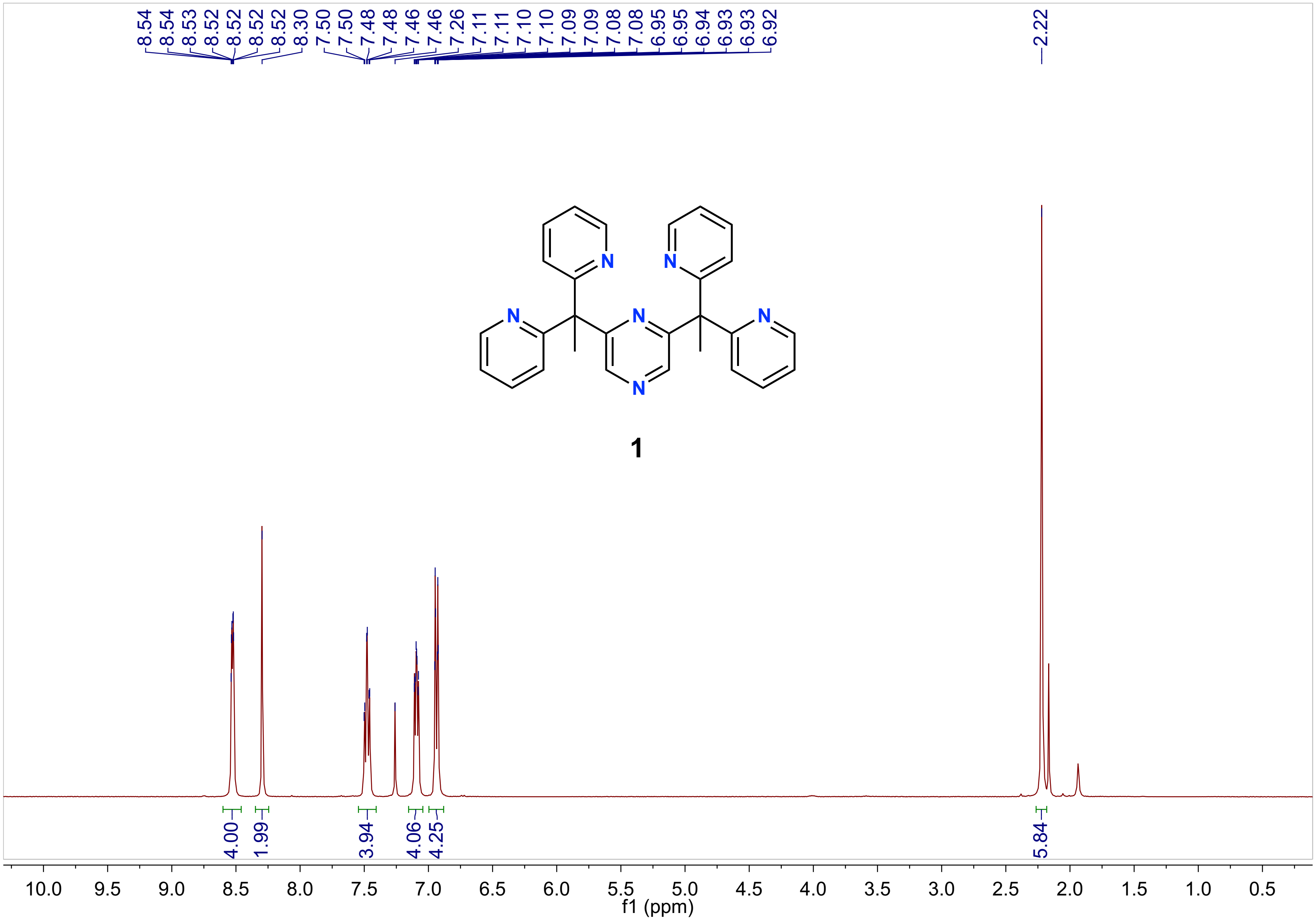

100 MHz ^{13}C NMR ($CDCl_3$)

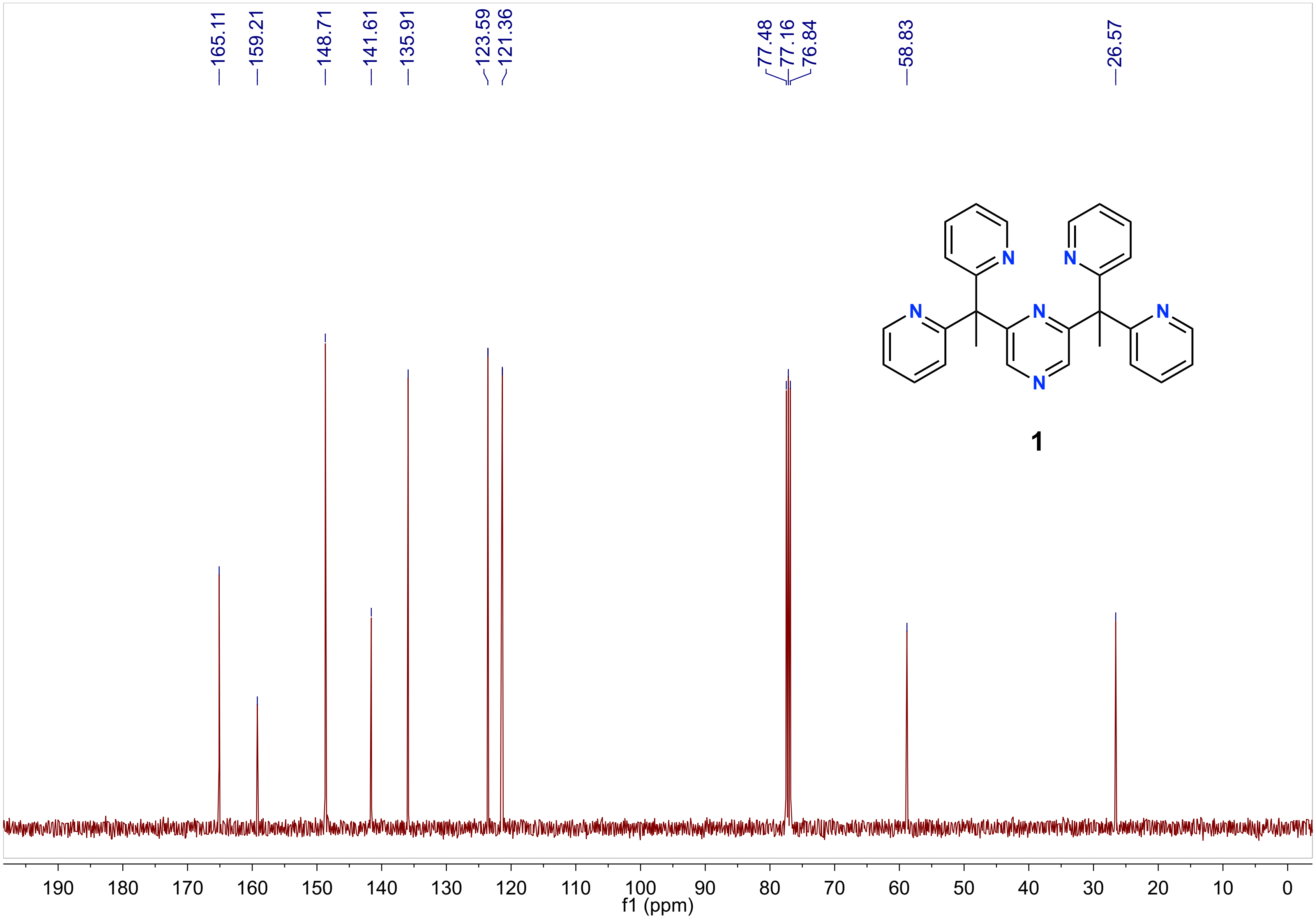

400 MHz ^{1}H NMR ($CDCl_3$)

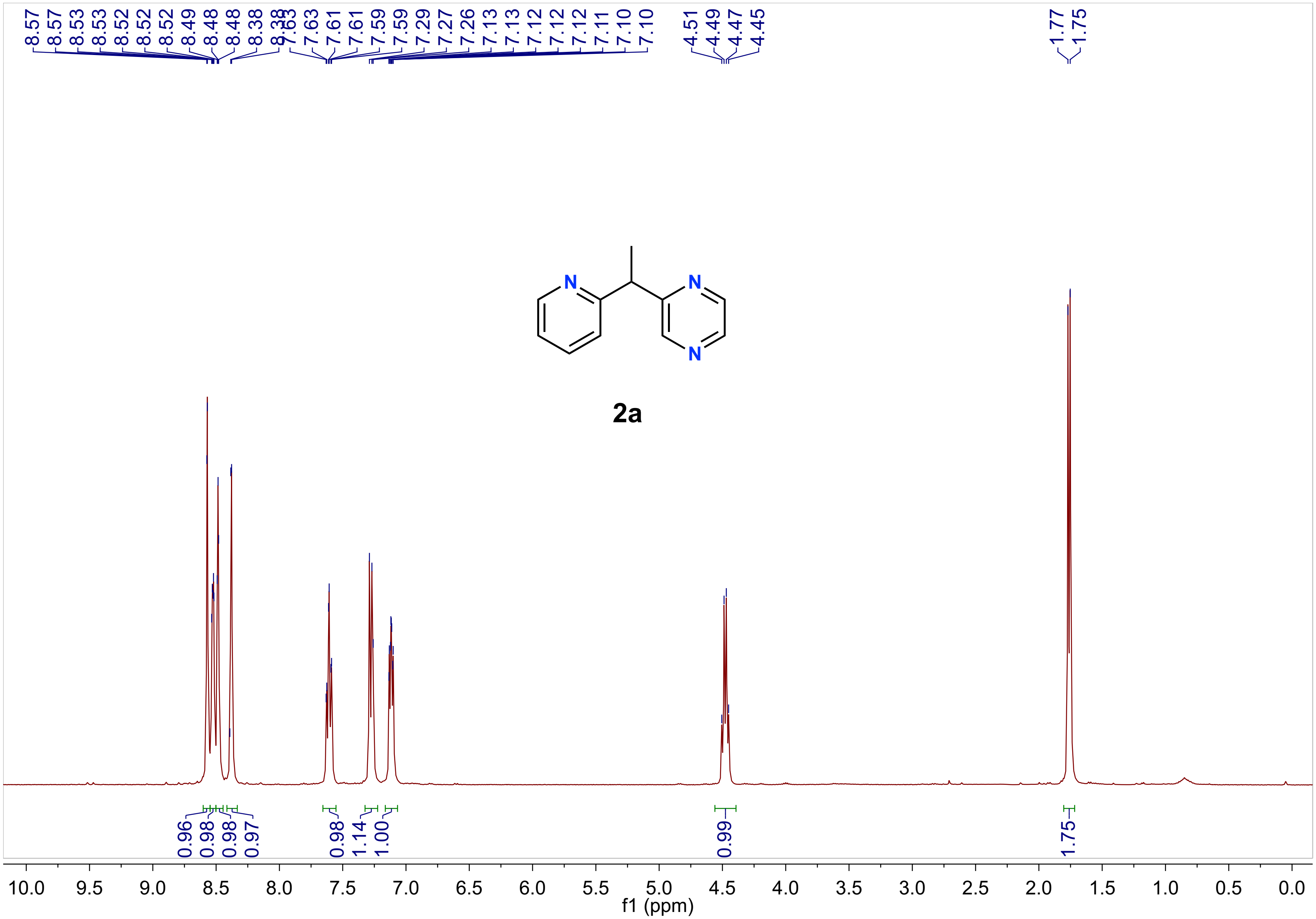

100 MHz ^{13}C NMR ($CDCl_3$)

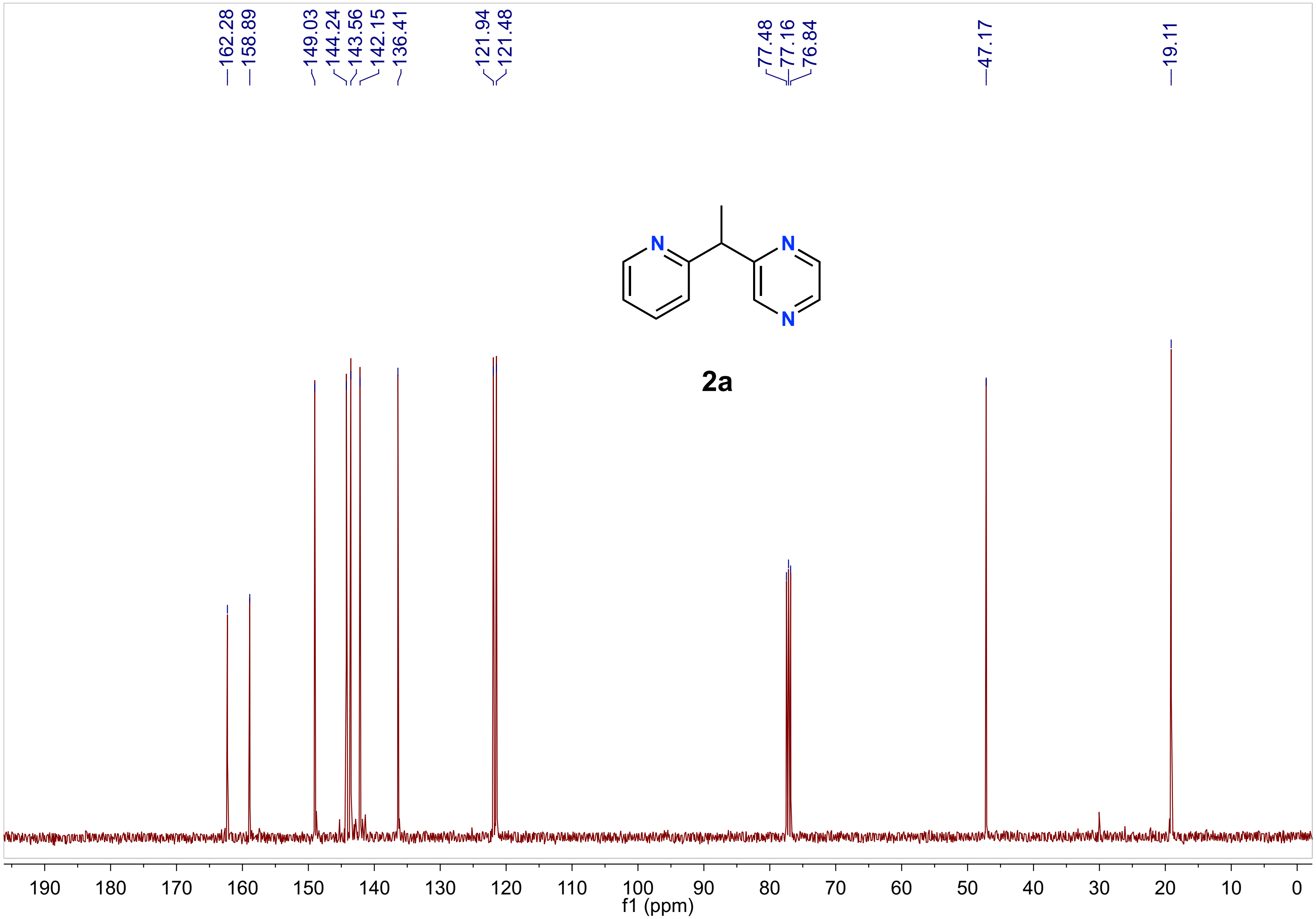

400 MHz ^{1}H NMR ($CDCl_3$)

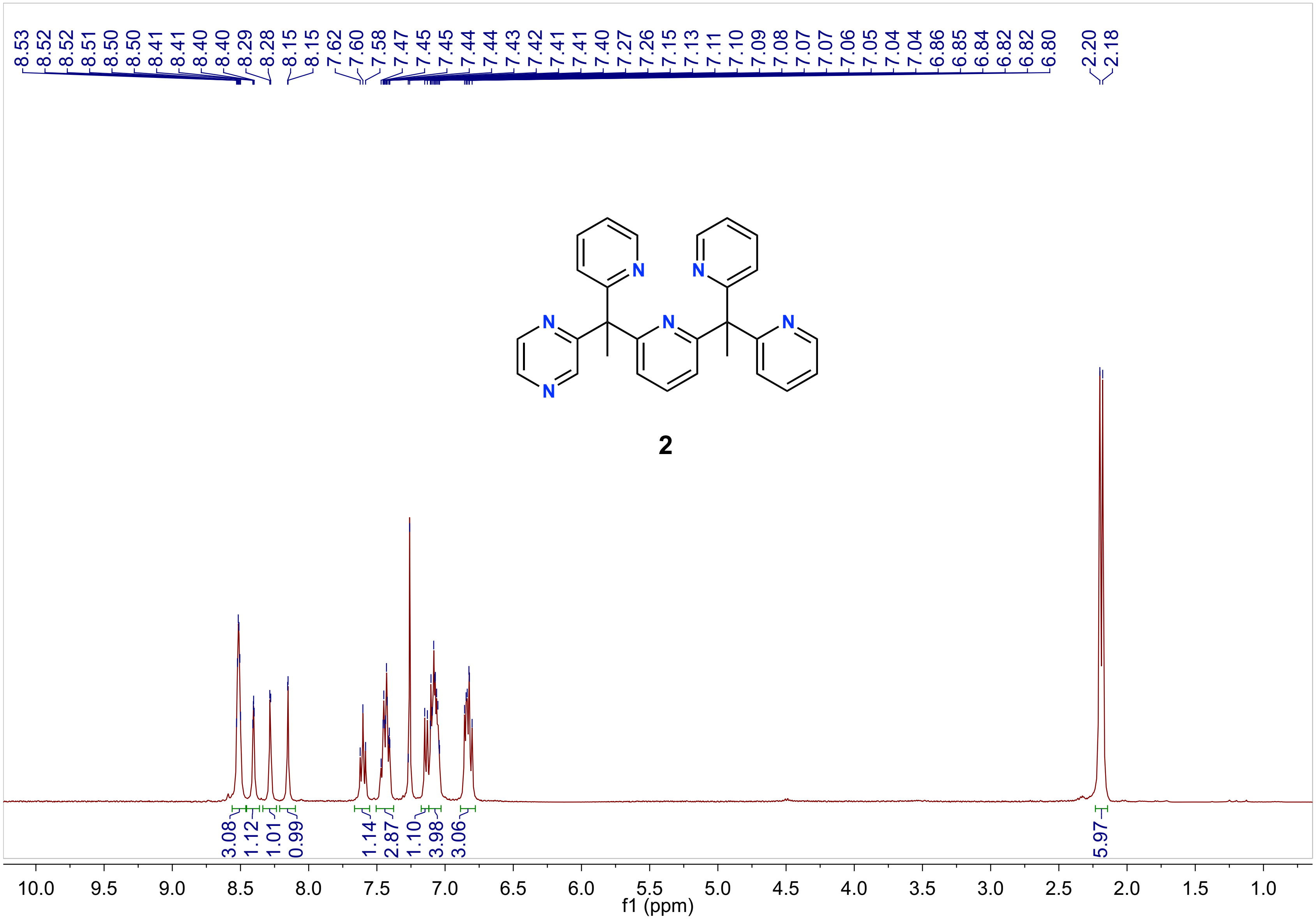

100 MHz ^{13}C NMR ($CDCl_3$)

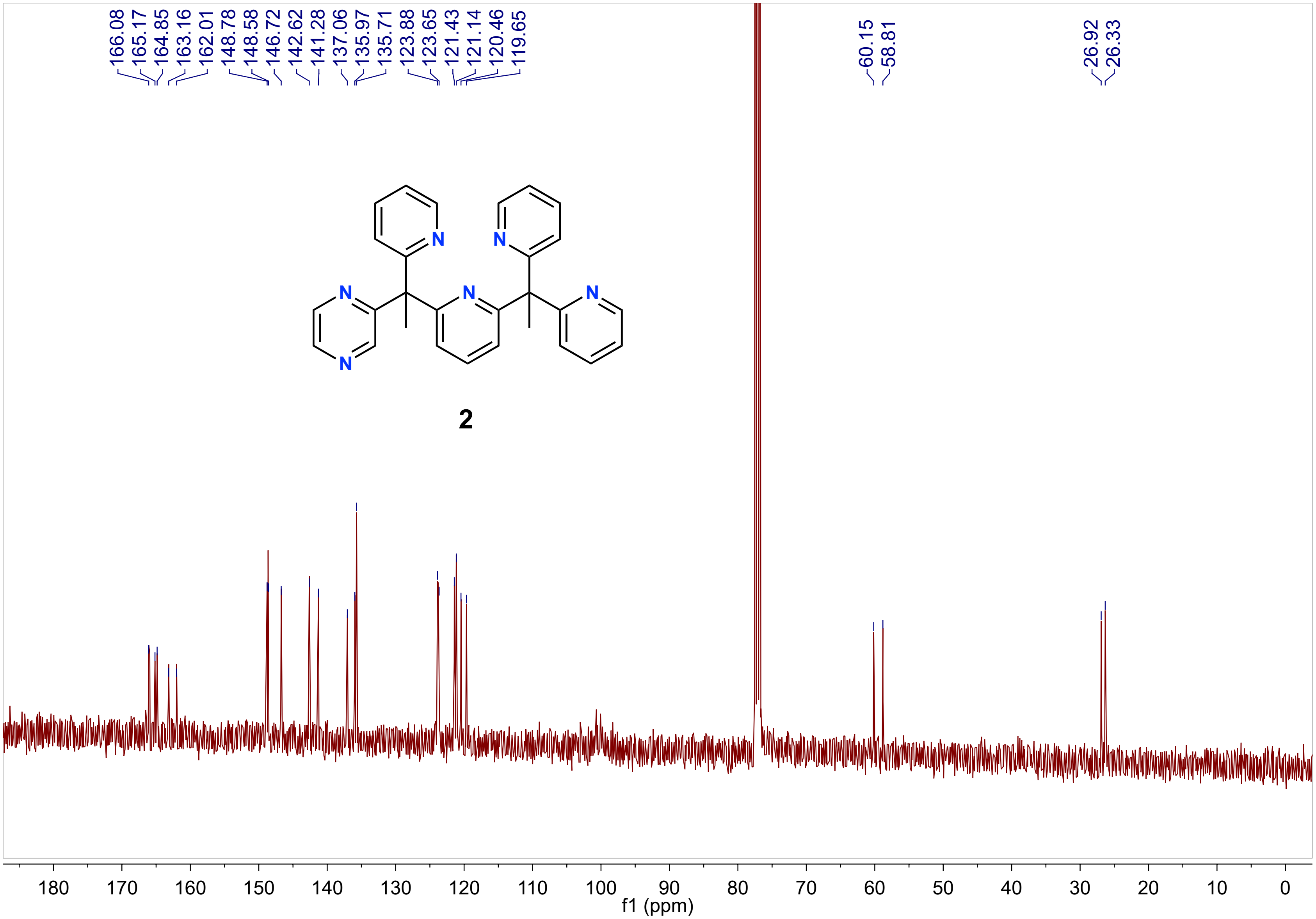

400 MHz ^{1}H NMR ($CDCl_3$)

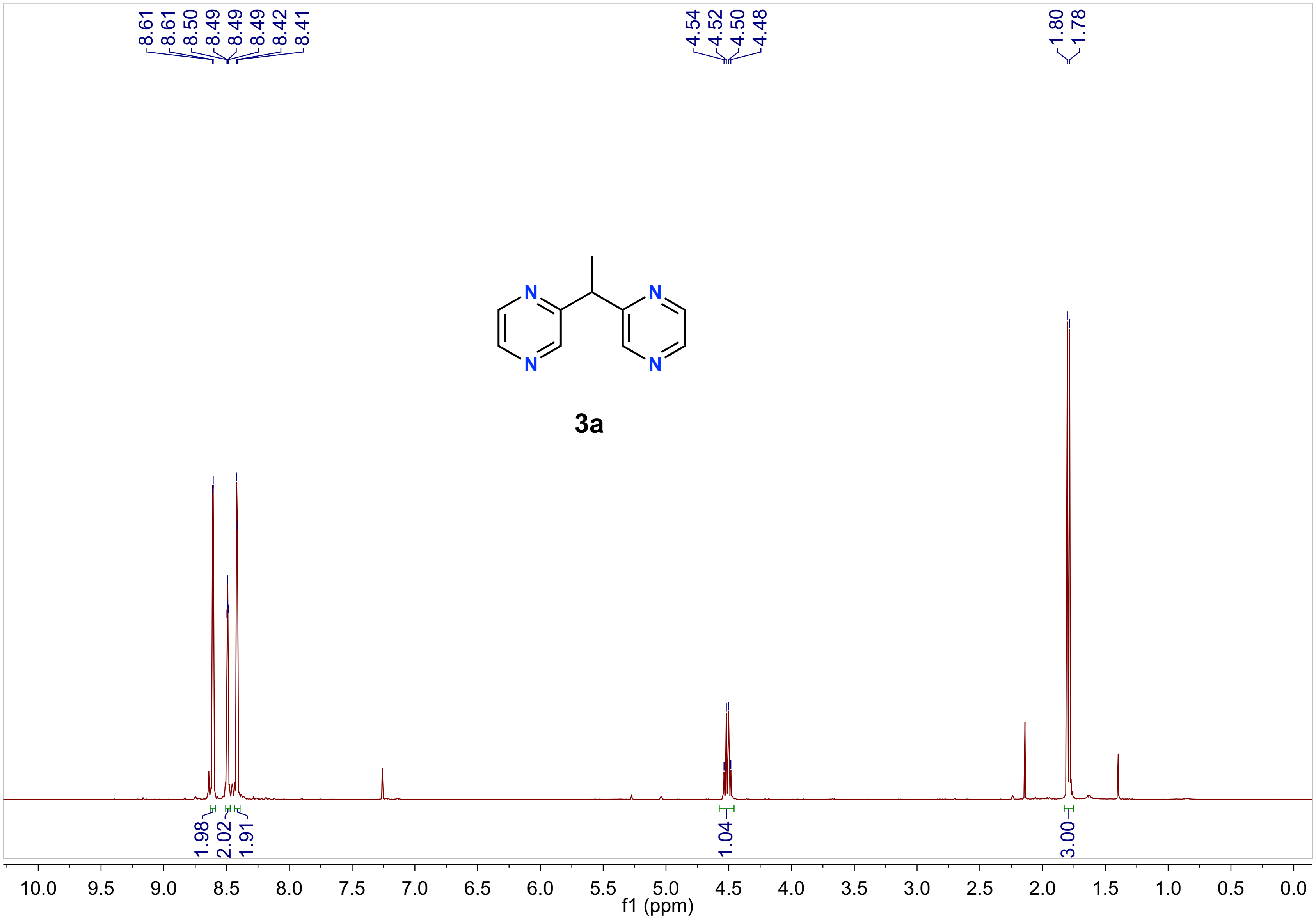

100 MHz ^{13}C NMR ($CDCl_3$)

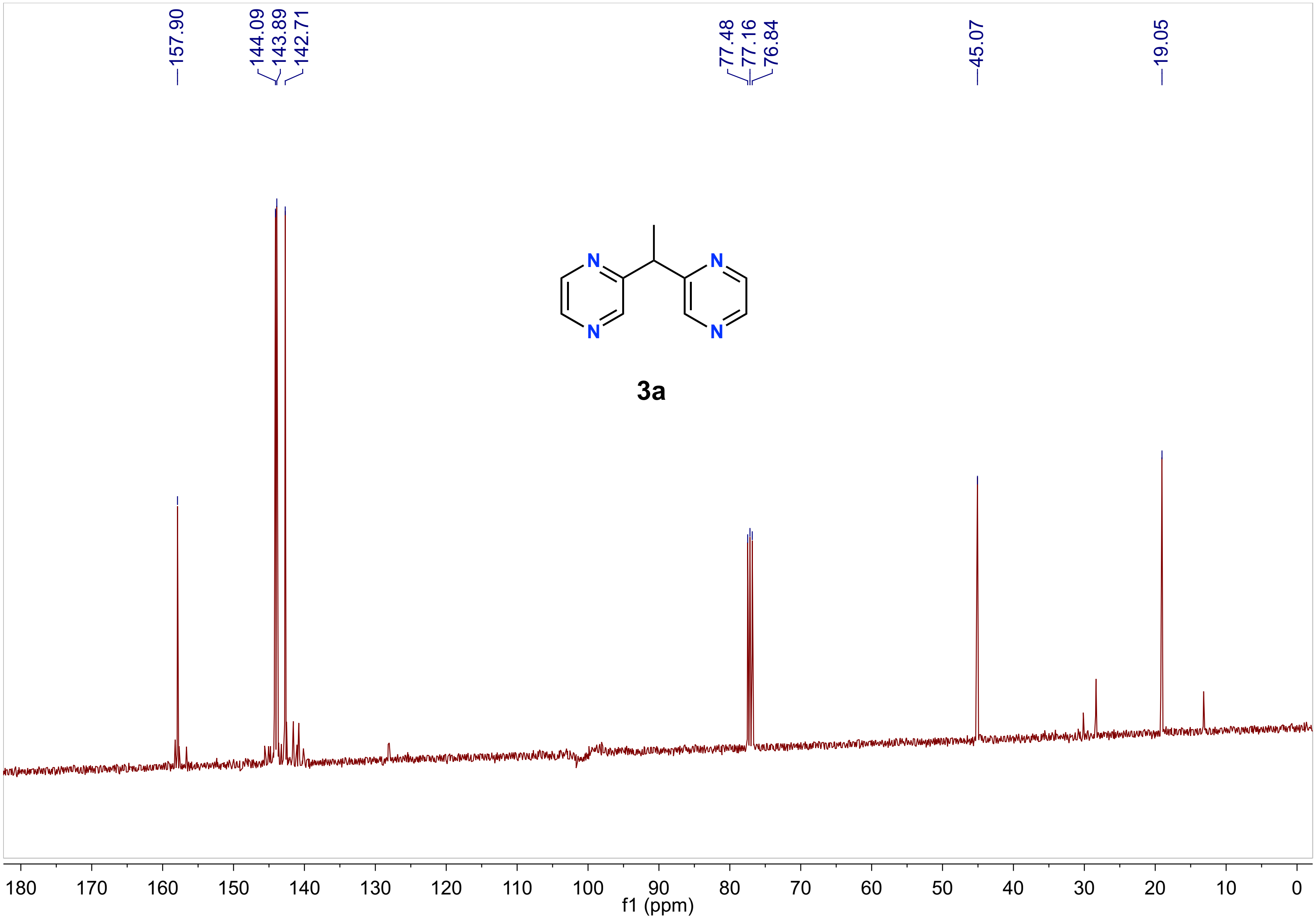

COMPUTED STRUCTURES AND ENERGIES

Acetonitrile (S = 0)

```
Energy              = -132.654863
Enthalpy 0K         = -132.609807
Free Energy 298K    = -132.633844

C  7.53820  2.87961  9.05531
N  7.35471  3.97134  9.39570
C  7.76952  1.50358  8.62616
H  7.74129  0.82322  9.49077
H  6.99555  1.19139  7.90870
H  8.75360  1.41432  8.14150
```

1-Co (S = 3/2)

```
Energy              = -2867.756390
Enthalpy 0K         = -2867.259050
Free Energy 298K    = -2867.326118

H  6.43501  0.74956  5.34748
H  6.41484  2.30667  5.42430
C  7.42130 -1.86318  0.79716
H  8.01657 -2.57896  0.23673
C  8.02685 -0.86415  1.57391
C 10.97646  2.95428  0.22184
H 11.35661  3.88758 -0.19231
C  7.97036  4.29821  1.80543
C 10.08383  0.58861  1.18452
C  9.59643 -0.19100  5.41614
H  9.09973  0.52203  6.07549
C  5.92836 -0.08310  2.20433
H  5.36287  0.63859  2.79507
C  5.88933  3.42498  2.37011
H  5.33993  2.64330  2.89629
C  5.26024 -1.05655  1.46919
H  4.16973 -1.09958  1.47212
C 10.23890 -1.91096  0.92499
H  9.99584 -1.84534 -0.14245
H  9.90219 -2.89105  1.28347
H 11.33127 -1.87992  1.01810
C  9.87447  4.24234  3.45837
C 10.34348  4.28853  6.19220
H 10.48563  4.27199  7.27413
C  9.92727 -0.91798  3.23198
C  9.52009  4.22352  1.94320
C 10.05729  2.92665  1.28645
C 10.99636 -2.08769  5.06745
H 11.64134 -2.89092  5.43132
C  9.56562  3.31776  5.56975
H  9.08587  2.53930  6.16444
C  6.03001 -1.95791  0.73758
H  5.55951 -2.73616  0.13213
C 11.00238  0.67456  0.12258
H 11.40307 -0.21039 -0.37077
C  9.57444 -0.77217  1.72344
C 10.39258 -1.19754  5.95260
H 10.53249 -1.27462  7.03220
C 10.15897  5.44169  1.24798
H 11.25187  5.42488  1.33777
H  9.80317  6.37915  1.69174
H  9.91483  5.46553  0.17901
C  5.20006  4.44780  1.72732
H  4.10899  4.47038  1.73785
C  5.94977  5.42527  1.07716
H  5.46231  6.24603  0.54581
C  7.34283  5.35143  1.12344
H  7.92205  6.12605  0.62810
C 10.92621  5.26585  5.38879
H 11.55628  6.04644  5.82152
C 10.68296  5.24462  4.01463
H 11.12637  6.01934  3.39475
C 10.75501 -1.94939  3.69977
H 11.21514 -2.65653  3.01493
N  9.38068 -0.03971  4.09815
N  7.26732  0.02575  2.24714
N  9.63962  1.73088  1.73795
N 11.43253  1.84175 -0.33963
N  9.34827  3.27965  4.24391
N  7.23020  3.33769  2.39857
Co 8.24504  1.64745  3.31842
O  6.81448  1.55402  4.96292
```

1'-Co (S = 1/2)

```
Energy              = -2924.051520
Enthalpy 0K         = -2923.530613
Free Energy 298K    = -2923.604277
C  7.41806 -1.69830  0.75410
H  7.99318 -2.39994  0.15610
C  8.06097 -0.74038  1.54964
C 11.05156  2.96171  0.15895
H 11.43332  3.89967 -0.24613
C  8.00751  4.17967  1.77472
C 10.15014  0.60482  1.11061
C  9.51006 -0.06776  5.36265
H  8.95398  0.61238  6.00195
C  5.99592  0.03461  2.28045
H  5.45746  0.71139  2.93820
C  5.96082  3.30144  2.43731
H  5.43842  2.55719  3.03211
C  5.29589 -0.90358  1.53030
H  4.20664 -0.94318  1.58322
C 10.23294 -1.89966  0.93088
H  9.99985 -1.85456 -0.14000
H  9.86562 -2.85889  1.31638
H 11.32594 -1.89502  1.02388
C  9.88584  4.12693  3.41955
C 10.25124  4.14835  6.16496
H 10.33642  4.12842  7.25270
C  9.93760 -0.79712  3.19839
C  9.55353  4.17126  1.90511
C 10.12439  2.92166  1.21473
C 10.97707 -1.92649  5.07793
H 11.63065 -2.70960  5.46895
C  9.47165  3.20152  5.51006
H  8.92712  2.45818  6.08580
C  6.02573 -1.77477  0.72705
H  5.52718 -2.52072  0.10387
C 11.07670  0.68022  0.05619
H 11.47847 -0.20878 -0.43163
C  9.60632 -0.71347  1.68532
C 10.30898 -1.05343  5.93121
H 10.39658 -1.12752  7.01641
C 10.15389  5.43322  1.25973
H 11.24689  5.44245  1.35191
H  9.76825  6.34649  1.72976
H  9.91976  5.48017  0.18909
C  5.23993  4.29260  1.78093
```

H 4.15049 4.30729 1.84205
C 5.94939 5.24831 1.05972
H 5.43394 6.04002 0.51123
C 7.34305 5.19537 1.07411
H 7.90210 5.96050 0.54235
C 10.90314 5.10561 5.39332
H 11.54158 5.86331 5.85334
C 10.70189 5.09928 4.01323
H 11.17976 5.86684 3.41030
C 10.77321 -1.80207 3.70390
H 11.26519 -2.50359 3.03552
N 9.35116 0.09166 4.03663
N 7.33592 0.15010 2.26897
N 9.70036 1.73602 1.66053
N 11.51208 1.84886 -0.40204
N 9.31725 3.15616 4.17478
N 7.30260 3.21248 2.41006
Co 8.29132 1.64999 3.26248
C 5.95397 1.48719 5.93223
N 6.71416 1.54784 5.05965
C 4.99787 1.41027 7.02833
H 5.52765 1.26403 7.98220
H 4.41321 2.34119 7.08656
H 4.31118 0.56415 6.87218

1'-Co (S = 3/2)

Energy = -2924.055978
Enthalpy 0K = -2923.536544
Free Energy 298K = -2923.611360
C 7.42395 -1.86090 0.79014
H 8.02276 -2.57708 0.23424
C 8.02395 -0.86282 1.57258
C 10.98144 2.95193 0.24416
H 11.36287 3.88571 -0.16732
C 7.96943 4.29702 1.80375
C 10.08259 0.58984 1.20161
C 9.60754 -0.19545 5.41949
H 9.11964 0.52083 6.08151
C 5.92273 -0.08156 2.18747
H 5.35356 0.63803 2.77613
C 5.88489 3.42315 2.34763
H 5.33111 2.64264 2.86963
C 5.25920 -1.05178 1.44374
H 4.16866 -1.09239 1.43623
C 10.23694 -1.90879 0.92711
H 9.99828 -1.83718 -0.14087
H 9.89728 -2.89027 1.27875
H 11.32890 -1.88009 1.02519
C 9.87005 4.24930 3.46545
C 10.34200 4.29945 6.19759
H 10.48655 4.28308 7.27915
C 9.92308 -0.92604 3.23612
C 9.51840 4.22406 1.95114
C 10.05754 2.92381 1.30467
C 10.98835 -2.10541 5.06744
H 11.62670 -2.91480 5.42927
C 9.56927 3.32409 5.57596
H 9.09422 2.54442 6.17246
C 6.03327 -1.95352 0.71791
H 5.56715 -2.73021 0.10710
C 11.00553 0.67522 0.14337
H 11.40579 -0.21028 -0.34898
C 9.57100 -0.77347 1.72954
C 10.39848 -1.20731 5.95357
H 10.54416 -1.28319 7.03244
C 10.15982 5.43935 1.25298
H 11.25222 5.42393 1.34854
H 9.80116 6.37850 1.69069
H 9.92076 5.45829 0.18285
C 5.20103 4.44426 1.69616
H 4.10990 4.46475 1.69391
C 5.95585 5.42315 1.05502
H 5.47344 6.24364 0.51866
C 7.34826 5.35079 1.11663
H 7.93159 6.12660 0.62829
C 10.91512 5.28223 5.39432
H 11.53897 6.06813 5.82638
C 10.66971 5.25935 4.02086
H 11.10474 6.03834 3.40057
C 10.74141 -1.96643 3.70114
H 11.19015 -2.67931 3.01487
N 9.38795 -0.04276 4.10314
N 7.26031 0.02658 2.24122
N 9.63659 1.72987 1.75874
N 11.44076 1.84092 -0.31696
N 9.35208 3.28324 4.25100
N 7.22440 3.33617 2.38961
Co 8.22632 1.64582 3.35380
C 6.00753 1.50079 5.83811
N 6.76385 1.55385 4.96383
C 5.05477 1.43394 6.93509
H 5.58867 1.29326 7.88775
H 4.47332 2.36744 6.98706
H 4.36705 0.58727 6.78538

1-Zn (S = 0)

Energy = -3264.356949
Enthalpy 0K = -3263.860288
Free Energy 298K = -3263.928858

C 10.67388 12.82400 18.68653
H 10.79336 12.92532 19.76592
C 10.75594 13.94656 17.86883
H 10.94429 14.93152 18.29938
C 10.59231 13.76210 16.49826
H 10.65470 14.60575 15.80686
C 10.33567 12.47843 16.01296
H 10.19746 12.34513 14.94360
C 10.25399 11.39322 16.89806
C 9.91269 9.95598 16.39588
C 9.69929 9.98295 14.86897
H 10.59997 10.32775 14.34684
H 8.87750 10.65574 14.59619
H 9.45567 8.98600 14.48240
C 8.59846 9.48595 17.09284
C 7.46331 9.12276 16.35274
H 7.47997 9.10691 15.26643
C 6.27848 8.78138 17.00762
H 5.39608 8.49854 16.42875
C 6.23674 8.82500 18.39930
H 5.32755 8.59190 18.95611
C 7.40333 9.17794 19.06941
H 7.41646 9.23919 20.15873
C 11.08088 8.98649 16.71890
C 11.78381 8.29120 15.71786
H 11.54156 8.39047 14.66013
C 13.12972 7.30772 17.28097
H 13.96014 6.62699 17.46663

```
C 12.45743  7.97497 18.32143
C  9.45388  7.49168 21.39980
H  8.57335  8.13402 21.43899
C  9.46456  6.28193 22.08663
H  8.59372  5.96607 22.66358
C 10.61549  5.50191 22.00600
H 10.67795  4.53899 22.51841
C 11.70174  5.97100 21.26489
H 12.59987  5.36170 21.21590
C 12.83782  7.78565 19.81423
C 14.02342  6.80373 19.90202
H 13.76418  5.82623 19.47780
H 14.32940  6.64485 20.94292
H 14.89447  7.18080 19.35265
C 13.24656  9.14714 20.45615
C 14.53468  9.36097 20.96945
H 15.30715  8.60114 20.88861
C 14.84474 10.56328 21.60788
H 15.84870 10.72732 22.00645
C 13.85480 11.53410 21.74208
H 14.04052 12.47943 22.25478
C 12.60525 11.26182 21.19535
H 11.79404 11.98582 21.28640
C 11.62455  7.20743 20.60661
N 10.44324 11.58473 18.22180
N  8.55360  9.47763 18.44262
N 11.44776  8.79886 17.99707
N 12.78696  7.47205 16.00912
N 10.49135  7.93952 20.67310
N 12.31261 10.11765 20.55426
O  9.31130 10.89622 21.16352
Zn 10.40886  9.81991 19.53383
H  8.60799 11.55332 21.05315
H  9.45352 10.79855 22.11685
```

2-Co (S = 3/2)

```
Energy              = -2867.755038
Enthalpy 0K         = -2867.257601
Free Energy 298K    = -2867.324549
```

```
C  9.17942  3.14031  6.74661
H  9.97745  2.45284  7.03031
C  9.22501  3.79354  5.51977
H 10.05109  3.61518  4.82920
C  8.18648  4.67029  5.21372
H  8.16337  5.20191  4.25954
C  7.17620  4.87679  6.15427
H  6.38022  5.57781  5.91804
C  7.20438  4.19409  7.37964
C  6.14215  4.45566  8.48738
C  5.18365  5.57070  8.02321
H  4.42972  5.79436  8.78752
H  4.65257  5.28405  7.10793
H  5.73007  6.49821  7.81318
C  6.88069  4.92452  9.76755
C  6.63222  6.17631 10.36857
H  5.87096  6.85448  9.98308
C  8.24731  5.81969 11.93229
H  8.81484  6.17763 12.79759
C  8.50347  4.56863 11.37061
H  9.27739  3.92064 11.78299
C  5.33003  3.15973  8.78066
C  3.94426  3.12365  8.58761
H  3.39513  3.98918  8.22931
C  3.25085  1.94924  8.86043
H  2.16906  1.90295  8.71501
C  3.94385  0.83438  9.31936
H  3.39300 -0.07747  9.52852
C  5.33042  0.91374  9.49821
C  8.60048  1.21962 12.36680
H  9.40364  1.94948 12.25381
C  8.39744  0.58834 13.58906
H  9.02887  0.82753 14.44651
C  6.61990 -0.63216 12.52844
H  5.83315 -1.37883 12.59378
C  6.89337  0.03214 11.32277
C  6.14220 -0.31498 10.00432
C  5.17979 -1.48974 10.26682
H  4.64949 -1.78526  9.35378
H  4.42544 -1.22723 11.01798
H  5.72345 -2.36808 10.63489
C  7.19411 -0.74186  8.93791
C  7.17532 -2.01754  8.35360
H  6.39940 -2.73755  8.59852
C  8.16754 -2.38825  7.44486
H  8.15117 -3.38218  6.99157
C  9.17663 -1.47833  7.13848
H  9.98440 -1.72429  6.44706
C  9.12456 -0.22767  7.74433
H  9.89757  0.51217  7.53369
N  8.19371  3.31529  7.64366
N  7.81712  4.12375 10.30868
N  5.98265  2.06495  9.22749
N  7.86095  0.97017 11.27231
N  8.16060  0.13976  8.60582
O 10.25083  2.22332  9.73281
Co 8.07714  2.12879  9.45725
H 10.82835  2.97993  9.55113
H 10.82309  1.48662  9.99538
C  7.36813 -0.34704 13.67142
H  7.15353 -0.86411 14.60960
N  7.30173  6.60919 11.43332
```

2'-Co (S = 1/2)

```
Energy              = -2924.050471
Enthalpy 0K         = -2923.529607
Free Energy 298K    = -2923.603495
```

```
C  9.15714  3.01032  6.87134
H  9.96718  2.36356  7.19643
C  9.22222  3.62809  5.62772
H 10.07238  3.44265  4.96919
C  8.18220  4.47896  5.26501
H  8.17334  4.97852  4.29356
C  7.15889  4.70985  6.18383
H  6.36505  5.40372  5.92073
C  7.16554  4.06801  7.42972
C  6.10389  4.39826  8.51080
C  5.21440  5.56582  8.04535
H  4.46312  5.81862  8.80321
H  4.67333  5.30717  7.12739
H  5.80723  6.46703  7.84398
C  6.86373  4.79540  9.79580
C  6.63969  6.02721 10.44294
H  5.86240  6.71022 10.10049
C  8.31795  5.63618 11.91851
H  8.93317  5.97352 12.75896
C  8.55066  4.40005 11.31722
```

```
H  9.35934  3.76317 11.66428
C  5.23612  3.15091  8.78526
C  3.84904  3.13997  8.59897
H  3.31021  4.01736  8.25059
C  3.14706  1.96801  8.86950
H  2.06388  1.93155  8.73252
C  3.83568  0.84319  9.31622
H  3.28490 -0.06985  9.52613
C  5.22373  0.92295  9.48223
C  8.59580  1.27809 12.25353
H  9.43075  1.95908 12.11016
C  8.40559  0.66407 13.48589
H  9.07476  0.88837 14.31821
C  6.57587 -0.51601 12.48614
H  5.77657 -1.24715 12.57019
C  6.83609  0.12304 11.26583
C  6.07591 -0.26786  9.97101
C  5.16848 -1.48194 10.23983
H  4.63089 -1.78369  9.33291
H  4.41299 -1.25035 11.00024
H  5.74785 -2.34476 10.59164
C  7.14095 -0.62538  8.90031
C  7.12814 -1.86520  8.24641
H  6.32528 -2.57605  8.42085
C  8.15612 -2.21415  7.37102
H  8.14166 -3.18064  6.86209
C  9.20756 -1.32059  7.18947
H 10.06160 -1.55582  6.55223
C  9.14825 -0.10012  7.85210
H  9.96664  0.60789  7.75477
N  8.14403  3.18399  7.73920
N  7.81006  3.96480 10.28623
N  5.88282  2.05914  9.21648
N  7.81339  1.05702 11.18171
N  8.13092  0.26556  8.65249
Co 8.01903  2.11741  9.46965
C  7.34955 -0.23401 13.61161
H  7.14147 -0.73169 14.56154
N  7.34519  6.43446 11.49343
C 11.56109  2.29700  9.88652
N 10.41165  2.23108  9.75345
C 13.00538  2.38015 10.05568
H 13.51253  2.08448  9.12442
H 13.29812  3.41136 10.30631
H 13.33058  1.70797 10.86457
```

2'-Co (S = 3/2)

```
Energy              = -2924.054424
Enthalpy 0K         = -2923.535028
Free Energy 298K    = -2923.610196
```

```
C  9.15638  3.10912  6.72533
H  9.94696  2.40911  6.99572
C  9.19419  3.76114  5.49736
H 10.00641  3.56857  4.79437
C  8.16665  4.65499  5.20639
H  8.13850  5.18829  4.25330
C  7.17221  4.87458  6.16053
H  6.38340  5.58692  5.93515
C  7.20616  4.18874  7.38405
C  6.15326  4.45819  8.49864
C  5.19441  5.57407  8.03676
H  4.44608  5.80210  8.80523
H  4.65709  5.28626  7.12557
H  5.74172  6.49958  7.82074
C  6.89683  4.92952  9.77328
C  6.65942  6.18745 10.36628
H  5.90398  6.86946  9.97642
C  8.27278  5.82738 11.92949
H  8.84381  6.18544 12.79240
C  8.51997  4.57253 11.37323
H  9.29436  3.92462 11.78457
C  5.34226  3.16403  8.79891
C  3.95498  3.13551  8.61297
H  3.40874  4.00556  8.26178
C  3.25678  1.96382  8.88176
H  2.17420  1.92285  8.74121
C  3.94872  0.84432  9.32879
H  3.39616 -0.06738  9.53309
C  5.33662  0.91774  9.50277
C  8.55305  1.21839 12.40054
H  9.34432  1.96337 12.30850
C  8.34581  0.57210 13.61431
H  8.96082  0.81556 14.48234
C  6.60533 -0.66901 12.51961
H  5.83192 -1.43068 12.56685
C  6.87915  0.01591 11.32541
C  6.13932 -0.31982  9.99911
C  5.17076 -1.49308 10.24604
H  4.64091 -1.77561  9.32869
H  4.41655 -1.23605 10.99912
H  5.70971 -2.37810 10.60470
C  7.19501 -0.74374  8.93551
C  7.16787 -2.01410  8.33961
H  6.38235 -2.72799  8.57085
C  8.16408 -2.38729  7.43652
H  8.14048 -3.37648  6.97332
C  9.18675 -1.48646  7.15032
H  9.99928 -1.73552  6.46570
C  9.14260 -0.24126  7.76792
H  9.92852  0.48967  7.57737
N  8.18742  3.29783  7.63608
N  7.82750  4.12524 10.31707
N  5.99543  2.06687  9.23925
N  7.83230  0.96855 11.29491
N  8.17288  0.13056  8.61961
Co 8.11320  2.12632  9.47878
C  7.33506 -0.38442 13.67424
H  7.11990 -0.91686 14.60361
N  7.33275  6.62179 11.42789
C 11.43830  2.26385  9.85682
N 10.29064  2.21329  9.71614
C 12.88115  2.32561 10.03025
H 13.33462  2.90134  9.20855
H 13.12411  2.81860 10.98436
H 13.30410  1.30910 10.03595
```

2-Zn (S = 0)

```
Energy              = -3264.355648
Enthalpy 0K         = -3263.858693
Free Energy 298K    = -3263.926790
```

```
C  4.07772  5.32198  6.14948
H  4.88991  4.59308  6.16290
C  3.79093  6.04180  4.99491
H  4.36451  5.87585  4.08141
C  2.75325  6.96980  5.05387
H  2.47310  7.55343  4.17373
```

```
C  2.08265  7.16488  6.26270
H  1.29198  7.90900  6.30290
C  2.43830  6.41729  7.39599
C  1.78322  6.67286  8.78854
C  0.83458  7.88314  8.67830
H  1.36992  8.77460  8.33018
H  0.38112  8.12422  9.64693
H  0.01942  7.68736  7.97155
C  2.90812  6.99742  9.82069
C  2.96336  8.23286 10.48336
H  2.21260  8.99895 10.31170
C  3.99785  8.50358 11.38082
H  4.03794  9.46714 11.89426
C  4.97159  7.53334 11.60348
H  5.80360  7.69825 12.29014
C  4.85247  6.32927 10.91718
H  5.59327  5.54262 11.06431
C  0.96797  5.43038  9.26355
C -0.39705  5.52754  9.56231
H -0.93924  6.46344  9.46628
C -1.07959  4.39546  9.99608
H -2.14391  4.45499 10.23532
C -0.40207  3.18667 10.12229
H -0.94658  2.31001 10.46039
C  0.96133  3.13428  9.80800
C  5.03421  2.83520 11.42674
H  5.83866  3.49644 11.10052
C  5.16396  2.08626 12.59120
H  6.06529  2.16390 13.20158
C  4.10945  1.24513 12.93976
H  4.15079  0.64222 13.85000
C  2.99912  1.16239 12.09744
H  2.19371  0.48379 12.36479
C  2.94201  1.93522 10.92773
C  1.76458  1.80155  9.91377
C  0.81996  0.67626 10.38260
H  1.35328 -0.27807 10.46988
H -0.00833  0.53177  9.67876
H  0.38511  0.90514 11.36257
C  2.34901  1.42261  8.52402
C  2.00131  0.22528  7.86406
H  1.28193 -0.47453  8.28880
C  3.40885  0.68950  6.13537
H  3.84885  0.39817  5.17596
C  3.76404  1.88707  6.75796
H  4.48712  2.56084  6.29720
N  3.40926  5.48386  7.30412
N  3.85433  6.06308 10.05811
N  1.60116  4.24683  9.39447
N  3.95129  2.78231 10.63206
N  3.23352  2.24859  7.93337
N  2.52221 -0.12878  6.69197
O  5.88843  4.03555  8.52955
Zn 3.67265  4.16450  8.99263
H  6.47106  4.78185  8.32372
H  6.44370  3.24178  8.51466
```

3-Co (S = 3/2)

```
Energy              = -2883.761320
Enthalpy 0K         = -2883.275898
Free Energy 298K    = -2883.342626
```

```
C  3.95357  7.74958  3.20156
H  3.41594  6.91376  2.75159
C  3.28553  8.63900  4.03592
H  2.22508  8.50189  4.25446
C  4.01723  9.69525  4.57428
H  3.54776 10.41867  5.24502
C  5.36398  9.82878  4.23351
H  5.92369 10.66520  4.64317
C  5.96947  8.90064  3.37258
C  7.44857  9.05289  2.91117
C  8.03545 10.34496  3.51185
H  8.02043 10.31907  4.60789
H  7.46081 11.22248  3.19205
H  9.07512 10.49592  3.19809
C  7.47084  9.15526  1.35771
C  8.00451 10.27255  0.69737
H  8.46295 11.08776  1.25050
C  7.94682 10.35897 -0.69441
H  8.36349 11.22934 -1.20673
C  7.33938  9.32991 -1.41072
H  7.24928  9.35942 -2.49798
C  6.84545  8.24687 -0.69203
H  6.35325  7.42406 -1.21250
C  8.29263  7.83335  3.38601
C  9.39445  8.00889  4.23254
H  9.69333  8.99249  4.58177
C 10.12722  6.89895  4.63859
H 10.98923  7.02015  5.29878
C  9.75503  5.63287  4.19824
H 10.33229  4.77108  4.51959
C  8.64758  5.50310  3.35328
C  7.40693  4.78393 -0.74119
H  6.71849  5.42878 -1.28842
C  8.23257  3.88518 -1.41747
H  8.21017  3.80887 -2.50955
C  9.07705  3.18050  0.57596
H  9.77176  2.51391  1.08740
C  8.24138  4.06572  1.28797
C  8.19941  4.10379  2.83766
C  9.13837  3.01534  3.39742
H  8.84275  2.01922  3.04550
H  9.12138  2.99867  4.49365
H 10.17565  3.18352  3.08426
C  6.74611  3.80532  3.28941
C  6.42376  2.71653  4.12590
H  7.19396  2.06273  4.53532
C  4.19966  3.18029  3.98029
H  3.16664  2.92693  4.24042
C  4.48926  4.27536  3.16550
H  3.68969  4.90076  2.76697
N  5.25665  7.86154  2.89026
N  6.92235  8.14843  0.64651
N  7.94930  6.59447  2.97090
N  7.42444  4.88150  0.59579
N  9.07356  3.10028 -0.75177
N  5.75050  4.59098  2.83821
N  5.17333  2.41787  4.46720
O  4.54983  6.12414  0.38098
Co 6.27806  6.37733  1.70045
H  4.20094  5.29813  0.01317
H  3.98430  6.84018  0.05380
```

3'-Co (S = 1/2)

```
Energy              = -2940.057569
Enthalpy 0K         = -2939.548713
Free Energy 298K    = -2939.621933
```

```
C  4.02205  7.67581  3.08208
H  3.48751  6.89853  2.54187
C  3.33086  8.52981  3.93346
H  2.25752  8.40222  4.08354
C  4.04875  9.53776  4.57104
H  3.56058 10.22641  5.26445
C  5.40586  9.67671  4.28114
H  5.96001 10.48824  4.74475
C  6.03777  8.79369  3.39460
C  7.51092  9.00676  2.95540
C  8.04410 10.33306  3.52604
H  8.02951 10.32631  4.62254
H  7.44335 11.18574  3.18555
H  9.08215 10.50755  3.21860
C  7.52719  9.05107  1.40426
C  8.08373 10.13469  0.71079
H  8.58996 10.93421  1.24479
C  7.98342 10.21255 -0.67812
H  8.42107 11.05720 -1.21515
C  7.28782  9.21340 -1.35320
H  7.13514  9.24537 -2.43326
C  6.77897  8.15612 -0.60808
H  6.20212  7.37540 -1.09747
C  8.38300  7.83514  3.45533
C  9.48200  8.01643  4.30413
H  9.77761  9.00139  4.65597
C 10.21264  6.90086  4.70523
H 11.07414  7.01615  5.36698
C  9.83715  5.63657  4.25687
H 10.40790  4.76626  4.57060
C  8.72839  5.52710  3.41062
C  7.34145  4.94446 -0.67082
H  6.61271  5.56223 -1.18774
C  8.16299  4.07343 -1.38561
H  8.09858  4.01340 -2.47689
C  9.07718  3.35201  0.56175
H  9.79350  2.68896  1.04721
C  8.24692  4.20479  1.31591
C  8.23108  4.17209  2.86023
C  9.12444  3.02796  3.37567
H  8.79039  2.05366  2.99646
H  9.11590  2.98293  4.47140
H 10.16656  3.17011  3.06514
C  6.76755  3.94326  3.29873
C  6.40235  2.87740  4.14492
H  7.15525  2.21964  4.57907
C  4.19889  3.37356  3.92982
H  3.15297  3.14079  4.15465
C  4.52712  4.44950  3.10565
H  3.74635  5.05961  2.66057
N  5.34364  7.76713  2.84726
N  6.92776  8.04433  0.72432
N  8.03618  6.61129  3.03370
N  7.40271  5.03660  0.66662
N  9.04513  3.29749 -0.76601
N  5.80142  4.75538  2.81620
N  5.14120  2.60585  4.46540
Co 6.32802  6.39716  1.73398
C  3.55002  5.89723 -0.41269
N  4.45277  6.07318  0.29263
C  2.41538  5.67839 -1.29908
H  1.84323  6.61140 -1.41799
H  2.76709  5.34714 -2.28833
H  1.75409  4.90307 -0.88243
```

3'-Co (S = 3/2)

```
Energy             = -2940.061515
Enthalpy 0K        = -2939.554100
Free Energy 298K   = -2939.628568

C  3.95717  7.76088  3.20975
H  3.41669  6.92517  2.76336
C  3.29060  8.65366  4.04175
H  2.23078  8.51697  4.26335
C  4.02203  9.71362  4.57247
H  3.55339 10.44116  5.23925
C  5.36772  9.84529  4.22826
H  5.92784 10.68486  4.63067
C  5.97208  8.91064  3.37326
C  7.45039  9.05592  2.91256
C  8.04140 10.34641  3.51296
H  8.02237 10.32227  4.60892
H  7.47125 11.22579  3.19044
H  9.08262 10.49270  3.20225
C  7.48034  9.15526  1.35886
C  8.01537 10.27473  0.70322
H  8.46406 11.09265  1.26001
C  7.97330 10.35946 -0.68909
H  8.39201 11.23118 -1.19742
C  7.38014  9.32616 -1.41040
H  7.30456  9.35268 -2.49878
C  6.88138  8.24285 -0.69539
H  6.39942  7.41770 -1.22026
C  8.28536  7.83276  3.39163
C  9.38373  8.00920  4.24305
H  9.68024  8.99310  4.59294
C 10.11666  6.90113  4.65165
H 10.97607  7.02255  5.31508
C  9.74794  5.63671  4.20582
H 10.32625  4.77516  4.52536
C  8.64315  5.50682  3.35676
C  7.41859  4.77766 -0.74261
H  6.72632  5.41612 -1.29161
C  8.25024  3.88223 -1.41560
H  8.23151  3.80460 -2.50763
C  9.09172  3.18423  0.58042
H  9.78726  2.52098  1.09475
C  8.25097  4.06801  1.28843
C  8.20704  4.10551  2.83830
C  9.15188  3.02193  3.39779
H  8.86353  2.02494  3.04259
H  9.13142  3.00205  4.49389
H 10.18890  3.19720  3.08785
C  6.75762  3.80032  3.29251
C  6.43917  2.70106  4.11669
H  7.21072  2.03787  4.50793
C  4.21725  3.17764  4.00098
H  3.18585  2.92781  4.27064
C  4.50340  4.27911  3.19407
H  3.70153  4.90953  2.80891
N  5.25951  7.86958  2.89771
N  6.94252  8.14598  0.64300
N  7.93976  6.59517  2.97484
N  7.43288  4.87907  0.59296
N  9.09294  3.10166 -0.74723
N  5.76190  4.59375  2.85815
N  5.19122  2.40295  4.46722
Co  6.24993  6.37578  1.68725
C  3.63085  5.97335 -0.35388
```

```
N  4.54672  6.10768  0.34103
C  2.48093  5.80546 -1.22829
H  1.69075  6.52093 -0.95243
H  2.77539  5.98770 -2.27362
H  2.08749  4.78069 -1.14230
```

3-Zn (S = 0)

```
Energy              = -3280.361472
Enthalpy 0K         = -3279.877021
Free Energy 298K    = -3279.945654
```

```
C  6.93515  3.28673 14.26643
H  7.25447  2.82851 15.20403
C  5.82305  4.12061 14.23336
H  5.26677  4.33873 15.14660
C  5.45566  4.65808 13.00154
H  4.59791  5.32951 12.91664
C  6.18771  4.31381 11.86366
H  5.87869  4.72136 10.90520
C  7.29047  3.45217 11.96682
C  8.08213  2.97736 10.70825
C  7.40240  3.54681  9.44689
H  6.35844  3.21817  9.37882
H  7.91518  3.21843  8.53519
H  7.40664  4.64315  9.45176
C  8.04161  1.41806 10.64694
C  7.47834  0.73867  9.55605
H  7.08089  1.27723  8.70027
C  7.41302 -0.65591  9.55428
H  6.97403 -1.18133  8.70284
C  7.90255 -1.35881 10.65264
H  7.85977 -2.44800 10.70586
C  8.45637 -0.62293 11.69470
H  8.84500 -1.13117 12.57815
C  9.55875  3.47827 10.75964
C 10.07978  4.30593  9.75669
H  9.47749  4.63315  8.91465
C 11.40482  4.72187  9.83756
H 11.82581  5.36641  9.06236
C 12.19188  4.31334 10.91006
H 13.22512  4.64380 10.96256
C 11.62871  3.48943 11.89121
C 11.47882 -0.56739 13.52825
H 10.55608 -1.10794 13.74346
C 12.69937 -1.23595 13.42634
H 12.76266 -2.32204 13.55025
C 13.74350  0.75450 13.05185
H 14.68641  1.26480 12.85571
C 12.52883  1.46187 13.17123
C 12.46260  3.01307 13.11951
C 13.89699  3.57274 13.01592
H 14.39230  3.23191 12.09906
H 14.51102  3.24953 13.86562
H 13.89740  4.66905 13.00365
C 11.81111  3.52444 14.43460
C 12.48330  4.39631 15.31613
H 13.48057  4.77663 15.09530
C 10.74093  4.36439 16.78283
H 10.30544  4.68977 17.73334
C 10.04587  3.50970 15.92566
H  9.05106  3.14610 16.18537
N  7.65952  2.97917 13.17677
N  8.53827  0.71837 11.69028
N 10.34180  3.09780 11.79044
N 11.39760  0.76222 13.38150
N 13.81962 -0.56853 13.16921
N 10.57360  3.10555 14.76244
N 11.95205  4.80860 16.46422
O  8.65631  0.52279 14.88883
Zn  9.49496  1.85454 13.27582
H  7.76118  0.15172 14.89864
H  9.08212  0.23488 15.71018
```

1'-Co+e^{-} (S = 0)

```
Energy              = -2924.288386
Enthalpy 0K         = -2923.772340
Free Energy 298K    = -2923.849517
```

```
C  7.51836 -2.22613  1.18595
H  8.17337 -3.04351  0.89435
C  8.04282 -1.01659  1.67007
C 10.47345  2.95126 -0.01061
H 10.72882  3.89519 -0.49325
C  7.99751  4.44324  1.92144
C  9.89742  0.59341  1.14918
C  9.44188 -0.09921  5.43382
H  8.76135  0.47407  6.06668
C  5.89834 -0.18185  1.93818
H  5.27883  0.65582  2.26357
C  5.86920  3.55029  2.12460
H  5.26646  2.67667  2.37895
C  5.30327 -1.34181  1.45075
H  4.21689 -1.41431  1.37398
C 10.33568 -1.89341  1.04685
H  9.98237 -2.00530  0.01471
H 10.20437 -2.86078  1.54859
H 11.41118 -1.67927  1.00676
C  9.93552  4.09978  3.49985
C 10.45329  3.94069  6.21265
H 10.59891  3.86723  7.29208
C  9.96785 -0.77765  3.28213
C  9.53287  4.22187  2.01045
C  9.87889  2.92221  1.25327
C 11.30592 -1.55971  5.14307
H 12.14068 -2.14785  5.53145
C  9.42459  3.23952  5.58888
H  8.75039  2.61108  6.17492
C  6.13848 -2.39198  1.07390
H  5.72648 -3.33001  0.69440
C 10.49131  0.68666 -0.11179
H 10.76098 -0.20648 -0.67630
C  9.57356 -0.77504  1.78625
C 10.47122 -0.84539  6.00154
H 10.61122 -0.86194  7.08407
C 10.27075  5.41582  1.37152
H 11.34930  5.22496  1.30577
H 10.12695  6.33084  1.96044
H  9.90838  5.61506  0.35581
C  5.25126  4.73757  1.74322
H  4.16333  4.79721  1.67829
C  6.06588  5.83117  1.45537
H  5.63554  6.79125  1.16091
C  7.44893  5.68064  1.54696
H  8.08796  6.53195  1.32557
C 11.28032  4.72864  5.41310
H 12.11457  5.28599  5.84553
C 11.01289  4.81537  4.04627
H 11.64188  5.44760  3.42374
```

```
C 11.04523 -1.53222  3.77254
H 11.67939 -2.10773  3.10215
N  9.21182 -0.03879  4.11531
N  7.22409 -0.01744  2.04902
N  9.55959  1.72731  1.82613
N 10.77323  1.84987 -0.70111
N  9.18795  3.28969  4.27143
N  7.19836  3.39978  2.21439
Co  8.08203  1.65229  3.16973
C  5.85767  1.51640  5.42558
N  6.64321  1.57137  4.57480
C  4.86855  1.44628  6.49357
H  5.07924  2.20790  7.25999
H  3.85984  1.62526  6.09054
H  4.88712  0.45218  6.96605
```

1'-Co+e$^-$ (S = 0, 5-coordinates)

```
Energy             = -2791.618195
Enthalpy 0K        = -2791.147074
Free Energy 298K   = -2791.211067
```

```
N  7.28874  3.26612  2.60774
C  7.94404  4.22102  1.90955
C  7.22966  5.24102  1.26116
C  5.84115  5.30689  1.37412
C  5.18723  4.36134  2.16245
C  5.95539  3.36639  2.75684
C  9.49902  4.19313  1.92058
C 10.04877  5.43580  1.19661
Co  8.37953  1.65188  3.26918
N  9.50978  3.19917  4.20839
C  9.95227  4.19579  3.40894
C 10.77452  5.21176  3.92185
C 11.12860  5.21343  5.27026
C 10.64313  4.19695  6.08987
C  9.84052  3.21968  5.51025
C 10.04881  2.92221  1.22693
C 10.93498  2.95319  0.13853
N 11.41683  1.83988 -0.40971
C 11.02809  0.67269  0.09899
C 10.13084  0.59340  1.17578
N  9.66797  1.73177  1.71299
C  9.62027 -0.74305  1.76917
C  9.92800 -0.84516  3.28996
C 10.75435 -1.85467  3.80856
C 10.91095 -1.99471  5.18724
C 10.21109 -1.13473  6.03202
C  9.42857 -0.14407  5.44911
N  9.31635  0.03019  4.11967
C  8.07505 -0.81547  1.60256
C  7.46841 -1.82314  0.83557
C  6.07929 -1.89015  0.73885
C  5.31565 -0.94720  1.42318
C  5.98586  0.02625  2.15697
N  7.32402  0.10990  2.24117
C 10.28907 -1.91077  1.02177
H  8.06611 -2.56429  0.31126
H 11.27140  3.88911 -0.30919
H  8.84486  0.53358  6.07470
H  5.41773  0.77351  2.70959
H  5.48532  2.62198  3.40199
H  4.22462 -0.96023  1.39731
H 10.05899 -1.87948 -0.05025
H  9.94890 -2.87897  1.40951
H 11.38096 -1.87214  1.12416
H 10.87347  4.15623  7.15592
H 11.55368 -2.78057  5.59029
H  9.43888  2.41519  6.12508
H  5.60650 -2.67451  0.14345
H 11.44862 -0.21636 -0.37259
H 10.26192 -1.22654  7.11840
H 11.14527  5.45514  1.21851
H  9.68834  6.36159  1.66198
H  9.74182  5.44742  0.14308
H  4.10781  4.39066  2.32125
H  5.28574  6.10085  0.86987
H  7.74311  5.99645  0.67211
H 11.76703  6.00439  5.67031
H 11.14342  6.00990  3.28302
H 11.27464 -2.54536  3.14985
```

1'-Co+e$^-$ (S = 1)

```
Energy             = -2924.316085
Enthalpy 0K        = -2923.799349
Free Energy 298K   = -2923.876237
```

```
C  7.41994 -1.89738  0.79875
H  8.02731 -2.61782  0.25743
C  8.00857 -0.89275  1.58666
C 10.92878  2.94575  0.26740
H 11.30524  3.88308 -0.14072
C  7.95152  4.32535  1.82062
C 10.04098  0.59088  1.22686
C  9.61505 -0.20744  5.43775
H  9.12032  0.50602  6.09908
C  5.90551 -0.10587  2.16145
H  5.33237  0.62065  2.74011
C  5.86538  3.45022  2.32620
H  5.30782  2.66409  2.83847
C  5.24943 -1.07585  1.40976
H  4.15872 -1.11081  1.38289
C 10.22956 -1.91057  0.95057
H  9.98715 -1.84260 -0.11671
H  9.90220 -2.89579  1.30508
H 11.32128 -1.86740  1.04492
C  9.85895  4.25731  3.48671
C 10.37789  4.29255  6.20833
H 10.53991  4.26875  7.28757
C  9.91270 -0.93633  3.25769
C  9.49676  4.23179  1.97456
C 10.01482  2.92073  1.32888
C 11.02444 -2.09535  5.07608
H 11.68079 -2.89308  5.43162
C  9.57776  3.33391  5.59403
H  9.09572  2.55739  6.19055
C  6.03170 -1.98756  0.70367
H  5.57318 -2.76754  0.09134
C 10.95403  0.67935  0.16804
H 11.35052 -0.21007 -0.32039
C  9.55123 -0.78307  1.75301
C 10.43125 -1.20270  5.96659
H 10.59286 -1.27132  7.04397
C 10.14990  5.43981  1.27543
H 11.24246  5.41023  1.36451
H  9.80400  6.38257  1.71702
H  9.90571  5.46241  0.20662
C  5.18857  4.47162  1.66658
```

```
H  4.09731  4.48843  1.64534
C  5.95115  5.45812  1.04461
H  5.47604  6.28177  0.50661
C  7.34117  5.38594  1.12835
H  7.93276  6.16437  0.65387
C 10.95452  5.26999  5.39987
H 11.59807  6.04422  5.82411
C 10.68673  5.25435  4.03146
H 11.12621  6.02663  3.40577
C 10.75712 -1.96353  3.71387
H 11.21020 -2.66946  3.02313
N  9.37398 -0.06108  4.12705
N  7.23991 -0.00062  2.23889
N  9.57414  1.72788  1.80125
N 11.39560  1.84076 -0.31044
N  9.33728  3.29903  4.27548
N  7.20172  3.36365  2.39104
Co  8.22086  1.64599  3.33171
C  6.06802  1.50428  5.76731
N  6.83430  1.55657  4.89736
C  5.10524  1.43856  6.85961
H  5.62458  1.29145  7.81923
H  4.52558  2.37286  6.91442
H  4.41025  0.59869  6.70610
```

1'-Co+e⁻ (S = 1, 5-coordinates)

```
Energy              = -2791.640328
Enthalpy 0K         = -2791.169446
Free Energy 298K    = -2791.233758

N  7.24128  3.30918  2.50589
C  7.93621  4.26491  1.85201
C  7.25928  5.30350  1.19231
C  5.86750  5.38146  1.24928
C  5.17002  4.42653  1.98653
C  5.90421  3.41245  2.59261
C  9.49205  4.21292  1.91552
C 10.07705  5.44260  1.19574
Co  8.33955  1.65169  3.20772
N  9.45253  3.24420  4.21272
C  9.90364  4.23736  3.41808
C 10.70027  5.26615  3.94630
C 11.01480  5.27961  5.30506
C 10.52029  4.26277  6.11874
C  9.74700  3.27166  5.52177
C 10.04689  2.92818  1.25120
C 10.96517  2.95445  0.19312
N 11.45903  1.84420 -0.35293
C 11.04490  0.67805  0.14043
C 10.12081  0.59101  1.19010
N  9.63801  1.73230  1.72932
C  9.60811 -0.75944  1.74974
C  9.87955 -0.88596  3.27845
C 10.68322 -1.91122  3.80258
C 10.81405 -2.06404  5.18308
C 10.11851 -1.19799  6.02454
C  9.36216 -0.19017  5.43538
N  9.26720 -0.01238  4.10662
C  8.06814 -0.85713  1.53401
C  7.49713 -1.87865  0.75762
C  6.11177 -1.95943  0.61805
C  5.31568 -1.01673  1.26467
C  5.95362 -0.02826  2.00808
N  7.28645  0.06377  2.13581
C 10.31095 -1.91054  1.00640
H  8.11685 -2.61985  0.25956
H 11.32134  3.89141 -0.23570
H  8.79370  0.50396  6.05859
H  5.36236  0.72694  2.52755
H  5.39716  2.64962  3.18794
H  4.22645 -1.03993  1.19930
H 10.10859 -1.86981 -0.07068
H  9.96988 -2.88627  1.37348
H 11.39895 -1.86272  1.13980
H 10.72466  4.23112  7.19040
H 11.43842 -2.86280  5.58982
H  9.34366  2.45937  6.12775
H  5.66621 -2.75473  0.01608
H 11.47108 -0.21074 -0.32556
H 10.15791 -1.29418  7.11101
H 11.17219  5.45250  1.25338
H  9.71133  6.37587  1.64125
H  9.80249  5.44731  0.13346
H  4.08421  4.45968  2.09211
H  5.34193  6.19047  0.73686
H  7.80234  6.06404  0.63752
H 11.63260  6.08060  5.71753
H 11.07950  6.06488  3.31432
H 11.20624 -2.60417  3.14865
```

2'-Co+e⁻ (S = 0)

```
Energy              = -2924.285843
Enthalpy 0K         = -2923.769738
Free Energy 298K    = -2923.847193

C  8.98104  3.18256  6.49856
H  9.62709  2.30401  6.55638
C  9.14962  4.10823  5.47196
H  9.91693  3.95549  4.71069
C  8.31366  5.22327  5.45757
H  8.40662  5.98614  4.68101
C  7.34677  5.35188  6.45473
H  6.69490  6.22222  6.44739
C  7.23087  4.36444  7.44656
C  6.16674  4.45390  8.57343
C  5.17095  5.59159  8.26539
H  4.33593  5.59479  8.97731
H  4.74782  5.49177  7.25835
H  5.66156  6.57168  8.32175
C  6.88031  4.75010  9.91035
C  6.43119  5.73629 10.80737
H  5.51052  6.29251 10.62335
C  8.23211  5.41208 12.15079
H  8.81021  5.69553 13.03710
C  8.68176  4.40824 11.29780
H  9.62034  3.88518 11.48955
C  5.41113  3.10096  8.65923
C  4.11392  2.97495  8.14670
H  3.60844  3.81252  7.67335
C  3.46048  1.75142  8.24433
H  2.45157  1.62826  7.84428
C  4.10387  0.69063  8.87316
H  3.58708 -0.26031  8.96842
C  5.40285  0.86866  9.36620
C  8.65667  1.27448 12.31740
H  9.57348  1.84072 12.14156
C  8.28554  0.91741 13.61099
H  8.90374  1.20470 14.46363
```

```
C  6.38915 -0.18615 12.63529
H  5.48334 -0.77522 12.75761
C  6.84817  0.18731 11.36147
C  6.15140 -0.29924 10.06248
C  5.15156 -1.42004 10.41315
H  4.73704 -1.88613  9.51110
H  4.31106 -1.03223 11.00246
H  5.63480 -2.21088 11.00054
C  7.23098 -0.84881  9.08979
C  7.34028 -2.21971  8.80330
H  6.67577 -2.94590  9.26512
C  8.31524 -2.66918  7.91312
H  8.40187 -3.73405  7.68474
C  9.16817 -1.73703  7.32502
H  9.94361 -2.03368  6.61632
C  9.00693 -0.39890  7.67305
H  9.66817  0.36140  7.25296
N  8.05596  3.30167  7.46024
N  7.99178  4.04526 10.20439
N  6.04027  2.05011  9.22579
N  7.94527  0.95426 11.22776
N  8.07229  0.03874  8.52822
Co  8.14762  2.08390  9.33541
C  7.10621  0.19193 13.77102
H  6.75330 -0.09597 14.76405
N  7.08846  6.05844 11.92173
C 11.33819  2.14563  9.45810
N 10.18025  2.10070  9.38886
C 12.79248  2.20292  9.54290
H 13.15974  1.50100 10.30743
H 13.24316  1.93318  8.57527
H 13.12071  3.21940  9.80962
```

2'-Co+e$^-$ (S = 0, 5-coordinates)

```
Energy             = -2791.617500
Enthalpy 0K        = -2791.146291
Free Energy 298K   = -2791.209647

N  7.43602  6.39814 11.60474
C  6.69339  6.00180 10.57111
C  6.93928  4.82095  9.84907
N  7.94863  4.00541 10.24310
C  8.74104  4.44355 11.24368
C  8.47921  5.62900 11.92074
C  6.14412  4.44267  8.57654
C  5.28771  3.16388  8.80095
C  3.91156  3.12648  8.54117
C  3.22396  1.93055  8.73384
C  3.90974  0.80459  9.18395
C  5.28434  0.90490  9.43658
N  5.92440  2.06680  9.23711
C  6.12586 -0.28982  9.96811
C  7.28271 -0.65144  8.99334
C  7.36022 -1.90538  8.36673
C  8.47756 -2.24162  7.60290
C  9.52234 -1.32405  7.50189
C  9.36959 -0.09325  8.12990
N  8.26833  0.25715  8.81988
Co  7.95930  2.14471  9.51617
N  7.67237  1.10671 11.34458
C  8.28644  1.40818 12.50096
C  8.02846  0.76308 13.70593
C  7.08015 -0.25704 13.71015
C  6.45387 -0.59195 12.51055
```

```
C  6.77072  0.09821 11.32947
C  7.17513  4.16686  7.44859
C  7.19797  4.93063  6.27062
C  8.15406  4.67641  5.28875
C  9.08060  3.65879  5.50588
C  8.99792  2.94466  6.69668
N  8.07539  3.17694  7.64503
C  5.22306  5.61155  8.18031
C  5.20858 -1.51249 10.15164
H  9.71115  2.14655  6.89802
H  9.85564  3.41918  4.77580
H  8.17283  5.27021  4.37208
H  6.47779  5.72799  6.10653
H  4.46480  5.79663  8.95136
H  4.69426  5.40014  7.24285
H  5.79242  6.53907  8.04159
H  5.86417  6.65967 10.30771
H  9.13512  5.96340 12.73155
H  9.61858  3.84200 11.48638
H  3.36986  4.00188  8.19215
H  2.15149  1.87576  8.53350
H  3.36500 -0.12429  9.33085
H  9.02674  2.20578 12.46370
H  8.56293  1.05802 14.61063
H  5.72188 -1.39523 12.50968
H  4.74857 -1.80745  9.20029
H  4.39586 -1.29762 10.85617
H  5.76614 -2.37557 10.53598
H  6.56173 -2.63492  8.47341
H  8.53636 -3.21752  7.11578
H 10.43959 -1.55249  6.95635
H 10.17410  0.64394  8.10122
H  6.83335 -0.79451 14.62853
```

2'-Co+e$^-$ (S = 1)

```
Energy             = -2924.313407
Enthalpy 0K        = -2923.796819
Free Energy 298K   = -2923.873539

C  9.07344  3.15985  6.60761
H  9.80359  2.36670  6.77749
C  9.15739  3.95409  5.46824
H  9.94280  3.77980  4.73046
C  8.21412  4.96790  5.31167
H  8.23126  5.62361  4.43804
C  7.24296  5.13892  6.29759
H  6.51443  5.93664  6.17925
C  7.22094  4.29124  7.41820
C  6.16745  4.45777  8.54991
C  5.18974  5.59072  8.17739
H  4.39657  5.69657  8.92796
H  4.70875  5.40166  7.21044
H  5.71086  6.55323  8.10351
C  6.89891  4.83435  9.86219
C  6.56755  5.98561 10.60287
H  5.73404  6.62590 10.31308
C  8.26950  5.61679 12.05715
H  8.84984  5.93370 12.93048
C  8.60517  4.46048 11.36060
H  9.45629  3.85096 11.66630
C  5.38132  3.12704  8.72377
C  4.03078  3.04634  8.35801
H  3.49976  3.90076  7.94857
C  3.35307  1.84347  8.51862
```

```
H  2.30264  1.75703  8.23186
C  4.02746  0.75448  9.05932
H  3.49260 -0.18086  9.19444
C  5.37677  0.88709  9.41403
C  8.62963  1.22009 12.35484
H  9.50840  1.85105 12.20929
C  8.31268  0.73871 13.62176
H  8.93202  0.99658 14.48290
C  6.46535 -0.39848 12.59445
H  5.60160 -1.05152 12.68844
C  6.86501  0.10759 11.34567
C  6.15348 -0.31275 10.02772
C  5.16792 -1.45799 10.33534
H  4.70782 -1.84829  9.41969
H  4.35887 -1.12066 10.99473
H  5.67731 -2.29306 10.83200
C  7.22215 -0.81425  9.01491
C  7.26695 -2.15465  8.59380
H  6.54922 -2.88477  8.95829
C  8.24739 -2.57234  7.69444
H  8.28226 -3.61333  7.36472
C  9.17596 -1.64134  7.23377
H  9.96656 -1.91490  6.53259
C  9.06982 -0.33490  7.70169
H  9.78843  0.42174  7.38206
N  8.13387  3.31041  7.55318
N  7.90901  4.04712 10.28850
N  6.02889  2.05306  9.22203
N  7.91324  0.94682 11.25488
N  8.12360  0.07494  8.55830
Co  8.15478  2.12257  9.40029
C  7.18561 -0.07157 13.74361
H  6.87570 -0.46292 14.71536
N  7.23267  6.37071 11.69169
C 11.37592  2.21607  9.64282
N 10.22096  2.15889  9.53643
C 12.82574  2.29011  9.77588
H 13.17788  1.57283 10.53325
H 13.31129  2.05229  8.81674
H 13.13096  3.30333 10.07989
```

2'-Co+e$^-$ (S = 1, 5-coordinates)

```
Energy              = -2791.638231
Enthalpy 0K         = -2791.167411
Free Energy 298K    = -2791.231507

N  7.59239  6.54930 11.43105
C  6.80978  6.11917 10.44138
C  6.97705  4.87382  9.80782
N  7.94210  4.03665 10.25604
C  8.76414  4.49386 11.22068
C  8.58993  5.74591 11.80037
C  6.14680  4.45542  8.56576
C  5.30704  3.17293  8.83061
C  3.92036  3.14771  8.63532
C  3.22706  1.95998  8.84949
C  3.92161  0.82538  9.25972
C  5.30644  0.90395  9.45546
N  5.95760  2.06369  9.23509
C  6.13284 -0.31750  9.95414
C  7.28535 -0.68568  8.97183
C  7.35044 -1.94120  8.34588
C  8.45939 -2.28459  7.57206
C  9.50599 -1.37186  7.45389
C  9.36227 -0.13762  8.07836
N  8.27431  0.21493  8.78553
Co  7.95971  2.14530  9.50812
N  7.65889  1.04107 11.38296
C  8.26100  1.32719 12.54731
C  8.01431  0.64096 13.73275
C  7.08861 -0.39942 13.70057
C  6.46805 -0.71039 12.49061
C  6.77541  0.02322 11.33293
C  7.14747  4.18239  7.40326
C  7.11375  4.92468  6.21147
C  8.04651  4.67301  5.20556
C  9.00455  3.68253  5.41062
C  8.97136  2.98580  6.61495
N  8.07145  3.21625  7.58310
C  5.20360  5.61036  8.17772
C  5.19620 -1.53026 10.11020
H  9.70401  2.20139  6.80905
H  9.76220  3.44954  4.66032
H  8.02250  5.24966  4.27803
H  6.37126  5.70230  6.05248
H  4.48156  5.81822  8.97734
H  4.63537  5.37156  7.27067
H  5.76325  6.53358  7.98458
H  6.01388  6.80214 10.14320
H  9.26967  6.09855 12.58332
H  9.58574  3.84385 11.52925
H  3.37257  4.03052  8.31765
H  2.14630  1.91828  8.69701
H  3.37310 -0.09802  9.42226
H  8.97774  2.14946 12.53536
H  8.53584  0.92053 14.64987
H  5.75118 -1.52695 12.46676
H  4.72479 -1.79247  9.15473
H  4.39439 -1.32445 10.82934
H  5.74337 -2.41125 10.46707
H  6.55000 -2.66745  8.45858
H  8.50768 -3.26188  7.08645
H 10.41261 -1.60336  6.89204
H 10.16194  0.60472  8.02051
H  6.85150 -0.96989 14.60154
```

3'-Co+e$^-$ (S = 0)

```
Energy              = -2940.300145
Enthalpy 0K         = -2939.796030
Free Energy 298K    = -2939.873759

C  3.98200  7.84154  3.37970
H  3.46370  6.90687  3.15671
C  3.29797  8.89537  3.97912
H  2.24543  8.78911  4.24819
C  4.00049 10.07574  4.21335
H  3.51339 10.93903  4.67273
C  5.34582 10.14034  3.85135
H  5.89296 11.06231  4.03129
C  5.96919  9.02634  3.26476
C  7.46898  9.04794  2.85728
C  8.13016 10.33560  3.38954
H  7.96914 10.45432  4.46781
H  7.71811 11.22402  2.89466
H  9.21345 10.32800  3.21534
C  7.57113  9.02632  1.30799
C  8.38368  9.93158  0.60551
H  9.02338 10.63862  1.12811
```

C 8.37112 9.93975 -0.78964
H 9.00187 10.64188 -1.33984
C 7.52571 9.05748 -1.45985
H 7.44993 9.04916 -2.54882
C 6.77316 8.17250 -0.69226
H 6.09419 7.46446 -1.17150
C 8.17354 7.80805 3.47215
C 9.04307 7.94780 4.56219
H 9.24938 8.91971 5.00168
C 9.65537 6.81926 5.09679
H 10.32872 6.90780 5.95228
C 9.40996 5.57890 4.51795
H 9.89942 4.69639 4.92128
C 8.53471 5.49556 3.42799
C 7.24613 4.89234 -0.73714
H 6.37916 5.34130 -1.22470
C 8.28019 4.33109 -1.48159
H 8.24751 4.33177 -2.57648
C 9.33381 3.73890 0.44053
H 10.19830 3.26188 0.90515
C 8.28664 4.26940 1.21643
C 8.23686 4.12927 2.75461
C 9.27357 3.07952 3.20657
H 9.15198 2.13949 2.65355
H 9.17279 2.84965 4.27445
H 10.29786 3.43524 3.03861
C 6.81612 3.67000 3.15919
C 6.55110 2.37962 3.65758
H 7.34628 1.64259 3.77737
C 4.34112 2.85729 3.86777
H 3.33306 2.54517 4.16118
C 4.57536 4.13467 3.36213
H 3.75572 4.84423 3.23351
N 5.27485 7.89762 3.03084
N 6.81798 8.12957 0.64626
N 7.91626 6.59290 2.94578
N 7.26476 4.90097 0.60516
N 9.34069 3.77918 -0.89149
N 5.80124 4.54015 3.00673
N 5.33109 1.97740 4.00916
Co 6.16709 6.32976 1.77036
C 3.50203 5.93210 0.04069
N 4.45634 6.08493 0.68356
C 2.30102 5.73878 -0.76278
H 1.50818 6.42933 -0.43637
H 2.51510 5.93036 -1.82555
H 1.93467 4.70568 -0.65992

C 10.13068 6.88959 4.73808
C 9.38839 8.00259 4.34902
C 8.32146 7.82762 3.45750
N 8.02032 6.60384 2.99878
C 7.45930 9.01399 2.94124
C 7.59379 9.10957 1.39432
C 8.13822 10.24687 0.77680
C 8.20717 10.32698 -0.61336
C 7.71729 9.26452 -1.36873
C 7.19809 8.16733 -0.68935
N 7.14549 8.07321 0.64963
C 5.95094 8.81067 3.26492
C 5.25041 9.68476 4.11104
C 3.87113 9.55254 4.27147
C 3.20341 8.56160 3.55345
C 3.96283 7.71589 2.75275
N 5.30128 7.80208 2.64210
C 7.95196 10.31854 3.59323
C 8.22821 4.16564 1.24215
C 9.07142 3.34908 0.46715
N 8.99763 3.27418 -0.86072
C 8.04313 3.99555 -1.45027
C 7.20376 4.82773 -0.71791
N 7.31982 4.95477 0.61951
H 3.47856 6.94368 2.15179
H 2.11945 8.44470 3.60365
H 3.32724 10.23326 4.93018
H 5.76462 10.48041 4.64373
H 7.86970 10.26906 4.68619
H 7.36693 11.18130 3.25130
H 9.00455 10.51078 3.35231
H 8.51007 11.08025 1.36683
H 8.63044 11.21245 -1.09297
H 7.73052 9.27745 -2.45992
H 6.80104 7.32358 -1.25145
H 9.65323 8.98064 4.74210
H 10.96940 7.00401 5.42854
H 10.38282 4.76619 4.54995
H 6.40272 5.38470 -1.20620
H 7.93237 3.90896 -2.53629
H 9.83824 2.72887 0.93312
H 8.88051 2.05301 2.88887
H 9.17084 2.96328 4.38183
H 10.21501 3.21580 2.97605
H 7.21735 2.04558 4.36340
H 3.23365 3.13349 4.34356
H 3.77656 5.13734 2.95106

3'-Co+e$^-$ (S = 0, 5-coordinates)

Energy = -2807.631215
Enthalpy 0K = -2807.171979
Free Energy 298K = -2807.235426

N 5.21357 2.48932 4.43007
C 6.45671 2.75631 4.03865
C 6.79495 3.87831 3.25713
N 5.82140 4.73849 2.87884
C 4.56633 4.45717 3.26608
C 4.26512 3.33960 4.03942
C 8.24428 4.14911 2.78963
C 9.17931 3.03080 3.28696
Co 6.42949 6.37906 1.71084
C 8.71475 5.51637 3.36383
C 9.79880 5.63025 4.24346

3'-Co+e$^-$ (S = 1)

Energy = -2940.328356
Enthalpy 0K = -2939.823426
Free Energy 298K = -2939.899840

C 3.95198 7.77134 3.22852
H 3.41222 6.92830 2.79417
C 3.29006 8.67286 4.05667
H 2.23231 8.53666 4.28932
C 4.02391 9.74071 4.56799
H 3.56115 10.47709 5.22914
C 5.36618 9.86583 4.21118
H 5.92990 10.70978 4.59932
C 5.96244 8.91664 3.36230
C 7.44281 9.05242 2.90034
C 8.03863 10.34131 3.49929

```
H  8.00842 10.32409  4.59523
H  7.47974 11.22381  3.16466
H  9.08475 10.47562  3.19929
C  7.48340  9.15074  1.34712
C  8.05585 10.25673  0.69439
H  8.52476 11.06199  1.25330
C  8.02416 10.34166 -0.69727
H  8.47079 11.19987 -1.20474
C  7.40199  9.32479 -1.41792
H  7.32937  9.35151 -2.50674
C  6.86813  8.25802 -0.70108
H  6.35955  7.44599 -1.22326
C  8.27176  7.82682  3.38547
C  9.35556  8.00317  4.25744
H  9.64371  8.98638  4.61643
C 10.08357  6.89561  4.67623
H 10.93075  7.01508  5.35530
C  9.72028  5.63477  4.21868
H 10.29050  4.77015  4.54476
C  8.62812  5.51026  3.34934
C  7.41527  4.80069 -0.75098
H  6.71481  5.43315 -1.29770
C  8.25788  3.92282 -1.42515
H  8.24205  3.85848 -2.51843
C  9.09301  3.21449  0.56246
H  9.78771  2.55205  1.07943
C  8.24393  4.08574  1.27323
C  8.19483  4.11240  2.82182
C  9.14200  3.02956  3.37668
H  8.86058  2.03447  3.01096
H  9.11566  2.99996  4.47259
H 10.17962  3.21428  3.07339
C  6.74536  3.80120  3.27315
C  6.43178  2.69187  4.08318
H  7.20893  2.02528  4.45770
C  4.21561  3.16926  3.98627
H  3.18415  2.92054  4.25812
C  4.49508  4.27497  3.18987
H  3.69116  4.90672  2.81019
N  5.24877  7.87194  2.90336
N  6.92405  8.15563  0.63438
N  7.92983  6.59494  2.95430
N  7.41888  4.90429  0.58807
N  9.11073  3.13751 -0.76716
N  5.75072  4.60739  2.84722
N  5.18808  2.38109  4.44474
Co  6.23819  6.32328  1.67148
C  3.66315  5.94799 -0.28228
N  4.57707  6.10192  0.41632
C  2.51493  5.75313 -1.15893
H  1.71144  6.45928 -0.89874
H  2.80211  5.92118 -2.20837
H  2.12984  4.72644 -1.06104
```

3'-Co+e⁻ (S = 1, 5-coordinates)

```
Energy             = -2807.651837
Enthalpy 0K        = -2807.192936
Free Energy 298K   = -2807.257052

N  5.27428  2.46420  4.57489
C  6.50495  2.74495  4.15103
C  6.80114  3.84948  3.32869
N  5.80328  4.67946  2.95688
C  4.56195  4.38468  3.37305
C  4.29865  3.27964  4.17892
C  8.23562  4.13331  2.80874
C  9.19322  3.03461  3.30939
Co  6.39932  6.36873  1.75422
N  7.30957  4.91729  0.62429
C  7.15649  4.75525 -0.70348
C  7.91392  3.83597 -1.42228
N  8.83164  3.07591 -0.82528
C  8.94915  3.19926  0.49622
C  8.17994  4.09821  1.25856
C  8.71052  5.51676  3.33671
C  9.83346  5.64528  4.16331
C 10.18764  6.90763  4.63083
C  9.42370  8.01307  4.26664
C  8.31558  7.83402  3.42832
N  7.99053  6.60169  2.98951
C  7.44419  9.03427  2.95647
C  7.53879  9.16557  1.40610
C  8.04038 10.32592  0.79429
C  8.07526 10.42408 -0.59682
C  7.60109  9.35820 -1.35759
C  7.12675  8.23852 -0.68047
N  7.10151  8.13287  0.65673
C  5.94165  8.84426  3.32658
C  5.27558  9.73089  4.18832
C  3.90204  9.60611  4.39963
C  3.20480  8.60718  3.72283
C  3.93364  7.74831  2.90688
N  5.26448  7.83482  2.73903
C  7.96574 10.32573  3.61406
H  3.42614  6.95703  2.34977
H  2.12426  8.48967  3.82196
H  3.38553 10.29663  5.07019
H  5.81012 10.52997  4.69497
H  7.92102 10.25947  4.70827
H  7.37202 11.19514  3.30656
H  9.00861 10.52087  3.33694
H  8.40392 11.16255  1.38492
H  8.46399 11.32686 -1.07353
H  7.59597  9.38547 -2.44864
H  6.74875  7.38420 -1.24348
H  9.70510  8.99410  4.63868
H 11.05795  7.03006  5.27937
H 10.43316  4.78586  4.44998
H  6.40363  5.36995 -1.20123
H  7.77552  3.72030 -2.50242
H  9.69078  2.55116  0.96433
H  8.88059  2.04359  2.95832
H  9.22469  3.00409  4.40527
H 10.21582  3.20825  2.95198
H  7.28508  2.05904  4.48262
H  3.27823  3.05693  4.50807
H  3.75409  5.04664  3.05999
```

1'-Co+2e⁻ (S = 1/2)

```
Energy             = -2924.423764
Enthalpy 0K        = -2923.912361
Free Energy 298K   = -2923.991039

C  7.48209 -2.09861  1.01702
H  8.12831 -2.87337  0.61171
C  8.02683 -0.97410  1.66801
C 10.54967  2.95124  0.04317
H 10.81749  3.89885 -0.42812
```

```
C  7.97641  4.40314  1.90219
C  9.92437  0.59273  1.16950
C  9.51492 -0.12372  5.47228
H  8.85194  0.44965  6.12487
C  5.88102 -0.17039  2.04748
H  5.27094  0.62034  2.49085
C  5.84587  3.53163  2.21304
H  5.25076  2.69503  2.58719
C  5.27237 -1.24834  1.41347
H  4.18424 -1.30726  1.34360
C 10.29462 -1.90765  1.06655
H  9.96495 -1.98219  0.02402
H 10.11442 -2.87845  1.54927
H 11.37589 -1.72603  1.04855
C  9.92578  4.11040  3.50943
C 10.50733  3.97584  6.21902
H 10.67800  3.90736  7.29567
C  9.97319 -0.78542  3.29617
C  9.51239  4.21617  2.01904
C  9.90280  2.92037  1.27040
C 11.36975 -1.57977  5.11777
H 12.21696 -2.17030  5.47573
C  9.47680  3.25731  5.62032
H  8.82327  2.61889  6.22013
C  6.10315 -2.24066  0.88472
H  5.68637 -3.11569  0.37953
C 10.57042  0.67999 -0.05550
H 10.85496 -0.21849 -0.60640
C  9.55904 -0.77021  1.80280
C 10.56044 -0.87120  6.00630
H 10.73341 -0.89099  7.08458
C 10.22702  5.42661  1.38582
H 11.31151  5.26718  1.35447
H 10.02903  6.34831  1.95085
H  9.89696  5.58609  0.35294
C  5.21685  4.65028  1.67718
H  4.12762  4.69611  1.61501
C  6.02857  5.69956  1.23613
H  5.59508  6.60813  0.81052
C  7.41021  5.57044  1.35321
H  8.04161  6.38890  1.01651
C 11.30479  4.77214  5.39641
H 12.14039  5.34547  5.80590
C 11.00546  4.84271  4.03504
H 11.61423  5.47621  3.39452
C 11.06761 -1.53969  3.75580
H 11.68563 -2.10513  3.06259
N  9.24405 -0.05392  4.16124
N  7.20706 -0.02544  2.17651
N  9.56204  1.72560  1.85663
N 10.89969  1.84669 -0.65199
N  9.20968  3.29393  4.30701
N  7.17477  3.39903  2.32668
Co  8.03265  1.65132  3.33984
C  5.83661  1.50204  5.55952
N  6.66710  1.56564  4.74040
C  4.79914  1.41877  6.57994
H  5.23719  1.25193  7.57818
H  4.21239  2.35112  6.61614
H  4.10891  0.58590  6.36761
```

1'-Co+2e⁻ (S = 1/2, 5-coordinates)

```
Energy              = -2791.754910
Enthalpy 0K         = -2791.289685
Free Energy 298K    = -2791.355745

N  7.28342  3.29069  2.70080
C  7.93831  4.21753  1.94513
C  7.20952  5.17186  1.21904
C  5.81714  5.22811  1.30794
C  5.16632  4.32129  2.15905
C  5.94106  3.38339  2.82203
C  9.49093  4.19597  1.95213
C 10.02391  5.43798  1.21469
Co  8.39625  1.65493  3.36324
N  9.58364  3.22892  4.26053
C  9.97973  4.22334  3.43006
C 10.80819  5.25210  3.91259
C 11.21814  5.26730  5.24520
C 10.78719  4.24388  6.08916
C  9.97443  3.25560  5.54514
C 10.02050  2.91955  1.25092
C 10.81766  2.94310  0.11345
N 11.30173  1.82961 -0.47879
C 10.92955  0.66100  0.08506
C 10.11763  0.57864  1.20969
N  9.67610  1.72231  1.81053
C  9.62211 -0.76039  1.80859
C  9.90949 -0.86350  3.33382
C 10.74917 -1.85773  3.86090
C 10.90974 -2.00321  5.24024
C 10.19661 -1.15176  6.08642
C  9.40547 -0.17081  5.50407
N  9.28826  0.00993  4.17111
C  8.08738 -0.83214  1.57856
C  7.50886 -1.80252  0.74171
C  6.12969 -1.83204  0.54055
C  5.33915 -0.87274  1.18304
C  5.97965  0.05898  1.99016
N  7.30752  0.09203  2.19260
C 10.31249 -1.93032  1.08462
H  8.12620 -2.54556  0.24251
H 11.10341  3.88385 -0.36085
H  8.82385  0.51064  6.13030
H  5.39559  0.82578  2.50229
H  5.47278  2.66475  3.50107
H  4.25392 -0.84609  1.06568
H 10.10182 -1.90469  0.00969
H  9.97199 -2.89962  1.47289
H 11.40198 -1.87251  1.20182
H 11.07122  4.20525  7.14283
H 11.56338 -2.78166  5.64048
H  9.62212  2.43462  6.17241
H  5.68305 -2.59241 -0.10460
H 11.31428 -0.23575 -0.40446
H 10.24987 -1.24034  7.17354
H 11.12078  5.45438  1.21820
H  9.66093  6.36613  1.67615
H  9.70502  5.43458  0.16589
H  4.08439  4.34273  2.30539
H  5.25698  5.97678  0.74354
H  7.72103  5.89241  0.58505
H 11.86128  6.07015  5.61399
H 11.14061  6.05078  3.25485
H 11.28025 -2.53846  3.20049
```

1'-Co+2e^- (S = 3/2)

Energy	=	-2924.426051
Enthalpy 0K	=	-2923.914045
Free Energy 298K	=	-2923.992088

C	7.39307	-1.78633	0.71178
H	8.00572	-2.47120	0.13158
C	7.98258	-0.86217	1.59526
C	10.90840	2.94318	0.31380
H	11.28282	3.88748	-0.08442
C	7.92912	4.29437	1.82464
C	10.02017	0.58729	1.25683
C	9.62186	-0.19091	5.47165
H	9.09505	0.49276	6.14113
C	5.87121	-0.08328	2.16949
H	5.29627	0.60650	2.79147
C	5.83335	3.42770	2.32690
H	5.27213	2.67378	2.88358
C	5.21890	-0.97864	1.33033
H	4.12809	-0.99385	1.27773
C	10.18387	-1.92519	0.99497
H	9.95097	-1.85341	-0.07343
H	9.84211	-2.90489	1.35708
H	11.27527	-1.88686	1.08627
C	9.85056	4.23365	3.50960
C	10.45975	4.21654	6.22057
H	10.64982	4.17676	7.29534
C	9.90332	-0.91405	3.28257
C	9.46936	4.22514	2.00540
C	9.99591	2.91955	1.35947
C	11.14686	-1.98091	5.08092
H	11.86440	-2.73178	5.42109
C	9.58375	3.31579	5.62639
H	9.06976	2.56680	6.23302
C	6.00736	-1.84713	0.57162
H	5.55457	-2.57057	-0.11099
C	10.93110	0.67480	0.21315
H	11.32309	-0.22289	-0.26749
C	9.52091	-0.78047	1.78458
C	10.51599	-1.12315	5.98488
H	10.70704	-1.17413	7.05900
C	10.10840	5.44776	1.32004
H	11.20047	5.42258	1.40761
H	9.74854	6.38504	1.76690
H	9.87455	5.46569	0.24960
C	5.16298	4.38410	1.57350
H	4.07196	4.38411	1.52322
C	5.93369	5.33285	0.89754
H	5.46633	6.10766	0.28467
C	7.32060	5.28481	1.03045
H	7.91893	6.03159	0.51509
C	11.07388	5.16228	5.39634
H	11.77691	5.89396	5.80223
C	10.76112	5.16826	4.03771
H	11.22886	5.91330	3.39929
C	10.83263	-1.87356	3.72691
H	11.31425	-2.54999	3.02562
N	9.33291	-0.06773	4.16689
N	7.20546	-0.00464	2.29156
N	9.52782	1.72434	1.85722
N	11.39934	1.83808	-0.28612
N	9.29714	3.30152	4.31525
N	7.16910	3.36290	2.44047
Co	8.14019	1.64314	3.42633
C	6.06359	1.50206	5.78884
N	6.83197	1.55802	4.91239
C	5.10494	1.43038	6.88464
H	5.61926	1.27934	7.84800
H	4.51967	2.36176	6.95111
H	4.40506	0.59212	6.73592

1'-Co+2e^- (S = 3/2, 5-coordinates)

Energy	=	-2791.757717
Enthalpy 0K	=	-2791.291894
Free Energy 298K	=	-2791.357768

N	7.20676	3.27949	2.59367
C	7.88565	4.22379	1.89251
C	7.17824	5.20144	1.17329
C	5.78428	5.25401	1.22046
C	5.10774	4.32398	2.01325
C	5.86355	3.36460	2.67360
C	9.43914	4.20529	1.93888
C	9.98060	5.45324	1.21800
Co	8.27467	1.64768	3.28260
N	9.46019	3.23354	4.24257
C	9.91352	4.21086	3.41920
C	10.81281	5.17830	3.90117
C	11.23125	5.15845	5.23090
C	10.74069	4.15486	6.07054
C	9.86456	3.22525	5.52456
C	10.00423	2.93232	1.26528
C	10.90981	2.95921	0.21406
N	11.43768	1.84949	-0.34758
C	11.01985	0.67939	0.18395
C	10.11386	0.59090	1.23142
N	9.59529	1.73214	1.78540
C	9.59735	-0.75093	1.80303
C	9.84510	-0.84767	3.33458
C	10.70310	-1.81396	3.88566
C	10.83581	-1.94578	5.26888
C	10.08267	-1.10815	6.09432
C	9.27810	-0.15318	5.48699
N	9.18305	0.01093	4.15206
C	8.07134	-0.82833	1.52054
C	7.53219	-1.78261	0.63972
C	6.16112	-1.82447	0.39133
C	5.33768	-0.89483	1.03297
C	5.93908	0.02525	1.88223
N	7.26050	0.07526	2.12515
C	10.31345	-1.91913	1.10205
H	8.17501	-2.50562	0.14370
H	11.24587	3.90388	-0.21848
H	8.66433	0.51646	6.09524
H	5.32665	0.76636	2.39883
H	5.37531	2.62405	3.31269
H	4.25592	-0.88187	0.88452
H	10.14714	-1.88704	0.01942
H	9.95752	-2.88952	1.47442
H	11.39727	-1.86279	1.26067
H	11.02486	4.09189	7.12301
H	11.50015	-2.70458	5.68897
H	9.46317	2.42619	6.15036
H	5.74631	-2.57431	-0.28659
H	11.44862	-0.21631	-0.27029
H	10.11466	-1.18880	7.18287
H	11.07604	5.47372	1.24071
H	9.61069	6.37793	1.68196

```
H  9.68335  5.45260  0.16228
H  4.02112  4.33799  2.12043
H  5.24261  6.02207  0.66345
H  7.70766  5.94074  0.57797
H 11.92273  5.91859  5.60235
H 11.19097  5.95947  3.24662
H 11.26767 -2.48338  3.24176
```

2'-Co+2e⁻ (S = 1/2)

```
Energy             = -2924.418746
Enthalpy 0K        = -2923.908671
Free Energy 298K   = -2923.986856
```

```
C  9.26288  3.01334  6.82118
H 10.09468  2.40916  7.19001
C  9.28924  3.52003  5.52667
H 10.13467  3.31241  4.86756
C  8.19216  4.28124  5.10829
H  8.13787  4.68362  4.09360
C  7.16830  4.53260  6.01764
H  6.32693  5.14253  5.69806
C  7.22340  4.00820  7.32478
C  6.17787  4.39706  8.40454
C  5.22191  5.45528  7.81822
H  4.54860  5.85045  8.58714
H  4.60794  5.03166  7.01163
H  5.78079  6.30706  7.41310
C  6.94896  4.99015  9.60467
C  6.87056  6.34167  9.95798
H  6.22262  7.02208  9.40069
C  8.36886  6.05369 11.63800
H  8.95335  6.46630 12.46884
C  8.47467  4.71830 11.31212
H  9.15280  4.06843 11.87233
C  5.35147  3.18120  8.89868
C  3.96369  3.27975  9.06428
H  3.43261  4.20121  8.84346
C  3.24685  2.17265  9.51726
H  2.16300  2.22715  9.64401
C  3.93288  0.99440  9.79903
H  3.37544  0.12802 10.14531
C  5.32381  0.95422  9.64366
C  8.37534  1.16906 12.52662
H  8.95806  2.09140 12.56642
C  8.37986  0.28400 13.59867
H  8.96050  0.51075 14.49555
C  6.89473 -1.09950 12.31564
H  6.30468 -2.00821 12.22216
C  6.92401 -0.14908 11.28349
C  6.12397 -0.33886  9.96701
C  5.14500 -1.51913 10.12970
H  4.49411 -1.61697  9.25145
H  4.50251 -1.38683 11.00919
H  5.68545 -2.46604 10.25204
C  7.13224 -0.66081  8.83679
C  6.96822 -1.75934  7.97843
H  6.07357 -2.37659  8.02898
C  7.96006 -2.08593  7.05208
H  7.83318 -2.94038  6.38344
C  9.12712 -1.30806  7.02616
H  9.95656 -1.53836  6.35402
C  9.20055 -0.21543  7.87807
H 10.08851  0.42061  7.88548
N  8.25743  3.20960  7.68570
N  7.76731  4.14065 10.30462
N  6.00404  2.04229  9.21195
N  7.67799  0.96550 11.39403
N  8.22182  0.14159  8.73138
Co  8.11937  2.12410  9.59094
C  7.62385 -0.88498 13.48891
H  7.59244 -1.61665 14.29978
N  7.55102  6.90474 10.96844
C 11.24827  2.41377 10.18353
N 10.11904  2.24172  9.96823
C 12.65767  2.65955 10.46222
H 13.25737  2.57086  9.54257
H 12.79296  3.67481 10.86765
H 13.03939  1.93425 11.19779
```

2'-Co+2e⁻ (S = 1/2, 5-coordinates)

```
Energy             = -2791.756947
Enthalpy 0K        = -2791.291447
Free Energy 298K   = -2791.356060
```

```
N  7.81458  6.40534 11.38747
C  6.94870  5.97938 10.45031
C  7.07059  4.78631  9.73731
N  8.10946  3.92131 10.02424
C  9.04329  4.40012 10.90373
C  8.89329  5.59913 11.56307
C  6.14738  4.42363  8.54339
C  5.29637  3.14540  8.80830
C  3.90449  3.13357  8.63548
C  3.19665  1.95746  8.86586
C  3.87706  0.81332  9.27543
C  5.26607  0.87404  9.45126
N  5.92717  2.02101  9.20609
C  6.09144 -0.35317  9.94276
C  7.20664 -0.75332  8.93625
C  7.26596 -2.02391  8.35257
C  8.35617 -2.40114  7.56062
C  9.40759 -1.48010  7.40457
C  9.28199 -0.23232  7.98642
N  8.19373  0.16546  8.69906
Co  7.94140  2.02594  9.38103
N  7.65064  1.04491 11.28882
C  8.30208  1.36000 12.42110
C  8.15307  0.65803 13.61134
C  7.27547 -0.43006 13.62519
C  6.59777 -0.76038 12.45216
C  6.79871 -0.00602 11.28358
C  7.08349  4.18900  7.33026
C  7.16025  5.09174  6.25595
C  8.04669  4.85630  5.20535
C  8.85280  3.71425  5.24753
C  8.72772  2.87268  6.34707
N  7.87813  3.09626  7.36172
C  5.19741  5.59915  8.24562
C  5.13803 -1.54252 10.15603
H  9.33071  1.96380  6.41702
H  9.55707  3.47661  4.44762
H  8.10110  5.55312  4.36536
H  6.53300  5.97985  6.22625
H  4.50012  5.76083  9.07753
H  4.60911  5.41839  7.33595
H  5.75755  6.53018  8.10442
H  6.10507  6.64839 10.26381
H  9.66461  5.93749 12.26482
```

```
H  9.93216  3.78173 11.06511
H  3.36595  4.02325  8.32083
H  2.11290  1.93224  8.72740
H  3.31808 -0.10151  9.45223
H  8.95952  2.23064 12.36860
H  8.70372  0.96519 14.50261
H  5.91307 -1.60507 12.46231
H  4.62904 -1.81146  9.22161
H  4.36938 -1.30715 10.90340
H  5.68253 -2.42849 10.50342
H  6.46714 -2.74495  8.51299
H  8.39254 -3.39004  7.09970
H 10.30684 -1.72724  6.83615
H 10.08114  0.50772  7.88727
H  7.11440 -1.01248 14.53558
```

2'-Co+2e$^-$ (S = 3/2, 5-coordinates)

```
Energy              = -2791.755476
Enthalpy 0K         = -2791.290020
Free Energy 298K    = -2791.354878

N  7.72456  6.56695 11.23977
C  6.92045  6.09865 10.27192
C  7.02833  4.83476  9.68546
N  7.98988  3.95116 10.13605
C  8.85667  4.45797 11.06424
C  8.72595  5.71952 11.59762
C  6.16093  4.39125  8.48231
C  5.30929  3.14672  8.85845
C  3.91159  3.20013  8.93132
C  3.19165  2.05637  9.27342
C  3.88282  0.87944  9.57100
C  5.27890  0.87891  9.51492
N  5.95526  1.99165  9.15363
C  6.11555 -0.37147  9.91443
C  7.20379 -0.72694  8.86289
C  7.18974 -1.94205  8.16252
C  8.24793 -2.29340  7.32114
C  9.33480 -1.41955  7.22262
C  9.27551 -0.22126  7.91869
N  8.22358  0.15563  8.68093
Co  7.96953  2.03132  9.39138
N  7.66600  1.00334 11.28777
C  8.28057  1.32236 12.43959
C  8.11849  0.60685 13.62068
C  7.26991 -0.50255 13.60473
C  6.61976 -0.83081 12.41498
C  6.82896 -0.05826 11.26063
C  7.14662  4.10091  7.31673
C  7.14740  4.87070  6.13900
C  8.10194  4.65323  5.14744
C  9.07047  3.66637  5.35591
C  9.00751  2.94054  6.53863
N  8.07549  3.12699  7.48770
C  5.21350  5.53467  8.07308
C  5.17397 -1.57636 10.09622
H  9.74130  2.15543  6.73147
H  9.85023  3.45643  4.62099
H  8.08896  5.24751  4.23056
H  6.40175  5.64578  5.98159
H  4.54325  5.80264  8.89795
H  4.59549  5.24944  7.21039
H  5.77621  6.43763  7.81036
H  6.13871  6.78994  9.94804
```

```
H  9.44524  6.07690 12.34358
H  9.68905  3.81473 11.36684
H  3.37104  4.11711  8.71340
H  2.10001  2.07978  9.30690
H  3.32168 -0.01141  9.84116
H  8.92236  2.20432 12.41248
H  8.64427  0.91677 14.52595
H  5.95317 -1.68992 12.40064
H  4.61396 -1.77959  9.17419
H  4.44350 -1.39335 10.89304
H  5.73368 -2.48220 10.36157
H  6.35856 -2.63468  8.27022
H  8.22989 -3.23954  6.77583
H 10.21171 -1.65720  6.61679
H 10.10805  0.48480  7.87390
H  7.10965 -1.10321 14.50330
```

2'-Co+2e$^-$ (S = 3/2)

```
Energy              = -2924.420980
Enthalpy 0K         = -2923.909953
Free Energy 298K    = -2923.989904

C  9.28716  3.01420  6.77435
H 10.12520  2.41406  7.13602
C  9.29987  3.52289  5.48018
H 10.13973  3.31882  4.81264
C  8.20118  4.28608  5.07634
H  8.13679  4.69192  4.06355
C  7.18722  4.53683  5.99805
H  6.34475  5.15027  5.68900
C  7.25282  4.00654  7.30212
C  6.21018  4.38559  8.38909
C  5.25777  5.45069  7.80982
H  4.57242  5.83205  8.57379
H  4.65195  5.03513  6.99295
H  5.81917  6.31128  7.42503
C  6.95502  4.95255  9.61626
C  6.77176  6.26529 10.07480
H  6.09037  6.94162  9.55340
C  8.24454  5.94822 11.77176
H  8.77987  6.33608 12.64611
C  8.45387  4.65365 11.34436
H  9.16883  4.01222 11.86683
C  5.38468  3.17392  8.89027
C  4.01009  3.28962  9.11961
H  3.48243  4.21698  8.91364
C  3.29315  2.19715  9.60976
H  2.21512  2.26396  9.77166
C  3.98248  1.00906  9.87181
H  3.43258  0.15112 10.25050
C  5.35618  0.94676  9.63625
C  8.40718  1.17807 12.49067
H  9.06504  2.04930 12.48961
C  8.28869  0.38966 13.62906
H  8.84593  0.64122 14.53379
C  6.76086 -0.97829 12.37949
H  6.10437 -1.84394 12.32708
C  6.92295 -0.12683 11.27185
C  6.15667 -0.34836  9.94189
C  5.18300 -1.53358 10.09795
H  4.54604 -1.63905  9.21034
H  4.52195 -1.39729 10.96119
H  5.72696 -2.47611 10.24490
C  7.16690 -0.67674  8.80952
```

```
C  7.00120 -1.79224  7.96900
H  6.11530 -2.41845  8.04074
C  7.97832 -2.11665  7.02777
H  7.84569 -2.98094  6.37227
C  9.12879 -1.32946  6.95967
H  9.94242 -1.55613  6.26739
C  9.21046 -0.22768  7.80468
H 10.09145  0.41726  7.79083
N  8.28973  3.20838  7.64874
N  7.82335  4.11552 10.27054
N  6.04446  2.01963  9.16799
N  7.75594  0.93745 11.34165
N  8.24856  0.11935  8.67404
Co  8.17690  2.10384  9.53314
C  7.44457 -0.72510 13.56738
H  7.31820 -1.38653 14.42795
N  7.38997  6.79093 11.13787
C 11.28944  2.41388 10.11017
N 10.15933  2.23326  9.89776
C 12.69717  2.67071 10.38818
H 13.30279  2.56014  9.47451
H 12.83036  3.69615 10.76842
H 13.07883  1.96602 11.14394
```

3'-Co+2e⁻ (S = 1/2)

```
Energy              = -2940.442323
Enthalpy 0K         = -2939.942839
Free Energy 298K    = -2940.021143
```

```
C  3.99668  7.85961  3.30437
H  3.47568  6.96155  2.96817
C  3.31839  8.83986  4.01445
H  2.25965  8.71261  4.25004
C  4.03117  9.97522  4.41551
H  3.55038 10.77579  4.98225
C  5.38156 10.06490  4.07340
H  5.93742 10.94936  4.37528
C  6.00370  9.03049  3.35641
C  7.49424  9.10699  2.92891
C  8.12677 10.38561  3.51134
H  8.03495 10.41368  4.60396
H  7.63601 11.28269  3.11463
H  9.19364 10.44997  3.26265
C  7.54799  9.17384  1.37846
C  8.18947 10.22558  0.70163
H  8.72583 10.99775  1.24802
C  8.14762 10.29884 -0.69024
H  8.65348 11.11225 -1.21554
C  7.43466  9.31760 -1.38828
H  7.34726  9.33431 -2.47657
C  6.84145  8.29907 -0.65539
H  6.27942  7.51089 -1.15906
C  8.26713  7.86408  3.46220
C  9.25892  8.00797  4.44280
H  9.51738  8.97862  4.85580
C  9.92611  6.87812  4.90160
H 10.69993  6.96613  5.66834
C  9.60026  5.63593  4.37150
H 10.12560  4.75616  4.73016
C  8.60168  5.53869  3.38863
C  7.26195  4.82707 -0.74622
H  6.51988  5.43653 -1.26804
C  8.05874  3.94650 -1.45216
H  7.96071  3.85721 -2.54004
C  9.04015  3.29593  0.48717
H  9.77417  2.65595  0.98064
C  8.23463  4.16828  1.23834
C  8.22168  4.15705  2.78452
C  9.21445  3.09605  3.30083
H  9.00301  2.11573  2.86128
H  9.14811  2.98584  4.39049
H 10.24792  3.36089  3.04076
C  6.78237  3.76253  3.18252
C  6.47215  2.51423  3.74093
H  7.26316  1.80021  3.97724
C  4.25253  2.98353  3.71291
H  3.21854  2.68745  3.92523
C  4.52397  4.21541  3.15033
H  3.70751  4.89673  2.89836
N  5.30356  7.93292  2.98215
N  6.91331  8.19919  0.68487
N  7.95535  6.64842  2.97084
N  7.35560  4.99259  0.59288
N  8.99244  3.18162 -0.84342
N  5.78329  4.64491  2.89103
N  5.22980  2.10729  4.03322
Co  6.23349  6.47454  1.73115
C  3.64245  5.85492 -0.11713
N  4.54268  6.17813  0.54016
C  2.51865  5.41868 -0.93642
H  1.74051  6.19663 -0.97270
H  2.85261  5.20744 -1.96422
H  2.08012  4.49935 -0.51787
```

3'-Co+2e⁻ (S = 1/2, 5-coordinates)

```
Energy              = -2807.778325
Enthalpy 0K         = -2807.324419
Free Energy 298K    = -2807.389152
```

```
N  5.27329  2.24280  4.24039
C  6.50758  2.59603  3.87458
C  6.82279  3.81985  3.26058
N  5.83058  4.71617  3.00214
C  4.57979  4.34853  3.35247
C  4.30381  3.13664  3.96184
C  8.24759  4.16889  2.77849
C  9.24151  3.08831  3.24606
Co  6.42404  6.48158  1.77074
N  7.22756  4.98372  0.63799
C  6.99480  4.76606 -0.68549
C  7.72098  3.85736 -1.42649
N  8.71048  3.10922 -0.88441
C  8.87689  3.25976  0.43578
C  8.15222  4.15737  1.23059
C  8.69364  5.54344  3.35962
C  9.77124  5.65297  4.25116
C 10.11362  6.90086  4.76339
C  9.38686  8.02471  4.37991
C  8.32218  7.87138  3.48226
N  8.00680  6.65077  3.00827
C  7.48382  9.08660  2.98374
C  7.62291  9.21506  1.44034
C  8.12545 10.37653  0.83042
C  8.20614 10.46073 -0.55934
C  7.77675  9.37218 -1.32337
C  7.29522  8.25435 -0.65304
N  7.21746  8.16722  0.68655
C  5.96781  8.92253  3.29114
```

```
C  5.26186  9.84644  4.07325
C  3.87667  9.74088  4.22665
C  3.21194  8.70766  3.55087
C  3.96825  7.81374  2.81169
N  5.31820  7.87680  2.70548
C  8.00758 10.36708  3.65834
H  3.48621  6.99426  2.27265
H  2.12732  8.59230  3.60230
H  3.33156 10.45766  4.84416
H  5.77896 10.66473  4.56914
H  7.91456 10.30338  4.75005
H  7.44443 11.24780  3.32707
H  9.06506 10.53823  3.42047
H  8.45983 11.22225  1.42647
H  8.60110 11.36209 -1.03394
H  7.82019  9.38140 -2.41422
H  6.96800  7.37461 -1.21041
H  9.65955  8.99619  4.78309
H 10.94871  6.99806  5.46148
H 10.34538  4.78188  4.55345
H  6.19420  5.34957 -1.15041
H  7.51214  3.72312 -2.49374
H  9.64507  2.62406  0.88113
H  8.97534  2.10630  2.84088
H  9.25036  3.00259  4.34031
H 10.26088  3.31928  2.90961
H  7.28870  1.86537  4.09278
H  3.27790  2.87792  4.24610
H  3.77606  5.06027  3.14954
```

3'-Co+2e⁻ (S = 3/2)

```
Energy             = -2940.444296
Enthalpy 0K        = -2939.944425
Free Energy 298K   = -2940.021618

C  4.01508  7.82693  3.40291
H  3.51651  6.89059  3.14658
C  3.32479  8.83525  4.06584
H  2.27872  8.69326  4.34499
C  4.00940 10.01804  4.35349
H  3.51484 10.84702  4.86546
C  5.34847 10.12227  3.97727
H  5.88512 11.04146  4.20035
C  5.98389  9.05329  3.32309
C  7.47853  9.11387  2.90801
C  8.11754 10.39844  3.47194
H  7.96978 10.47606  4.55577
H  7.67817 11.29357  3.01304
H  9.19842 10.41827  3.28326
C  7.57566  9.13680  1.35878
C  8.34516 10.09478  0.67673
H  8.94583 10.82147  1.21859
C  8.34810 10.12695 -0.71796
H  8.95125 10.86660 -1.25003
C  7.55598  9.20993 -1.41032
H  7.49846  9.20665 -2.50069
C  6.83734  8.28000 -0.66692
H  6.20549  7.54139 -1.16286
C  8.20956  7.87098  3.49133
C  9.08368  7.99880  4.57689
H  9.28498  8.96234  5.03762
C  9.71138  6.86095  5.07670
H 10.40051  6.93448  5.92155
C  9.45918  5.63115  4.47732
H  9.95412  4.74500  4.86348
C  8.57984  5.55432  3.38614
C  7.20233  4.87365 -0.78386
H  6.42714  5.46191 -1.28125
C  8.00284  4.01795 -1.51561
H  7.87782  3.93098 -2.60108
C  9.04797  3.38175  0.39348
H  9.81020  2.75708  0.86258
C  8.24387  4.23134  1.17381
C  8.26143  4.18864  2.72042
C  9.29640  3.14811  3.19382
H  9.11942  2.17598  2.72135
H  9.23902  2.99073  4.27731
H 10.31782  3.46646  2.94326
C  6.84191  3.75593  3.14016
C  6.56892  2.50910  3.72063
H  7.38128  1.81210  3.93765
C  4.34280  2.94338  3.75348
H  3.32058  2.63162  3.99768
C  4.57503  4.17136  3.16839
H  3.73780  4.83102  2.92525
N  5.30278  7.92428  3.03150
N  6.86828  8.21576  0.67363
N  7.96121  6.66724  2.93362
N  7.33145  5.04020  0.55249
N  8.97264  3.27763 -0.93623
N  5.81867  4.62083  2.86156
N  5.34491  2.08287  4.05187
Co  6.19948  6.44872  1.72631
C  3.58748  5.87890 -0.04213
N  4.50302  6.18284  0.60612
C  2.44618  5.46322 -0.84790
H  1.64512  6.21777 -0.80724
H  2.74489  5.32708 -1.89924
H  2.04747  4.50655 -0.47515
```

3'-Co+2e⁻ (S = 3/2, 5-coordinates)

```
Energy             = -2807.778830
Enthalpy 0K        = -2807.324358
Free Energy 298K   = -2807.389113

N  5.24335  2.27795  4.17803
C  6.48807  2.65727  3.87655
C  6.81447  3.85593  3.22355
N  5.81760  4.72584  2.86576
C  4.55229  4.31279  3.12796
C  4.27044  3.12304  3.76909
C  8.25591  4.19739  2.78319
C  9.23321  3.11887  3.28865
Co  6.42023  6.48593  1.77130
N  7.32840  5.02571  0.60438
C  7.08053  4.76704 -0.70862
C  7.73741  3.77800 -1.40989
N  8.67139  2.98486 -0.83471
C  8.84114  3.17276  0.47931
C  8.18486  4.15500  1.23499
C  8.69613  5.57286  3.35800
C  9.77030  5.69089  4.25323
C 10.10972  6.94040  4.76347
C  9.37974  8.06124  4.37601
C  8.31944  7.90001  3.47594
N  8.00519  6.67873  3.00176
C  7.47787  9.11205  2.97311
C  7.60185  9.22568  1.42661
```

C	8.14001	10.36519	0.80506
C	8.21904	10.43334	-0.58564
C	7.75450	9.35334	-1.33731
C	7.23771	8.25782	-0.65480
N	7.16111	8.18632	0.68501
C	5.97191	8.93958	3.31916
C	5.28736	9.85648	4.13163
C	3.91618	9.72029	4.35361
C	3.24282	8.66607	3.73089
C	3.98188	7.78599	2.95333
N	5.31559	7.88764	2.76774
C	8.00781	10.40097	3.62659
H	3.49173	6.95129	2.44890
H	2.16672	8.52211	3.84622
H	3.38657	10.43263	4.99008
H	5.81080	10.68832	4.59657
H	7.92681	10.35178	4.71971
H	7.44267	11.27838	3.28835
H	9.06352	10.56657	3.37835
H	8.50271	11.20586	1.39134
H	8.63933	11.31810	-1.06977
H	7.79378	9.35118	-2.42833
H	6.87885	7.38590	-1.20345
H	9.64936	9.03500	4.77606
H	10.94496	7.04167	5.46078
H	10.34710	4.82248	4.55772
H	6.32663	5.38706	-1.20352
H	7.52325	3.61917	-2.47279
H	9.55935	2.50105	0.95374
H	8.95601	2.12906	2.91245
H	9.23184	3.06408	4.38481
H	10.25767	3.32778	2.95221
H	7.27423	1.96466	4.18433
H	3.23258	2.83793	3.97430
H	3.73789	4.97221	2.81954

Reference:

1. S. Grimme, *Chem.-Eur. J.*, 2012, **18**, 9955.
